# Models of provider care in long-term care: A rapid scoping review

**DOI:** 10.1371/journal.pone.0254527

**Published:** 2021-07-16

**Authors:** Candyce Hamel, Chantelle Garritty, Mona Hersi, Claire Butler, Leila Esmaeilisaraji, Danielle Rice, Sharon Straus, Becky Skidmore, Brian Hutton

**Affiliations:** 1 Knowledge Synthesis Group, Ottawa Hospital Research Institute, Ottawa, Ontario, Canada; 2 Department of Medicine, University of Toronto and St. Michael’s Hospital, Toronto, Ontario, Canada; 3 School of Epidemiology and Public Health, University of Ottawa, Ottawa, Ontario, Canada; Witten/Herdecke University, GERMANY

## Abstract

**Introduction:**

One of the current challenges in long-term care homes (LTCH) is to identify the optimal model of care, which may include specialty physicians, nursing staff, person support workers, among others. There is currently no consensus on the complement or scope of care delivered by these providers, nor is there a repository of studies that evaluate the various models of care. We conducted a rapid scoping review to identify and map what care provider models and interventions in LTCH have been evaluated to improve quality of life, quality of care, and health outcomes of residents.

**Methods:**

We conducted this review over 10-weeks of English language, peer-reviewed studies published from 2010 onward. Search strategies for databases (e.g., MEDLINE) were run on July 9, 2020. Studies that evaluated models of provider care (e.g., direct patient care), or interventions delivered to facility, staff, and residents of LTCH were included. Study selection was performed independently, in duplicate. Mapping was performed by two reviewers, and data were extracted by one reviewer, with partial verification by a second reviewer.

**Results:**

A total of 7,574 citations were screened based on the title/abstract, 836 were reviewed at full text, and 366 studies were included. Studies were classified according to two main categories: healthcare service delivery (n = 92) and implementation strategies (n = 274). The condition/ focus of the intervention was used to further classify the interventions into subcategories. The complex nature of the interventions may have led to a study being classified in more than one category/subcategory.

**Conclusion:**

Many healthcare service interventions have been evaluated in the literature in the last decade. Well represented interventions (e.g., dementia care, exercise/mobility, optimal/appropriate medication) may present opportunities for future systematic reviews. Areas with less research (e.g., hearing care, vision care, foot care) have the potential to have an impact on balance, falls, subsequent acute care hospitalization.

## Introduction

On a global level, the population is ageing. In 2020, approximately 9%, or over 700,000,000, of the global population were aged 65 years and older [1, 2]. By 2050, one in six people (over 1.5 billion people; 16%) in the world will be over 65 [3, 4]. Between 2010–2050, it is projected that the 85-and-over population will increase by 351%. A cause for concern, as the prevalence of dementia rises with age, with an estimated 25–30% of people 85 years and older having dementia [4].

There has been a shift in leading causes of disease and death, moving from infectious and acute disease to chronic and degenerative diseases [4]. Due to declining health and with the development of multiple chronic diseases, many older adults need assistance with activities of daily living (ADL), such as bathing or preparing a meal. More generally, they may also require effective and innovative support and management for complex medical and social needs [5]. Requiring such help may lead to admission to long-term care homes (LTCH). LTCH (also called nursing homes) provide living accommodation for older adults who require on-site delivery of 24-hour, seven days a week supervised care, including professional health services, personal care and services such as meals, laundry and housekeeping [6]. In 2020, there were 18,075 care homes across the UK, with over half a million adult care home residents [7]. In 2016, there were over 15,600 Medicare- or Medicare-certified nursing homes in the United States [8]. In 2012, 143,000 Canadian lived in approximately 1,360 LTCHs across the country [9]. In Canada, as of 2014, these homes employed more than 126,000 full-time employees. Direct care is provided by care aides/personal support workers (PSW), registered nurses (RN), registered practice nurses (RPN), as well as allied health professionals (e.g., physiotherapists, occupational therapists). Over the years, there has been a marked decline in regulated caregivers in Canadian LTCHs [10, 11], with unregulated care aides (e.g., PSW) providing almost 90% of the direct care [12]. Some provinces, including Alberta, have recently started initiatives to regulate these care providers [13].

COVID-19 deaths in LTCHs often represent a large proportion of overall deaths from COVID-19, an average of 38% in Organisation for Economic Co-operation and Development (OECD) countries [14]. In the UK, of the deaths registered as related to COVID-19, 31% (n = 17,127) occurred in care homes [7]. Although Canada’s overall mortality rate from COVID-19 is relatively low, LTCH residents accounted for 81% of all reported COVID-19 deaths [14]. The troubling spread of COVID-19 through LTCHs across Canada has highlighted issues LTCH industry faces about how to operate and provide care. In June 2020, the Royal Society of Canada (RSC) released a policy briefing entitled, *‘Restoring Trust*: *COVID-19 and The Future of Long-Term Care*,*’* developed by the Working Group on Long-Term Care in Canada [12]. This report is an incontestable overview of the long-standing challenges in the LTC sector and their causes. It also highlights the characteristics of older Canadians living in LTCHs, their caregivers and the physical environment of these homes. Importantly, this report focused on the healthcare workforce and proposed nine recommended steps to solving the workforce crisis in LTCH including identification and implementation of optimal care models.

Existing systematic reviews have focused on one of two main areas: (1) Evaluating the impact of specific healthcare providers (e.g., pharmacists [15], specialist practitioners [16, 17], physiotherapists [18]) in LTCHs. For example, Barker (2018) found that the addition of a specialist practitioner, either a doctor or nurse, to supplement usual primary care, has the potential to improve health outcomes for LTCH residents [16]; or (2) Evaluating the impact of interventions specific to health conditions, which may include several healthcare providers within the model, for example nonpharmalogical interventions for dementia [19, 20]. However, we know that healthcare is provided by a range of professionals (e.g., personal support workers, physiotherapists, pharmacists, occupational therapists, psychologists) working together, and that residents in LTCHs do not typically have only one condition (e.g., cognitive decline, depression, urinary incontinence). Unfortunately, little is known about the optimal *mix* of healthcare provider groups to achieve the best outcomes for residents when delivering care and there is no consensus on the complement or scope of care delivered by these providers.

### Objectives

In June 2020, the Royal Society of Canada (RSC) release a policy briefing on COVID-19 and the future of LTC in Canada [12]. It highlighted the “profound, long-standing deficiencies in the long-term care sector that contributed to the magnitude of the COVID-19 crisis”. As an extension to this recent policy briefing, the RSC is motivated to better understand how to improve the healthcare for residents in LTCH. Therefore, on behalf of the RSC through the Strategy for Patient Oriented Research (SPOR) Evidence Alliance, we undertook a rapid scoping review. Scoping reviews are often conducted to: (1) Identify the types of available evidence in a given field; (2) Identify and analyze knowledge gaps; and (3) Inform future research, for example, as a precursor to a systematic review or to inform primary research where knowledge gaps exist [21, 22]. In order to produce the evidence for the RSC in a short time frame (i.e., 10 weeks), we employed rapid review methodologies to the conduct of this scoping review, through streamlining or omitting some of the methods (e.g., single data extraction with partial verification) [23, 24].

The objective of this rapid scoping review was to identify what care provider models and interventions in LTCHs have been evaluated to improve quality of life, quality of care, and health outcomes of residents, map these interventions, and identify gaps in the literature. This manuscript is a modified version of the full report (https://osf.io/bpxk4/), with a focus on interventions evaluating healthcare services delivery (further discussed in the Methods) in LTCH.

## Methods

A rapid scoping review protocol was prepared and registered on Open Science Framework (https://osf.io/u3an4/), and was guided by established scoping review [21] and rapid review methodology [25]. This project was conducted over a 10-week timeframe (July 10 to September 18, 2020) and was reported according to Preferred Reporting Items for Systematic Reviews and Meta-Analyses extension for scoping reviews (PRISMA-ScR) statement (S1 File) [26].

### Key questions

The focus was centered around the care provider perspective (i.e., providing the necessary staff levels, mix of staff, and interventions to the facility, staff, and residents). Specifically, what type/level of care (e.g., medical, direct patient care, allied health care) should be provided and by whom?

The following questions were addressed in this scoping review:

1. What care provider models or services in LTC homes have been evaluated to improve quality of life, quality of care, and health outcomes of residents? Care provider models encompass the makeup of the healthcare provider team (e.g., adding a nurse practitioner), and care provider services encompass an additional service provided by a new healthcare team member (e.g., monthly medication reviews performed by a pharmacist, bi-annual eye exams provided by an ophthalmologist).
2. What interventions delivered by care providers in LTC homes have been evaluated to improve quality of life, quality of care, and health outcomes of residents? These interventions may include exercise program delivered by physiotherapists, interventions for depression for patient with dementia delivered by mental healthcare providers.

### Inclusion and exclusion criteria

Table 1 provides a summary of the inclusion criteria.

**Table 1:** Inclusion/exclusion criteria.

PICO element	Details
**Population/ participants**	Residents of LTCH^1^ with any condition (e.g., frailty, dementia) Palliative care was limited to within LTCH *Excluded*: Hospice settings, residential homes, skilled nursing facilities
**Interventions/ exposure**	Models of provider care, or interventions delivered to facility, staff, and residents in LTCHs. Includes studies evaluating different approaches/ arrangements of staffing (separately or in combination): i ***Medical care*** provided by: physicians; physician assistant or nurse practitioner^2^; specialty physicians; palliative physicians. ii ***Direct patient care*** provided by: regulated nurses (e.g., registered nurses, licensed practical nurses), personal support workers, nursing aides. iii ***Allied health team care*** provided by: physical therapists, occupational therapists, speech/ language therapists, recreation therapists, dieticians, podiatry/chiropody, dental, vision care, hearing care, pharmacists, psychologists, and social workers along with those working as aides alongside these positions. Care also included spiritual care, palliative care, advanced care planning, psychosocial/ mental health services, cognitive training and those services specific to dementia care. Studies evaluating access to or direct services provided by relevant care providers to LTC residents. *Excluded*: art, music, pet, robot or virtual reality interventions; studies focused on continuing medical education or training/education as part of professional development requirements; indirect care (e.g., cleaning, food preparation)
**Comparator(s)/ Control(s)**	Different models of provider care Different models were compared over time at the same LTCH Limited to those conducted within or across LTCH
**Outcomes**	**Primary**: Quality of life; quality of care (e.g., urinary tract infection, pressure ulcers, use of antipsychotics); health outcomes (e.g., mortality; appropriateness of prescribing and number of medications; ER admissions/ hospitalizations) **Secondary:** Healthcare worker stress, burnout or quality of work-life if reported along with a relevant primary outcome *Excluded***:** Studies that specifically evaluated interventions to mitigate these healthcare worker outcomes
**Study designs**	Randomized controlled trials (RCTs); non-RCTs; quasi-experimental study designs (e.g., controlled before-after studies); comparative cohort studies *Excluded*: Cross-sectional, case-control, case reports and qualitative literature
**Geography**	No geographic restrictions
**Language**	English

^1^ LTCH, as defined by Health Canada [6], provide living accommodation for people who require on-site delivery of 24-hour, 7 days a week supervised care, including professional health services, personal care and services such as meals, laundry and housekeeping. In other countries, these homes may have other names (e.g., care homes, residential aged care facilities [RACFs]), but offer similar levels of medical care (e.g., physician, nurse) and other services (e.g., meals).

^2^ Both physician assistants and nurse practitioners can work autonomously within their scope of practice within primary care [27, 28].

### Description of methods

Table 2 provides a brief description of the methods, with complete methods described in S2 File.

**Table 2:** Methods in brief.

Review stage	Details
**Literature search**	Both research questions captured using a single search strategy MEDLINE, Embase, CENTRAL, PsycINFO, and CINAHL Published since 2010, for feasibility and to capture the most recently evaluated models of care and interventions No language limits were applied Peer reviewed with PRESS^a^ (S3 File) Search run on July 9, 2020 (S4 File) For feasibility, no grey literature searching or scanning of the reference lists of the included studies was performed
**Study selection**	Citations from literature search collated and de-duplicated in Reference Manager^b^, unique results uploaded to DistillerSR^®^^c^ Screening performed in two stages: (1) title and abstract; (2) full text, with pilot testing for each stage Screened independently, in duplicate with disagreements resolved through consensus DistillerSR’s^®^ artificial intelligence (AI) active-machine learning to implement prioritized screening for title and abstract records At 95% estimated recall, the AI reviewer was assigned to exclude the remaining records. A human reviewer screened all of the citations excluded by the AI reviewer, and any conflicts were resolved between two human reviewers.
**Data mapping/ charting**	Using standardized, and piloted, forms (S5 File), conducted in two phases: i Mapping: using guidance from Effective Practice and Organisation of Care (EPOC)^d^, interventions were mapped to one of two categories: 1) evaluating delivery of a healthcare service; or, 2) evaluating implementation of a healthcare strategy within LTCHs, and subcategories (further described in S2 File). Performed by two reviewers through discussion. ii Charting: two different charting forms depending on mapping. Extracted by one reviewer, with approximately 20% of data verified by a second reviewer.
**Risk of bias**	The objective was to identify and map interventions offered in LTCH, and not to evaluate the risk of bias of these studies. Therefore, risk of bias was not completed.
**Synthesis**	Descriptive approach, presented narratively and in tables Across studies involving similar care providers within a condition or focus of the intervention provided, we have highlighted consistent and/or contradictory conclusions.

^a^ McGowan et al. https://doi.org/10.1016/j.jclinepi.2016.01.021.

^b^ Thomson Reuters. Reference Manager 12.

^c^ Evidence Partners. DistillerSR [https://v2dis-prod.evidencepartners.com/]

^d^ Effective Practice and Organisation of Care (EPOC). The EPOC taxonomy of health systems interventions. EPOC Resources for review authors 2016.

## Results

### Search findings

**Fig 1:**
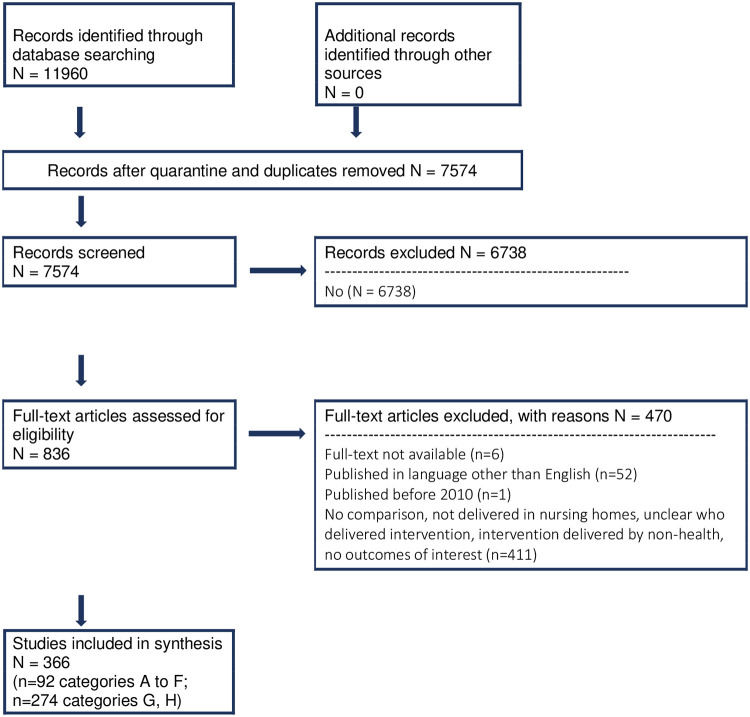
PRISMA flow diagram. Fig 1 presents the flow (inclusion/exclusion) of the studies through the stages of study selection.

The search resulted in 11,960 records. After removing duplicates (n = 2,369) and quarantining records based on title (e.g., cross-sectionals, systematic reviews, reviews, study and review protocols, trial registries, studies in children) (n = 2,017), 7,574 citations were screened based on the title and abstract. Using the AI ranking feature, the estimated recall of 95% (828/872) of included records was achieved after 4,128 records were screened. At this time, the highest prediction score that a citation was relevant was 0.1774 (or 17.74%), and the remaining 3,446 records were excluded by the AI reviewer. Human reviewers included nine of these records to be further reviewed at full text, seven because there was no abstract, and two because it was unclear if the intervention took place in a LTCH. Of these nine, all were excluded when evaluated at full-text. A total of 836 records were included to be further reviewed at full text and 366 of these studies were included in the final review. Studies were primarily excluded because they were published in a language other than English, they did not provide a comparison group, or it was unclear if those who delivered the intervention (e.g., staff, research assistant, principle investigator) were health care providers (Fig 1).

### Focus of this rapid scoping review

Given the primary focus of this rapid scoping review (Question 1) was to identify primary research that evaluated provider care delivered in LTCHs, the remainder of the results focuses on healthcare service delivery interventions (description provided in S2 File under Synthesis) [29]. Briefly, health service delivery interventions included those which introduced a new member to the LTCH to provide an additional service (e.g., general practitioner) or an intervention (e.g., physiotherapist providing an exercise program). Studies identified as evaluating implementation strategies (Question 2; n = 274) can be found the full report posted on (https://osf.io/bpxk4/).

### Characteristics of included studies

Ninety-two studies were mapped to healthcare service delivery interventions. The majority of the studies were RCT/non-RCTs (n = 66), with 15 comparative cohort studies, and 11 controlled before-after studies. Among all studies, a total 18 countries were represented (Table 3). S6 File under Section 1 presents studies in alphabetical order by each first author’s last name and in which tables they can be found based on the mapping exercise.

**Table 3:** Study characteristics.

Category	Details	Healthcare Service Delivery (n = 92)
*Study design*	RCT/non-RCT	66
	Comparative cohort	15
	ITS/CBA	11
*Year of Publication*	2010	5
	2011	5
	2012	3
	2013	8
	2014	5
	2015	7
	2016	13
	2017	9
	2018	13
	2019	9
	2020	15
*Country of conduct*	Australia	27
	United States of America	12
	Canada	7
	France	7
	United Kingdom^†^	6
	Germany	5
	Japan; New Zealand; Sweden	4 each
	Denmark; The Netherlands	3 each
	Belgium; Norway; Taiwan	2 each
	Italy; Malaysia; Spain; Turkey	1 each
*Studies per category*^⁑^	A. Specialty physicians	23
	B. Primary care	20
	C. Direct patient care	24
	D. Allied health care	45
	E. Prevent admissions	30
	F. Specific condition	9

^†^ England, Northern Ireland, Scotland, Wales.

^⁑^ Studies are not mutually exclusive and could have been mapped to ≥1 category.

### Mapping of healthcare services delivery interventions

Healthcare services delivery interventions were mapped into six categories.

A. Access to specialty physician care/team members (e.g. geriatricians, neurologists)
B. Models to provide primary care (e.g. primary care doctors, nurse practitioners)
C. Models to support direct resident care (e.g. clinical nursing specialties, personal support workers)
D. Models to support access to specialists/other allied health care providers (e.g. pharmacists, physiotherapists, dental hygienists)
E. Models to support access to specialists to avoid acute care hospitalizations (e.g. advice from physician specialists to LTC staff to help avoid hospital)
F. Models of care focused on specific conditions/interventions

Due to the complex nature of the interventions and the different needs of the knowledge users who will use this information, studies have been mapped and are presented in several ways:

1. Studies specific to categories A to F (i.e., only mapped to one category) (Section 2 in S6 File).
2. Studies, which were mapped to two or more categories consisting of A to D are described under multidisciplinary teams (Section 3 in S6 File).
3. Studies mapped to categories A to D, but also E, F or (G and H), can be found in the two points above (Section 2 and 3 in S6 File), with a notation found under the study author name in italics for category E, F, G or H.

Although we did not present studies mapped only to categories G and/or H in this report, the interventions included in categories A to F may have also been mapped to G and/or H, and this has been noted in the relevant tables.

### Main findings—Healthcare services delivery

**Fig 2:**
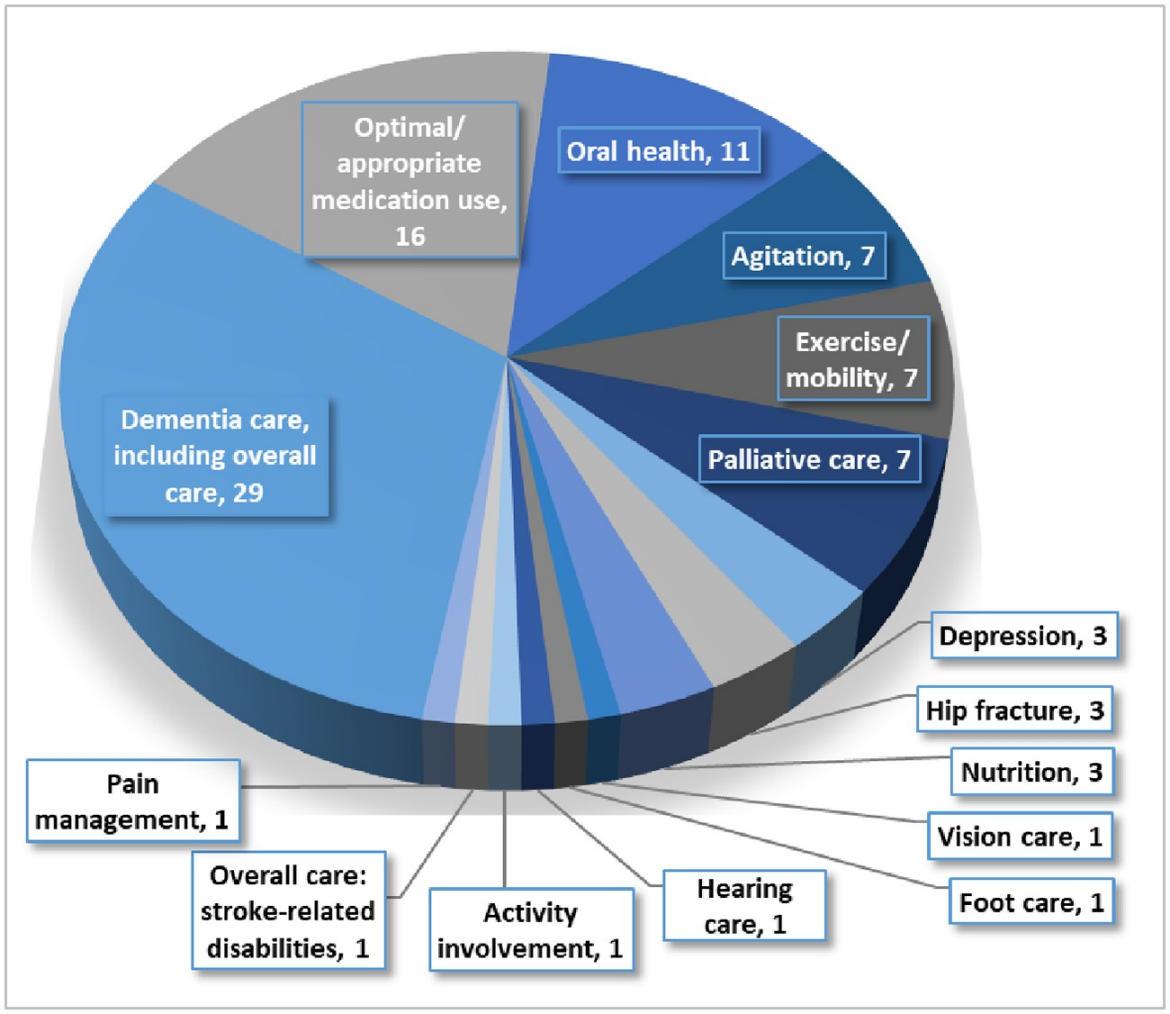
Condition or intervention focus. Fig 2 presents the number of studies addressing each condition/ intervention.

Healthcare service delivery studies were classified into 15 different conditions/ intervention focus (Fig 2).

Related appendices tables provide additional PICOS details, with study authors’ main conclusions.

#### A. Access to specialty physician care

Ten studies evaluated access to specialty physician care (Section 2: Table A in S6 File).

*Synopsis of specialty physician care*. Most studies evaluated the IQUARE intervention (including a geriatrician) in France, which was successful in reducing potentially inappropriate drug prescribing. However, when evaluating the inclusion of a geriatrician for overall care, results were mixed. A very small study evaluating the addition of a licensed osteopathic physician reduced hospitalization and decreased medication usage.

*Overall care*. Three studies evaluated the inclusion of a geriatrician. D’Arcy 2013 (USA) [30] evaluated treatment by a geriatrician (n = 2,477) compared to treatment by other physicians (n = 64,074), which resulted in a reduction in ED use. Gloth 2011 (USA) [31] evaluated a dedicated post-acute care hospitalist by a geriatrician (n = 390) compared to a traditional model with a cadre of community physicians (n = 364), which resulted in an increase in laboratory costs and no improvement in fall rates. Last, Rolland 2020 (France) [32] evaluated the Impact of Systematic Tracking of Dementia Cases on the Rate of Hospitalization in Emergency Care Units (IDEM) which was a team led by a geriatrician (n = 599) compared to usual practice (n = 829), and reported results that did not support a team, which includes a geriatrician to reduce ED transfers.

*Exercise/mobility*. Snider 2012 (USA) [33] reported a pilot study, which evaluated osteopathic manipulative treatment delivered by licensed osteopathic physicians (n = 8) and light touch (n = 6) compared to treatment as usual (n = 7), and resulted in reduced hospitalizations and decreased medication usage. Several other outcomes were evaluated, including activities of daily living (ADL) dependence, cognition, mood, falls, pain, among others.

*Optimal/appropriate medication use*. Six studies in France evaluated the IQUARE (Impact d’une demarche QUAlité sur l’évolution des pratiques et le déclin fonctionnel des Résidents en EHPAD) intervention [34–39], which included cooperative meetings between hospital geriatricians and NH staff, plus audit and feedback (median: n = 1,740, range: 459 to 3,017) compared to audit and feedback only (median: n = 2,080, range: 464 to 3,258). The impact of the intervention mainly resulted in reducing potentially inappropriate drug prescribing. Several other outcomes were evaluated, including contraindications and drug-drug interaction, benzodiazepine use, total number of medications, among others.

#### B. Models for primary care

Eight studies evaluated models for primary care (Section 2: Table B in S6 File).

*Synopsis of primary care*. The addition of nurse practitioners resulted in improved quality of life and improved resident pain, but mixed results around emergency department transfers. The addition of a primary care physician was beneficial with respect to hospitalizations and ED visits. However, these studies had a small number of residents exposed to the intervention (<350 residents). The largest study, in Canada, evaluated same-day physician access for 5617 residents in 52 LTCH compared to physician visits the next day (or later), and resulted in lower hospitalizations and ED visits.

*Overall care*. Three studies evaluated the addition of a NP in collaboration with the primary care physician (median: n = 101, range: 45 to 325) compared to usual/standard care [40, 41], or internal or external control medication review meetings [42] (median: n = 135, range: 99 to 1,056). One study reported an improved quality of life [40], and there were mixed results across the three studies regarding ED transfers. Kobewka 2020 (Canada) [43] evaluated same-day physician access (n = 5,617) compared to physician visits the next day or later (n = 15,007) and resulted in lower hospitalizations and emergency department visits. Weatherall 2019 (Denmark) [44] evaluated assigning a dedicated primary care physician to a home (n = 339) compared to no dedicated primary care physician (n = 26,446), which resulted in a reduction in the probability a resident experienced a preventable hospitalization or a readmission.

*Pain management*. Kaasalainen 2016 (Canada) [45] evaluated a NP-led pain management team (n = 139), which significantly improved resident pain and functional status compared to no NP or pain management team (n = 98). Depression, agitation, clinical practice behaviours and other outcomes also reported.

*Palliative care*. The implementation of the Improving Palliative Care Through Teamwork (IMPACTT) intervention, which included a gero-palliative care nurse practitioner (n = 2,852), was evaluated in two related studies in the USA [46, 47], and did not demonstrate a significant impact on residents’ outcomes compared to no intervention. Several outcomes were evaluated, including death in a hospital, depressive symptoms, perceived palliative care competency, staff satisfaction, among others.

#### C. Models for direct patient care

Ten studies evaluated the addition of direct care providers (e.g., advance practice nurses, certified nursing assistants) for depression or overall care (Section 2: Table C in S6 File).

*Synopsis of direct patient care*. Studies evaluating direct care contributing to reduced depression, increased quality of care and self-efficacy perceptions and health lifestyle behaviours. However, several studies in New Zealand evaluated the RACIP/ARCHUS/ARCHIP intervention, and had mixed results related to transfers to ED, hospitalizations, and length of stay.

*Overall care*. Nine studies evaluated nursing focused interventions, including nurse assistants, gerontology/geriatric nurse specialists (GNS), nurse-led telephone support service, and advanced practice nurses. Most studies compared the intervention to usual care. Among those that reported study size, the median number of participants in the intervention groups was 788 (range: 30 to 1425) and 855 in the comparison groups (range: 30 to 1934). Studies were conducted in Australia, the Netherlands, New Zealand, Turkey, and the USA. Studies reported an increase in quality of care [48, 49], staff skill [50], and self-efficacy perceptions and healthy lifestyle behaviors in older adults [51]. There were mixed results in transfers to the ED [52, 53], acute hospitalizations/ ED admissions [54–56], and hospital stay length [53, 54]. There was no reduction in mortality [54].

*Depression*. Verkaik 2011 (The Netherlands) [57] evaluated the introduction of a nursing guideline on depression, focused on CNAs (n = 62), which significantly reduced depression severity, when compared to usual care (n = 35).

#### D. Allied health care teams

Thirty-seven studies evaluated the addition of allied health care team members (e.g., physiotherapists, dental hygienists), with a focus in several areas (Section 2: Table D in S6 File).

*Synopsis of allied health care*. Interventions evaluating the addition of pharmacists, massage therapist, physiotherapists, occupational therapist, dental hygienists, etc. comprised the largest group of studies (n = 37). The intent or focus of these interventions also covered a wide range of conditions. Overall, the addition of pharmacists, oral health care providers, and exercise programs were beneficial to LTCH residents. Other interventions may have also been beneficial, but due to small sample sizes, firm conclusions could not be made.

*Activity involvement*. Wenborn 2013 (UK) [58] evaluated an occupational therapy intervention around the care home environment and an education program for the staff (n = 104) compared to usual care (n = 106). Overall, there was no evidence to suggest improved QoL or other health outcomes in residents with dementia.

*Dementia care*, *including agitation*. Three studies evaluated interventions for dementia care. Two studies by Moyle 2014 (Australia) [59, 60] evaluated foot massage by trained massage therapists (n = 26) compared to quiet presence (n = 29) in residents with moderate to severe dementia. Agitation increased in both groups, mood was unchanged in both groups, and blood pressure was significantly reduced in both groups. Rodriguez-Mansilla 2013 (Spain) [61] evaluated ear acupuncture (n = 40) or massage therapy (n = 40) compared to no experimental treatment (n = 40) and reported improvement in behaviour and sleep disturbances, and increases in participation in eating and rehabilitation.

*Depression*. Travers 2017 (Australia) [62] evaluated the addition of a mental health therapist to work individually with residents to identify and implement a tailored plan around pleasant events (n = 10) compared to a facility volunteer walking and talking with each resident (one-on-one) (n = 8). Although it increased the number of pleasant events residents participated in, it did not significantly improve depression and QoL, which may be due to the small sample size.

*Exercise/mobility*. Five studies (six publications) evaluated exercise programs, which included aerobic exercise, resistance training, and balance exercises (median: n = 23, range: 20 to 113). Comparisons differed between studies, including chair-based activities (e.g., watching films, reading, conversation), a one-time health education talk, or usual care (median: n = 28, range: 20 to 108). Studies were conducted in Australia, Malaysia, Norway, and Sweden. Overall, results were positive, reporting that physical exercise appears to manage or delay physical decline [18], decreases fall rates [63], increases life satisfaction [64] and perceived positive effects [65], improves balance and strength and reduces apathy and agitation [66, 67].

*Foot care*. Wylie 2017 (Scotland) [68] provided core podiatry support (i.e., routine nail and callus maintenance), in addition to foot orthoses, footwear assessment and provision, and a course of foot and ankle exercises (n = 23) compared to core podiatry only (n = 20). The authors concluded that the intervention was feasible to conduct, but that the effectiveness could not be determined, as it was a pilot study and included 43 residents. Several outcomes were evaluated, including falls, mobility, and activities of daily living, among others.

*Hearing care*. Hopper 2016 (Canada) [69] evaluated hearing ability measured by an audiologist (n = 25) compared to hearing ability measured by LTC staff (n = 25). Health care staff completing the assessments were able to recognize hearing loss.

*Hip fracture rehabilitation*. Beaupre 2020 (Canada) [70] evaluated outreach rehabilitation by a physiotherapist after hospital discharge due to hip fracture (n = 46) compared to usual post-fracture care (n = 31). The authors concluded that an outreach program resulted in a modest, but sustained mobility benefit. Outcomes included quality-adjusted life years (EQ-5D), outpatient visits, physician claim, and inpatient readmissions.

*Nutrition*. Three studies evaluated the addition of team members to provide additional nutritional care (e.g., nutritional support, dietician) (median: n = 94, range: 9 to 125). Comparisons included nutrition coordinator education, and education outreach visit strategy, and usual care (median: n = 78, range: 22 to 249). Studies were conducted in Denmark, Sweden, and Taiwan. Results were mixed, with two studies suggesting the use of nutrition support could be beneficial [71, 72] and one study reporting no difference in nutrition status or physical function in the residents [73].

*Optimal/appropriate medication use*. Seven studies evaluated medication review and/or the addition of a pharmacist to LTCH. Interventions included an in-depth medication review by pharmacy students (n = 22) [74], review of residents’ medications by a clinical pharmacist (n = 90) [75], pharmacist visit once per week (n = 32) [76], the implementation of a residential pharmacist position (n = 74) [77], a part-time pharmacist employed (n = 58) [78], the Fleetwood Northern Ireland model of pharmaceutical care which included a monthly visit by a pharmacist (n = 173) [79], and the addition of a clinical pharmacist applied simplification guide (n = 99) [80]. Comparisons were typically usual or standard care (median: n = 43, range: 23 to 897). Studies were conducted in Australia, Canada, Japan, and Northern Ireland. These studies support the addition of a pharmacist or pharmacist medication review, as it can reduce the number of unnecessary and potential harmful medications taken by residents [74–79] and improve medication administration practices [78, 80]. Several other outcomes were evaluated, including falls, sleep status, adverse events/reactions, ED presentation rates, quality of life, hospitalizations, and mortality.

*Oral health*. Eleven studies evaluated the addition of a dental nurse, dental hygienist, dentist, or oral health therapists to provide examinations, brushing, cleaning and denture care to residents, in addition to in-house training/education for staff (median: n = 31, range: 17 to 144). Studies were conducted in Australia, Germany, Japan, Sweden, and the USA. Comparison groups were largely usual care or no additional intervention (median: n = 25, range: 17 to 141). Overall, the addition of professional care improved oral health/hygiene and reduced caries [81–90], although some studies did not find meaningful differences for clinical or microbiological outcomes [91].

*Overall care*: *Stroke-related disabilities*. Sackley 2016 (UK) [92] evaluated an occupation therapist (OT) intervention for residents with a history of stroke or transient ischaemic attack (n = 568) compared to usual care (n = 474). Overall, there was no evidence to suggest benefit from the intervention. Outcomes included the European Quality of Life-5 Dimensions, activities of daily living, functional mobility, mood, and adverse events.

*Vision care*. Man 2020 (Australia) [93] evaluated an ocular care model from a trained optometrist (n = 95) compared to usual care (n = 83) among visually impaired residents. The model was effective in improving clinical visual outcomes, subjective quality vision, and emotional well-being of residents. Mobility, number of falls, and number of injurious falls also evaluated.

#### E. Models for preventing acute care hospital admission or readmission

Although 30 studies aimed to prevent or reduce acute care hospital admissions or readmissions, 29 have been categorized in A, B, C, or D (n = 14) or are presented below in models for multidisciplinary healthcare service (n = 15) (Section 2: Table E in S6 File).

*Overall care*. Kane 2017 (USA) [94] evaluated the Interventions to Reduce Acute Care Transfers (INTERACT) to identify and evaluate acute changes in NH resident condition (n = 9,050) compared to a combination of patients receiving usual care with no contact and those receiving additional attention (n = 14,428). The intervention had no effect on hospitalization or ED visit rates.

#### F. Models of care focused on specific conditions/interventions

Three studies were interventions best represented by models of care focused on specific conditions, specifically, dementia care (Section 2: Table F in S6 File).

*Synopsis of models of care focused on specific conditions/ interventions*. All studies were related to participants with dementia care. Overall, the interventions were beneficial to staff, reducing aggressive behaviours and increasing self-image. However, there were mixed results in QoL of residents.

*Dementia care*, *including agitation*. Three interventions were evaluated including a dementia outreach service (DEMOS) [95] (n = NR), a movement-oriented restorative care (MRC) intervention [96] (n = 37), and a face-to-face didactic education intervention for staff (n = 51) and family members (n = 37) of residents with dementia [97]. Studies were conducted in Australia and the Netherlands. Overall, interventions were effective in improving staff awareness on various symptoms of dementia, positive attitudes of staff, and reducing aggressive and difficult behaviours [95, 97]. There was also an increase in self-image [96]. However, there were mixed results related to improvements in QoL of residents [96, 97].

#### A. and B. Multidisciplinary healthcare service delivery

Three studies evaluated combined care from specialty primary care and a primary care provider (Section 3: Table A&B in S6 File).

*Synopsis of multidisciplinary healthcare service delivery*: *specialist and primary care*. The addition of a geriatrician and nurse practitioner reduced hospital readmission, and palliative care consults reduced end-of-life acute care.

*Overall care*. Cordato 2018 (Australia) [98] evaluated the addition of a geriatrician and nurse practitioner (n = 22) compared to usual post-discharge care (n = 21). The implementation of this intervention resulted in a cost-effective reduction in hospital readmissions and utilization of other medical services.

*Palliative care*. Two studies in the USA evaluated providing palliative care consults before death (n = 477 and 203) compared to the residents who did not received palliative care consults (n = 1,174 and 429), which resulted in reduced end-of-life acute care [99, 100].

#### A. and C. Multidisciplinary healthcare service delivery

Three studies evaluated the addition of specialty physician care and direct resident care (Section 3: Table A&C in S6 File).

*Synopsis of multidisciplinary healthcare service delivery*: *specialist and direct patient care*. A complex guideline-based intervention reduced agitation and disruption behaviour in residents with dementia. The Residential Care Intervention Program in the Elderly (RECIPE) program did not reduce readmissions, however the post-RECIPE study reduced acute hospital utilization rates.

*Overall care*. Two studies in Australia evaluated the RECIPE program, which introduced geriatricians and aged care nurse specialists to the home. The RECIPE program (n = 57) in the initial study by Harvey 2014 [101] did not reduce readmissions compared to usual care (n = 59), however the post-RECIPE study by Hutchinson 2015 [102] reported that post-RECIPE enrollment (n = 1327) may have had a significant impact on reducing acute hospital utilization rates compared to pre-RECIPE enrollment (2 years prior to enrollment).

*Dementia care*, *including agitation*. Rapp 2013 (Germany) [103] introduced a complex guideline-based intervention, which included the training of nursing home staff, the implementation of structured clinical assessments, the implementation of non-pharmacological interventions, and the optimization of pharmacological interventions (n = 163) and compared it to treatment as usual (n = 141). The authors reported a reduction in agitation and disruption behaviour in residents with dementia. Other outcomes reported are around medication prescriptions (e.g., neuroleptics, antidepressants).

#### A. and D. Multidisciplinary healthcare service delivery

Six studies evaluated the addition of specialty physician care in combination with an allied health member (Section 3: Table A&D in S6 File).

*Synopsis of multidisciplinary healthcare service delivery*: *specialist and allied health care provider*. The addition of a specialist and applied health care provider showed improvement for all conditions, with varying degrees of success, as reported in the main conclusions of the study authors.

*Overall care*. De Luca 2016 (Italy) [104] evaluated the monitoring of vital signs and weekly tele-consultation with either a neurologist or a psychologist (n = 32) compared to standard care (n = 27). The authors concluded that telemedicine can be considered an important tool in improving health and QoL, and reducing hospitalizations.

*Depression*. McSweeney 2012 (Australia) [105] evaluated a specialist mental health consultation for residents with depression (n = 21) compared to no advice regarding the management of depression (n = 23), and reported improving outcomes for depressed residents.

*Hip fracture rehabilitation*. Two studies in Australia [106, 107] (intervention given to the same residents in both studies) reported on a geriatric rehabilitation program for residents who were recovering from hip fracture surgery (n = 121) compared to usual care (n = 119). The intervention showed improved mobility, nutritional status and survival.

*Optimal/appropriate medication use*. Two studies combined a specialist physician and a pharmacist to evaluate medication use. Doernberg 2015 (USA) [108] reported that the intervention (n = 104) had a decrease in antibiotic utilization when compared to the pre-intervention phase (n = 292). Verrue 2012 (Belgium) [109] reported that the intervention (n = 69) modestly improved the appropriateness of prescribing compared to usual care (n = 79). However, both studies reported that the intervention was not used to its full potential.

*Overall care*. De Luca 2016 (Italy) [104] evaluated the use of an electronic box in combination with weekly tele-consultation with either a neurologist or a psychologist (n = 32) compared to standard care (n = 27). The authors concluded that telemedicine can be considered an important tool in improving health and QoL, and reducing hospitalizations.

#### A. and C. and D. Multidisciplinary healthcare service delivery

One study evaluated a multidisciplinary team which included members from several care provider groups (Section 3: Table A&C&D in S6 File).

*Overall care*. Wu 2010 (Taiwan) [110] introduced an interdisciplinary team, composed of a geriatrician, nurses, physical therapists, dieticians and social workers, to actively participate in the daily care of severely disabled residents (n = 42) compared to usual nursing/ personal care with some professional care (n = 32). The authors reported that the clinical effectiveness of this team was minimal.

#### B. and C. Multidisciplinary healthcare service delivery

Nine studies evaluated the combination of primary care and direct resident care (Section 3: Table B&C in S6 File).

*Synopsis of multidisciplinary healthcare service delivery*: *primary care and direct patient care*. Interventions for overall care were beneficial in reducing admissions to emergency or hospital, and length of stay, but not mortality. Needs Rounds were also beneficial for reducing admissions, length of stay, and quality of death and dying.

*Overall care*. Six studies evaluated a variety of combinations of primary care and direct patient care support. Interventions varied, including off-hour physician coverage by a telemedicine service staffed by a medical secretary, RN, NP and physician [111], a clinical manager appointed to support the primary care physician [112], increased collaborative working and establishment of partnerships between health and care providers [113, 114], and a follow-up visit from a geriatric team after discharge [115, 116]. Comparators also varied, including usual care, and external primary care physicians. Three studies provided study size (median: n = 568, range: 318 to 648 participants). Studies were conducted in Australia, Denmark, the UK, and the USA. Briefly, intervention groups were mostly beneficial: telemedicine and in-house primary care physicians reduced admission to hospitals, the vanguard model reduced secondary use resource utilization/ emergency admissions, a follow-up visit from a geriatric team (i.e., doctor and nurse) after discharge from the hospital reduced readmissions and length of stay [115], but did not impact mortality [116].

*Palliative care*. Three studies in Australia evaluated ‘Needs Rounds’ run by specialist palliative care staff (NPs and clinical nurse consultant) to improve communication and relationships between specialist palliative care and the residential facility compared to usual care or care prior to the intervention starting. The intervention reduced admissions to acute care facilities [117, 118], length of stay [118], and improved the quality of death and dying [119].

#### C. and D. Multidisciplinary healthcare service delivery

One study evaluated the addition of a team comprised of a nurse and a psychologist (Section 3: Table C&D in S6 File).

*Optimal/appropriate medication use*. Azermai 2017 (Belgium) [120] evaluated transition to a person-centered support approach (n = 118) compared to no transition towards person-centred care (n = 275). The intervention was successful in reducing in-house psychotropic drug use. Several other drug types were reported, including drugs for the nervous system, cardiovascular, blood, respiratory, etc.

## Discussion

As an extension to the RSC policy briefing on COVID-19 and the future of LTC in Canada [12], we conducted a rapid scoping review to identify studies that evaluated care provider models or services in LTC homes. The identification and mapping of these studies will contribute to the knowledge-base on how healthcare for residents in LTCH may be improved. This rapid scoping review identified 366 studies published since 2010. Using guidance from EPOC [29], two main categories were used to map these included studies; healthcare services delivery and implementation strategies. A preliminary mapping framework, which included eight subcategories, was created and studies were placed into one or more categories. Although some studies were easily mapped to one category (e.g., adding a geriatrician to the nursing home), many interventions were complex and included more than one healthcare provider. Due to the wide variety of interventions evaluated, our approach was to further classify these studies based on the condition or focus of the intervention. Overall, different models of direct care for LTCH residents showed mixed results on systems-level outcomes and little evidence on resident-level outcomes; this area should be targeted for a future systematic review and additional primary research. Access to direct primary care by primary care physicians and/or NPs appeared effective across studies and this is also an area that should be targeted for a future systematic review. Similarly, access to specialist physicians including geriatricians appeared helpful across different outcomes and interventions models.

Care aides/PSWs perform approximately 90% of the direct resident care [12], however, few studies evaluated interventions that included or specifically targeted these workers. COVID-19 has highlighted their vulnerability and how essential this care provider group is for LTCH residents [12]. Therefore, its paramount that future research include this important group of care providers.

The main objective of this rapid scoping review was to identify and map the existing research in this area. However, this mapping exercise has highlighted several gaps in the literature, as several healthcare areas and interventions covering specific conditions are not well represented in the literature. For example, vision care, hearing care, foot care activity involvement, overall care specific to stroke-related disabilities, and pain management were only covered by one article each. Each of these areas are important for overall quality of life of any individual, and should be further evaluated by researchers who conduct primary research studies. Future research priorities and questions should be based on the gaps identified in this review and guided by the residents’ needs, as well as those of their essential care partners. This approach should reflect the diversity of the population to ensure strategies will be contextualized to relevant needs including gender, race, and language amongst other factors.

Although outside of the scope of this review, it is important to consider that the standard healthcare team that is employed in LTCHs in different countries may differ. Additionally, there may also be differences between for-profit and not-for-profit LTCHs within a country. For this reason, the country of conduct and profit status of the LTCHs included in the studies were extracted. This may provide the reader with additional information to contextualize the information and determine its generalizability. A systematic review may conduct a subgroup analysis on these two variables (i.e., country of conduct, profit status).

Methodologically, the standard reporting across primary studies in this realm requires improvement. For example, in this rapid scoping review, studies were excluded when it was not clear who was involved in the intervention (i.e., if it was a healthcare provider). Among the healthcare service delivery studies, 57.8% (54/92) did not report the profit status of the LTCH. Although this area has not been fully explored, there could be important differences in outcomes based on this criterion alone. This is an area of interest, particularly in the Canadian context, where the balance between for profit vs. non-profit LTCHs is shifting across many Canadian provinces, which has resulted in an increase in the number of chain LTCHs and in the number of beds per home [121]. In Ontario 58% of LTCH are privately owned, 24% are non-profit and 16% are publicly owned [122]. In the current COVID-19 pandemic, deaths from COVID-19 in Ontario were higher in for profit homes compared to non-profit and publicly owned homes, 82.5% vs 17.5%, respectively [123].

There is a wide variety of outcomes reported in these studies, which are largely dependent on the intervention that was delivered and its focus/objective. The number and similarity of outcomes would impact meta-analyses in a systematic review and how the rating of the certainty of the evidence [e.g., Grading of Recommendations Assessment, Development and Evaluation (GRADE)] would be performed. A search of the Core Outcome Measures in Effectiveness Trials (COMET) (https://www.comet-initiative.org/) was performed to determine if there are any existing core sets in this area, and resulted in no relevant research. However, the Worldwide Elements to Harmonize Research in LTC Living Environments (WE-THRIVE) initiative has developed a consortium of researchers across 21 countries to identify measurement domains that are internationally relevant and to provide a set of data elements to measure concepts that can be used across studies for data sharing and comparisons [124, 125].

### Limitations of the rapid scoping review

One broad search was developed to capture the wide range of models of care, and therefore may have missed capturing studies if a more specialized search had been done across key conditions or key provider groups. As this was a rapid scoping review, we employed several abbreviations or omission of the methods, for feasibility, which may have missed some relevant studies. For example, in the study identification stage of the review, we included only studies published since 2010 and did not do any supplemental searching (e.g., no grey literature searching, no scanning of the bibliographies of the included studies).

We did not include population-based cohort studies that lacked a comparator group and therefore, we may have missed studies which may have provided further contextual information related to the provision of care within a LTCH. For example, we know of one study that addressed whether residents retained their family physician after LTC entry [126]. This study did involve a comparator group, it did highlight that few residents retained their family physician post-admission to LTC and therefore is a potential breakdown point in terms of continuity of care.

Several studies (n = 56) were mapped to implementation interventions (category G and H) as it was unclear who delivered the intervention. These studies would require additional information from the study author to confirm if the provider team member(s) were a newly accessed service for the LTCH (and therefore would be categorized as healthcare services delivery) or were part of an existing team of care providers. As some countries (e.g., The Netherlands) or jurisdictions may include primary care physician and allied healthcare members as part of their standard care team or provide access to such services as part of the broader healthcare system, no assumptions were made. Additionally, several studies were excluded as it was not clear who delivered the interventions. In some cases, authors simply stated the researcher staff or principal investigator were involved, but we were unable to confirm their credentials or training. In a systematic review, authors of these studies would typically be contacted for additional information, but due to the rapid nature of this scoping review, these studies were excluded.

A total of 366 studies were included in the larger report, and we aimed to be as consistent as possible, but there is a chance that some studies that may have involved different types of intervention focus (e.g., dementia care) or several health care providers may not have been consistently categorized. We felt it was important to classify these studies to provide a framework to potentially identify future research priorities. However, in an effort to minimize inconsistency, two reviewers mapped all studies together through discussions.

## Conclusions

A wide variety of healthcare service delivery and implementation strategy interventions have been evaluated in the published literature in the last decade. Some areas are well represented in the current research, including dementia care, oral care, exercise/mobility, overall resident care, and optimal/appropriate medication use. These areas may present opportunities for additional formal systematic reviews and syntheses. However, other areas of provider care are not well researched (e.g., hearing care, vision care, foot care) yet may have the potential to improve a LTCH resident’s overall quality of life (e.g., promote balance and to prevent falls, subsequent acute care hospitalizations, and the downstream effects of hospitalizations).

## Supporting information

S1 File This is the completed PRISMA-ScR checklist. (DOCX)

S2 File This is the complete description of the methods. (DOCX)

S3 File This is the PRESS Guideline 2015—Search submission & peer review assessment. (DOCX)

S4 File This is the final search strategy. (DOCX)

S5 File This is the data collection forms. This includes the extraction forms details for the mapping and charting extractions.
(DOCX)

S6 File This is the included studies mapping and details. (DOCX)

## Abbreviations

ACP: Advanced care planning
ADL: Activities of daily living
CBA: Controlled before after
CNA: Certified Nursing Assistant
COPD: Chronic Obstructive Pulmonary Disease
ED/ ER: Emergency department/ Emergency room
EQ-5D: European Quality of Life-5 Dimensions
GNS: Gerontology nurse specialist
GP: General physician/practitioner
ITS: Interrupted time series
LoS: Length of stay
LPN: Licensed practical nurse
LTCH: Long-term care homes
MMSE: Mini Mental State Examination
NH: Nursing home
NP: Nurse practitioner
NR: Not reported
OT: Occupational therapist
PA: Physician Assistant
PCC: Person centered care
PCP: Primary care physician
PICOS: Population, Intervention/exposure, Comparator, Outcomes, Study design
PRISMA: Preferred Reporting Items for Systematic Reviews and Meta-Analyses
PSW: Personal support worker
PT: Physiotherapist
PWD: Persons with dementia
QoC: Quality of care
QoL: Quality of life
QoL-AD: Quality of life in Alzheimer’s disease
QUALID: Quality of life in late-stage dementia
RACF: Residential Aged Care Facility
RCT: Randomized controlled trial
RN: Registered Nurse
ScR: Scoping review
SF: Short Form
