# Supplementary material for: Models of provider care in long-term care: A rapid scoping review
Source: PLoS One. 2021 Jul 16;16(7):e0254527. doi: 10.1371/journal.pone.0254527 (PMC8284811; doi:10.1371/journal.pone.0254527)
Supplement: S2 File — (DOCX) [file pone.0254527.s002.docx]

# S2 File. Complete description of the methods

## Literature Search

An information specialist (BS) designed the search using controlled vocabulary (e.g. MeSH) and title/abstract keywords. The search strategy combined concepts for long-term care, terms for various care providers, and certain vocabulary related to specific study designs of interest. Search sources included MEDLINE, Embase, CENTRAL, PsycINFO, and CINAHL. Results were limited to references published since 2010. No language limits were applied. The strategy was peer reviewed by a second Information Specialist (KC) using the Peer Review of Electronic Search Strategies (PRESS) Checklist **(S3 File)** (1). PRESS was returned with suggested minor revisions on July 8, 2020, and an updated search was performed on July 9, 2020 **(S4 File).**

## Research question & PICOS Framework

Eligibility criteria for the scoping review were designed according to the PICOS (population, intervention/exposure, comparator, outcomes, study design) framework; additional considerations related to the timeframe, and language of the publication were also established in the process of study selection. Criteria were developed to capture studies related to both research questions using a single search strategy. We included studies that met the following criteria:

The focus of this rapid scoping review was centered around the care provider perspective (i.e., providing the necessary staff levels, mix of staff, and interventions to the facility, staff, and residents). As such, we developed three primary categories of LTC staff including those that provide direct medical care; nursing care; and allied health care team members.

1. What type/level of **medical care** should be provided and by whom? [e.g., by the primary care physician (PCP) (either the residents’ own PCP, or one PCP assigned in the LTCH for all); should it involve a nurse practitioner (NP) or a physician’s assistant (PA)?]
2. What **type/level of direct patient care** should be provided and by whom? [e.g., by regulated nurses (registered or licensed practical nurses)?]
3. What **type/level of allied health care team support** should be involved/available?

Some studies evaluated these groups separately, while other studies evaluated team-based studies from a multidisciplinary approach. This rapid scoping review captured both.

## Study Selection Criteria

**Population/participants:** Residents of LTCH* (e.g., nursing homes) with any condition (e.g., frailty, dementia). Palliative care was limited to within LTCH; hospice settings, residential homes, and skilled nursing facilities were excluded.

*LTCH, as defined by Health Canada (2), provide living accommodation for people who require on-site delivery of 24-hour, 7 days a week supervised care, including professional health services, personal care and services such as meals, laundry and housekeeping. In other countries, these homes may have other names (e.g., care homes, residential aged care facilities [RACFs]), but offer similar levels of medical care (e.g., physician, nurse) and other services (e.g., meals).

**Interventions/exposure:** Models of provider care, or interventions delivered to facility, staff, and residents in LTC homes. Resident exposure to staffing levels/mix of staffing was of most interest. This included studies evaluating different approaches/ arrangements of staffing (e.g. team-based approach; access to primary care physicians; nurse practitioners or physician assistants; access to allied health workers; involvement of personal support workers; and possibly other approaches). Studies evaluating access to or direct services provided by relevant care providers to LTC residents were also included. Provision of care could be delivered by any of the following (separately or in combination):

- - - - 1. **Medical care** provided by physicians (e.g., primary care physicians; or geriatric medicine specialists), physician assistant; or nurse practitioner. Both physician assistants and nurse practitioners can work autonomously within their scope of practice within primary care (3,4). This also included, for example, access to specialist physicians and palliative physicians.
        2. **Direct patient care** provided by regulated nurses (e.g., registered or licensed practical nurses), personal support workers, and nursing aides.
        3. **Allied health team care** provided by: physical therapists, occupational therapists, speech/ language therapists, recreation therapists, dieticians, podiatry/chiropody, dental, vision care, hearing care, pharmacists, psychologists, and social workers along with those working as aides alongside these positions. Care also included spiritual care, palliative care, advanced care planning, psychosocial/mental health services, cognitive training and those services specific to dementia care.

Interventions delivering art, music, pet, robot or virtual reality interventions were not considered part of this scoping review. Further, studies focused on continuing medical education (CME) or training/education as part of professional development requirements were excluded. We also excluded indirect care, for example, cleaning, laundry, and food preparation.

**Comparator(s)/Control(s):** Studies involving parallel comparisons between different models of provider care as well as studies wherein different models were compared over time (e.g. interrupted time series/controlled before-after studies involving different models at the same LTC home) were included. Comparators of interest were limited to those conducted within or across LTCH.

**Outcomes:**

- **Primary outcomes:** Quality of life (QoL); Quality of care (QoC) [i.e., anything related to quality of care (e.g., urinary tract infection, pressure ulcers, venous thromboembolism (VTE), pain control, use of antipsychotics)]; and health outcomes (e.g., mortality; chronic disease management indicators; appropriateness of prescribing and number of medications; unplanned transfers to hospital, ER admissions/hospitalizations; goal concordant care) were included.
- **Secondary outcomes:** We were also interested in healthcare worker (HCW) stress, burnout or quality of work-life if reported along with a relevant primary outcome. Studies that for example, specifically evaluated interventions to mitigate these HCW outcomes, were not included.

**Study Designs:** Randomized controlled trials (RCTs), non-randomized controlled trials, and quasi-experimental study designs (e.g., controlled before/after studies [CBA]; interrupted time series [ITS]) were of primary interest. We also identified comparative cohort studies for inclusion. Cross-sectional studies, case-control studies, case reports/studies and qualitative literature were excluded for this work.

No geographic restrictions were used. The literature search looked for evidence published between the years 2010-2020. Only English language publications were included in the final scoping review.

## Study Selection Process

Citations from the literature search were collated and de-duplicated in Reference Manager (5). The remaining unique results were then uploaded to DistillerSR Software® (6). Screening was performed in two stages. Citations included at the title and abstract stage were further reviewed at full-text. Pilot testing of screening questions for both levels was completed prior to implementation to calibrate responses. All titles and abstracts and full-text articles were screened by independent reviewers in duplicate (CH, MH, CG, CB, LE, DR) for eligibility. Disagreements during title and abstract and full-text screening were resolved through consensus.

We also used DistillerSR’s® artificial intelligence active-machine learning feature to implement prioritized screening during review of titles/abstracts. This meant that, following a training set of 152 citations, the remaining titles/abstracts were presented to reviewers in an order of perceived highest relevance to lowest relevance (based on a classification algorithm). This helped expedite identification of potentially relevant citations to be screened at full text. Two reviewers screened citations in order of likelihood of inclusion. Four other reviewers screened in random order. After every 152 citations were screened (by two independent reviewers), the remaining unscreened citations were re-ordered based on the new score. Once an estimated recall of 95% of included studies was achieved (828/872), the AI reviewer was assigned to exclude the remaining citations (n=3446). At the time this was run, the highest prediction score that a citation was relevant was 0.1774. A human reviewer screened all of the citations excluded by the AI reviewer, and any conflicts were resolved between two human reviewers. Nine of these records were included by human reviewers to be further reviewed at full text, seven because there was no abstract, and two because it was unclear if the intervention took place in a LTCH. Of these nine, all were excluded when evaluated at full-text.

## Data Collection

We collected data in two phases, mapping and then data extraction.

*Mapping*: Once a study was deemed to be eligible at full text, studies were mapped using a standardized data charting form either evaluating a healthcare service delivery or evaluating an implementation strategy. At this level, we recorded the country where the study took place; a brief description of the model, service or intervention; type of care provided/intervention delivered including a description of the care providers involved; and whether the study was an RCT/non-RCT (including a controlled before-after study), a CBA or ITS, or a comparative cohort. This mapping was conducted by one person (CG and CH) following a pilot exercise of five relevant studies performed in DistillerSR. After initial feedback from a clinical expert, all studies were revisited by two reviewers (CH and CG) to confirm or change the mapping. At this stage, studies were further classified into additional categories (further described in the data charting and visualization section below).

*Data charting*: Studies that were deemed to evaluate a healthcare service delivery, were then directed to another level of further data extraction, including details of the residents, intervention, comparison and outcomes (e.g., quality of life, quality of care, health outcomes), and a summary of the main conclusions. Studies that evaluated implementation strategies were directed to further extraction of the number of LTCH, total number of residents, and number of residents per group. A pilot exercise of five relevant studies was performed in DistillerSR to help develop the draft data charting form. Data charting was performed by one reviewer (CH, MH, CB, DR, LE, NA). Approximately 20% of the extractions were verified by a second reviewer (CH or CG). The extent of breadth and depth of the extraction was limited to these variables for feasibility purposes.

## Risk of bias assessment

A risk of bias assessment of the individual studies was not completed for this scoping review, as the objective was to identify and map interventions offered in LTCH, and not to evaluate the risk of bias of these studies (7).

## Synthesis

As there was a diverse set of included studies with regard to interventions evaluated, study designs used and outcomes reported, we used a descriptive approach to synthesize the included studies. Study characteristics (e.g., author, year, country, number of participants) and population demographics/design/endpoint information (e.g., care provider(s), interventions delivered, relevant outcomes, and general conclusions as stated by the authors) are presented in tabular form. Using guidance from Effective Practice and Organisation of Care (EPOC) (8), we organized the data first by two categories:

1. **Healthcare Services Delivery:** where a new member has been introduced to the LTCH to provide an additional service (e.g., general practitioner) or an intervention (e.g., physiotherapist providing an exercise program). This category was further separated into subcategories. Several complex interventions fell into more than one category.
   1. Access to primary care specialists/team members (e.g. geriatricians, neurologists)
   2. Models to provide primary care (e.g. primary care doctors, nurse practitioners)
   3. Models to support direct resident care (e.g. clinical nursing specialties, personal support workers)
   4. Models to support access to specialists/other allied health care providers (e.g. pharmacists, physiotherapists, dental hygienists)
   5. Models to support access to specialists to avoid acute care hospitalizations (e.g. advice from physician specialists to LTC staff to help avoid hospital)
   6. Models of care focused on specific conditions/interventions
2. **Implementation Strategies:** where existing healthcare members of the LTCH were trained/education in different areas that would impact care (e.g., oral health education) or deliver new care strategies to residents (e.g., reminiscence therapy). Implementation strategies were further divided into two subcategories:
3. Strategies to support multidisciplinary care team: included strategies to optimize care by current LTCH team.
4. Strategies targeting specific conditions/risk factors: included quality improvement strategies that are targeted to a particular condition(s) or risk factor that a LTCH resident may have.

Results are presented using tables and visual representations (e.g., bar charts), as appropriate. A descriptive summary is presented to further provide a cursory overview of the results from the tables and charts. Across studies involving similar care providers within a condition or focus of the intervention provided, we have highlighted consistent and/or contradictory conclusions.

Due to the number of studies and vast amount of information to process over a short duration of time, the results are first presented as a high-level summary for research question 1. Subsequently, for research question 2, we have simply organized this information by type of intervention (and provider(s)) by year, study design, and sample size.

# References

1. McGowan J, Sampson M, Salzwedel DM, Cogo E, Foerster V, Lefebvre C. PRESS Peer Review of Electronic Search Strategies: 2015 Guideline Statement. J Clin Epidemiol. 2016 Jul;75:40–6.

2. Health Canada. Long-term facilities-based care [Internet]. 2004 [cited 2020 Aug 21]. Available from: https://www.canada.ca/en/health-canada/services/home-continuing-care/long-term-facilities-based-care.html

3. Nurse Practitioners [Internet]. College of Nurses of Ontario. 2020 [cited 2020 Oct 13]. Available from: https://www.cno.org/en/learn-about-standards-guidelines/educational-tools/nurse-practitioners/

4. What is a Physician Assistant? [Internet]. Canadian PA. 2020 [cited 2020 Oct 13]. Available from: https://canadianpa.ca/whatisapa/

5. Thomson Reuters. Reference Manager 12, 2011.

6. Evidence Partners. DistillerSR. [Internet]. 2011.

7. Peters, MDJ, Godfrey, C, McInerney, P, Munn, Z, Tricco, A, Khalil, H. Chapter 11: Scoping Reviews. In: Aromataris E, Munn Z (Editors). JBI Manual for Evidence Synthesis. [Internet]. The Joanna Briggs Institute; 2020. Available from: https://wiki.jbi.global/display/MANUAL/Chapter+11%3A+Scoping+reviews

8. Effective Practice and Organisation of Care (EPOC). The EPOC taxonomy of health systems interventions. EPOC Resources for review authors [Internet]. Oslo: Norwegian Knowledge Centre for the Health Services; 2016 [cited 2020 Sep 28]. Available from: https://epoc.cochrane.org/sites/epoc.cochrane.org/files/public/uploads/taxonomy/epoc_taxonomy_guidance.pdf
