# Supplementary material for: Models of provider care in long-term care: A rapid scoping review
Source: PLoS One. 2021 Jul 16;16(7):e0254527. doi: 10.1371/journal.pone.0254527 (PMC8284811; doi:10.1371/journal.pone.0254527)
Supplement: S3 File — (DOCX) [file pone.0254527.s003.docx]

# S3 File. PRESS Guideline 2015— Search Submission & Peer Review Assessment

Reference: McGowan J, Sampson M, Salzwedel DM, Cogo E, Foerster V, Lefebvre C. PRESS Peer Review of Electronic Search Strategies: 2015 guideline statement. *J Clin Epidemiol* 2016;75:40-6. Available: <http://www.jclinepi.com/article/S0895-4356(16)00058-5/pdf>.

**Search submission: This section to be filled in by the searcher**

Searcher: Becky Skidmore Email: [bskidmore@rogers.com](mailto:bskidmore@rogers.com)

Date submitted: 8 Jul 2020 Date requested by: 9 Jul 2020 AM

| 1. **Systematic Review Title** |  |
| --- | --- |

| A rapid review to determine effective models of (provider) care in long-term care nursing homes. |
| --- |
|  |

| X | My PRIMARY (core) database strategy — First time submitting a strategy for search question and database |
| --- | --- |
|  | My PRIMARY (core) strategy — Follow-up review NOT the first time submitting a strategy for search question and database. If this is a response to peer review, itemize the changes made to the review suggestions |
|  | SECONDARY search strategy— First time submitting a strategy for search question and database |
|  | SECONDARY search strategy — NOT the first time submitting a strategy for search question and database. If  this is a response to peer review, itemize the changes made to the review suggestions |

| 1. **Database** (e.g., MEDLINE, CINAHL) *[mandatory]* |
| --- |

MEDLINE

| 1. **Interface** (e.g., Ovid, EbscoHost…) *[mandatory]* |
| --- |

Ovid

| 1. **Research Question** (Describe the purpose of the search)  *[mandatory]* |
| --- |

*What are effective care provider models in long-term care nursing homes to improve the health, quality of life and quality of care outcomes of residents?*

1. What type/level of medical care should be provided and by whom? [e.g., by the primary care physician (PCP) (either the resident’s own PCP, or one PCP assigned in the LTC facility for all); should it involve a nurse practitioner (NP) or a physician’s assistant (PA)?]
2. What type/level of nursing support should be provided and by whom? [e.g., by regulated nurses (registered or licensed practical nurses)?]
3. What type/level of allied health professional support should be involved/available?
4. How should families, friends, or volunteers be optimally involved in caregiving in long-term care?

The focus of the RR is the care provider perspective (i.e., providing the necessary staff levels and mix of staff).

| 1. **PICO Format** Outline the PICOs for your question — i.e., Patient, Intervention, Comparison, Outcome, and Study Design — as applicable |
| --- |

| **P** | Residents of LTC **nursing home** facilities with any condition (e.g., general aging, dementia) |
| --- | --- |
| **I / Exposure** | Models of LTC in nursing homes - Studies evaluating different approaches / arrangements of staffing (e.g. team-based approach; access to primary care physicians; nurse practitioners or physician assistants; access to allied health workers; involvement of personal support workers; and possibly other approaches).  Provision of care could be delivered by staff and resources including physicians (primary care physicians; or geriatric medicine specialists), physician assistant; nurse practitioner; regulated nurses (registered or licensed practical nurses); mental health care, palliative resources, physical therapists, occupational therapists, speech/ language therapists, recreation therapists, dieticians, pharmacists, pastoral care, psychologists, social workers; personal support workers; and various aides in nursing homes. This may also include uninsured services (e.g., podiatry; dental, vision, and hearing care services). |
| **C** | Studies involving parallel comparisons between different models of (provider) care as well as studies wherein different models are compared over time (e.g. interrupted time series/controlled before-after studies involving different models at the same LTCH) will be of interest. |
| **O** | Quality of life; quality of care (i.e., anything related to quality of care (e.g., urinary tract infection, use of foley catheters, pressure ulcers, venous thromboembolism (VTE), pain control, use of antipsychotics); and health outcomes (e.g., mortality; chronic disease management indicators; appropriateness of prescribing and number of medications; unplanned transfers to hospital, ER admissions/hospitalizations; goal concordant care).  We will also identify whether studies cite health care worker stress or quality of work-life; caregiver stress (i.e., involving families); interprofessional communication/collaboration. |
| **S** | Randomized controlled trials, non-randomized controlled trials (e.g., controlled before/after studies; interrupted time series). We will also consider comparative cohort studies if feasible. |

| 1. **Inclusion Criteria** (List criteria such as age groups, study designs, etc., to be included) *[optional]*   **This search strategy is …** |
| --- |

2010-present

| 1. **Exclusion Criteria** (List criteria such as study designs, date limits, etc., to be excluded) **[optional]** |
| --- |

| 1. **Was a search filter applied?** Yes |
| --- |

**If YES, which one(s) (e.g., Cochrane RCT filter, PubMed Clinical Queries filter)? Provide the source if this is a published filter.** *[mandatory if YES to previous question* — *textbox]*

Modified Cochrane HSSS, sensitivity- and precision-maximizing version (2008) for RCTs

| 1. **Notes or comments you feel would be useful for the peer reviewer**  *[optional]* |
| --- |

LTC portion of search has been PRESSed before – very minor changes only (line 9).

Terminal/End-of-life care not of interest – only palliative care services in context of LTC

Not interested in ‘home-like’ models of care and as they are focused more on the built envt. (e.g., Butterfly, Green House, Wellspring and Eden Alternative) – focus instead is on care provider mix.

Have not included terms for family, friends, volunteers deliberately – just terms for caregivers

Also interested in uninsured services but such broad range have just used term “uninsured”

Small amount of redundancy in terms pertaining to physicians

Will add observational studies later, time permitting.

| 1. **Please copy and paste your search strategy here, exactly as run, including the number of hits per line. [mandatory]** |
| --- |

Database: Ovid MEDLINE(R) ALL <1946 to July 07, 2020>

Search Strategy:

--------------------------------------------------------------------------------

1 Long-Term Care/ (25867)

2 ((longterm or long-term) adj3 (care or facility or facilities or healthcare or health care or institution or institutions)).tw,kf. (26383)

3 (LTC or LTCF or LTCFs or RACF or RACFs).tw,kf. (4624)

4 (care home or care homes).tw,kf. (3961)

5 (care facility or care facilities).tw,kf. (22079)

6 (residential adj (care or facility or facilities or healthcare or health care)).tw,kf. (4428)

7 (SNF or SNFs).tw,kf. (4313)

8 Institutionalization/ (5322)

9 (institutionali#ed or institutionali#ation*).tw,kf. (14895)

10 Homes for the Aged/ (13854)

11 home? for the aged.tw,kf. (1479)

12 home? for the elderly.tw,kf. (1452)

13 ((facility or facilities) adj "for the aged").tw,kf. (23)

14 ((facility or facilities) adj "for the elderly").tw,kf. (13)

15 institution? for the aged.tw,kf. (133)

16 institution? for the elderly.tw,kf. (219)

17 ("old age" adj (facility or facilities or home or homes or institution?)).tw,kf. (430)

18 (("aged care" or "aged healthcare" or "aged health care") adj3 (facility or facilities or home or homes or institution?)).tw,kf. (1221)

19 (("elder care" or "elder healthcare" or "elder health care") adj3 (facility or facilities or home or homes or institution?)).tw,kf. (67)

20 (("geriatric care" or "geriatric healthcare" or "geriatric health care") adj3 (facility or facilities or home or homes or institution?)).tw,kf. (116)

21 (("psychogeriatric care" or "psychogeriatric healthcare" or "psychogeriatric health care") adj3 (facility or facilities or home or homes or institution?)).tw,kf. (5)

22 (("gerontologic care" or "gerontologic healthcare" or "gerontologic health care") adj3 (facility or facilities or home or homes or institution?)).tw,kf. (0)

23 (("gerontological care" or "gerontological healthcare" or "gerontological health care") adj3 (facility or facilities or home or homes or institution?)).tw,kf. (1)

24 exp Nursing Homes/ (39136)

25 nursing home?.tw,kf. (30569)

26 (nursing facility or nursing facilities).tw,kf. (3999)

27 or/1-26 [LTC] (126836)

28 Models, Organizational/ (18964)

29 (organi#ational adj2 model?).tw,kf. (1242)

30 (model? adj3 (care or healthcare or health care or home*)).tw,kf. (39984)

31 Patient Care Management/ (4114)

32 Delivery of Health Care/ (90388)

33 exp "Delivery of Health Care, Integrated"/ (12809)

34 ((collaborat* or comprehensive* or connect* or coordinat* or co-ordinat* or integrat* or interconnect* or inter-connect*) adj3 (care or healthcare or health care or team?)).tw,kf. (50926)

35 ((interprofessional* or inter-professional*) adj3 (care or collaborat* or connect* or coordinat* or co-ordinat* or healthcare or health care or integrat* or interconnect* or inter-connect* or network* or team?)).tw,kf. (5549)

36 ((colocat* or co-locat*) adj3 (care or healthcare or health care)).tw,kf. (178)

37 Health Services Accessibility/ (74078)

38 (access* adj3 (care or healthcare or health care or health service?)).tw,kf. (44443)

39 Continuity of Patient Care/ (19060)

40 Patient Care Team/ (65189)

41 (team* adj3 (care or healthcare or health care)).tw,kf. (21083)

42 (team* adj3 (approach* or model?)).tw,kf. (9806)

43 teamwork*.tw,kf. (10118)

44 Health Services for the Aged/ (17673)

45 (health service? adj3 (aged or elder* or geriatric* or gerontolog* or older*)).tw,kf. (1220)

46 Dental Care for Aged/ (2015)

47 ((dental care or dentistry or oral care or oral health or oral healthcare or oral hygiene) adj3 (aged or elder* or geriatric* or gerontolog* or older*)).tw,kf. (1923)

48 Physicians/ (89030)

49 General Practitioners/ (7868)

50 Physicians, Family/ (16360)

51 Physicians, Primary Care/ (3438)

52 ((clinician? or doctor? or physician? or practitioner?) adj3 family*).tw,kf. (25425)

53 ((FP or FPs or GP or GPs) adj5 (physician? or practitioner*)).tw,kf. (12856)

54 ((clinician? or doctor? or physician? or practitioner?) adj3 primary care).tw,kf. (26300)

55 ((clinician? or doctor? or physician? or practitioner?) adj3 primary healthcare).tw,kf. (267)

56 ((clinician? or doctor? or physician? or practitioner?) adj3 primary health care).tw,kf. (880)

57 ((PCP or PCPs) adj5 (primary care or primary healthcare or primary health care)).tw,kf. (3355)

58 general practitioner?.tw,kf. (50352)

59 Family Practice/ (65179)

60 General Practice/ (13296)

61 (family medicine or family practice? or general practice?).tw,kf. (59690)

62 (doctor? or physician?).tw,kf. (495803)

63 Physician Assistants/ (5680)

64 ((clinician? or doctor? or physician? or practitioner?) adj2 (assistant* or extender*)).tw,kf. (4991)

65 feldsher?.tw,kf. (479)

66 Geriatricians/ (119)

67 (geriatrician* or gerontologist*).tw,kf. (2687)

68 Nurses/ (38928)

69 (nurse or nurses).tw,kf. (263718)

70 Nurse Practitioners/ (17626)

71 (nurs* adj2 practitioner?).tw,kf. (13009)

72 (NP adj5 (nurs* or practitioner?)).tw,kf. (925)

73 Nursing, Practical/ (3437)

74 (nurs* adj2 practical).tw,kf. (1850)

75 Nursing Assistants/ (4190)

76 (nurs* adj2 assistant?).tw,kf. (3347)

77 nurse* aide?.tw,kf. (941)

78 nursing auxiliar*.tw,kf. (176)

79 Nursing Staff/ (21101)

80 Allied Health Personnel/ (11725)

81 allied health.tw,kf. (8757)

82 (healthcare assistant? or health care assistant? or healthcare aide? or health care aide? or healthcare worker? or health care worker? or HCW or HCWs).tw,kf. (24363)

83 Licensed Practical Nurses/ (73)

84 (LPN or LPNs).tw,kf. (1506)

85 vocational nurs*.tw,kf. (178)

86 (support worker? or PSW or PSWs).tw,kf. (1245)

87 (care aide? or healthcare aide?).tw,kf. (380)

88 Dentists/ (17975)

89 (dentist or dentists or periodontist? or perio-dontist?).tw,kf. (36626)

90 exp Dental Auxiliaries/ (13175)

91 (dental adj2 auxiliar*).tw,kf. (630)

92 (dental adj2 assistant*).tw,kf. (1508)

93 denturist*.tw,kf. (95)

94 hygienist*.tw,kf. (5086)

95 Nutritionists/ (1216)

96 (nutritionist* or dietician* or dietitian*).tw,kf. (10207)

97 Occupational Therapists/ (302)

98 (occupational adj2 therapist*).tw,kf. (5744)

99 Pharmacists/ (16685)

100 pharmacist*.tw,kf. (33148)

101 Physical Therapists/ (1849)

102 Physical Therapist Assistants/ (13)

103 (physical therapist? or physiotherapist? or physio-therapist?).tw,kf. (14115)

104 (podiatrist? or chiropodist?).tw,kf. (992)

105 ((podiatr* or chiropod* or foot or feet) adj3 (care or healthcare or health care or nurs* or service? or support*)).tw,kf. (2924)

106 psychologist?.tw,kf. (15487)

107 (recreation* adj2 therapist*).tw,kf. (45)

108 Social Workers/ (610)

109 (socialworker* or social worker* or social service? or social support?).tw,kf. (59529)

110 ((speech* or language*) adj2 (pathologist* or therapist*)).tw,kf. (4989)

111 "Personnel Staffing and Scheduling"/ (17045)

112 Personnel Turnover/ (5166)

113 ((personnel or staff*) adj3 (mix* or compos* or compris*)).tw,kf. (1161)

114 (staff* adj2 level?).tw,kf. (3044)

115 (turnover* or turn* over*).tw,kf. (100622)

116 exp Workforce/ (76074)

117 (workforce? or work force?).tw,kf. (27093)

118 manpower*.tw,kf. (7468)

119 staffing.tw,kf. (14052)

120 ((care or healthcare or health care or health service?) adj3 (mix* or compos* or compris*)).tw,kf. (3825)

121 exp Dementia/nu [nursing] (6284)

122 ((dementia or memory) adj3 (care or healthcare or health care or nurs* or service? or support*)).tw,kf. (9090)

123 Medicalization/ (420)

124 medicali*.tw,kf. (2667)

125 Mental Health Services/ (33681)

126 (mental health service? or mental health care or mental healthcare).tw,kf. (30720)

127 (mental hygiene service? or mental hygiene care or mental hygiene care).tw,kf. (106)

128 Palliative Care/ (53940)

129 palliat.tw,kf. (11)

130 Pastoral Care/ (3473)

131 (pastor or pastors or pastoral or minister? or priest? or rabbi? or iman?).tw,kf. (183195)

132 ((eye? or ocular or optical or vision) adj3 (care or healthcare or health care or service?)).tw,kf. (6588)

133 (hearing adj3 (care or healthcare or health care or service?)).tw,kf. (877)

134 Caregivers/ (36491)

135 (caregiver? or care giver? or carer or carers).tw,kf. (79574)

136 uninsured.tw,kf. (8112)

137 Health Care Reform/ (32492)

138 (reform* adj3 (care or healthcare or health care)).tw,kf. (11663)

139 or/28-138 (1905907)

140 27 and 139 [LTC - MODELS OF CARE] (46619)

141 (controlled clinical trial or randomized controlled trial or pragmatic clinical trial or equivalence trial).pt. (598875)

142 "Clinical Trials as Topic"/ (191936)

143 exp "Controlled Clinical Trials as Topic"/ (142805)

144 (randomi#ed or randomi#ation? or randomly or RCT or placebo*).tw,kf. (989212)

145 ((singl* or doubl* or trebl* or tripl*) adj (mask* or blind* or dumm*)).tw,kf. (173234)

146 trial.ti. (221365)

147 or/141-146 (1418704)

148 140 and 147 [LTC - MODELS OF CARE - RCTS] (3546)

149 controlled clinical trial.pt. (93749)

150 Controlled Clinical Trial/ or Controlled Clinical Trials as Topic/ (99222)

151 (control* adj2 trial).tw,kf. (159531)

152 Non-Randomized Controlled Trials as Topic/ (712)

153 (nonrandom* or non-random* or quasi-random* or quasi-experiment*).tw,kf. (56811)

154 (nRCT or non-RCT).tw,kf. (322)

155 Controlled Before-After Studies/ (526)

156 (control* adj3 ("before and after" or "before after")).tw,kf. (4345)

157 Interrupted Time Series Analysis/ (907)

158 time series.tw,kf. (31397)

159 (pre- adj3 post-).tw,kf. (83291)

160 (pretest adj3 posttest).tw,kf. (5582)

161 Historically Controlled Study/ (185)

162 (control* adj2 study).tw,kf. (173637)

163 Control Groups/ (1677)

164 (control* adj2 group?).tw,kf. (504923)

165 trial.ti. (221365)

166 or/149-165 (1108283)

167 140 and 166 [LTC - MODELS OF CARE - nRCTS] (3036)

168 148 or 167 [RCTs, nRCTs] (4605)

169 limit 168 to yr="2010-current" (2612)

170 exp Animals/ not Humans/ (4714911)

171 169 not 170 [ANIMAL-ONLY REMOVED] (2612)

172 (comment or editorial or news or newspaper article).pt. (1429127)

173 (letter not (letter and randomized controlled trial)).pt. (1083153)

174 171 not (172 or 173) [OPINION PIECES REMOVED] (2604)

***************************

**Peer review assessment: this section to be filled in by the reviewer**

|  | Reviewer: Kaitryn Campbell | Email: kcamlolo668@gmail.com | Date completed: 8 Jul 2020 |
| --- | --- | --- | --- |
|  |  |  |  |

Do you wish to be acknowledged? (If yes, the review team will be advised to add an acknowledgement to any publications related to this work). No – unless your organization requires it

The suggested acknowledgement is “We thank Xxxxx Yyyyyy, MLIS, AHIP (xxxxx Health Sciences Library, University of xxxxxx) for peer review of the MEDLINE search strategy.” [please edit to indicate your name, postnomials and institutional affiliation as you would like them presented].

|  | **1. TRANSLATION** |  | | |  |
| --- | --- | --- | --- | --- | --- |
| A -­‐No revisions | X |  |  |  |  |
| B -­‐ Revision(s) suggested |  |  |  |  |  |
| C -­‐ Revision(s) required |  |  |  |  |  |

If “B” or “C,” please provide an explanation or example:

**2. BOOLEAN AND PROXIMITY OPERATORS**

| A -­‐No revisions | X |
| --- | --- |
| B -­‐ Revision(s) suggested |  |
| C -­‐ Revision(s) required |  |

If “B” or “C,” please provide an explanation or example:

**3. SUBJECT HEADINGS**

| A -­‐No revisions |  |
| --- | --- |
| B -­‐ Revision(s) suggested | X |
| C -­‐ Revision(s) required |  |

If “B” or “C,” please provide an explanation or example:

For nursing concept, consider adding: Nurse Clinicians/ OR Advanced Practice Nursing/ OR ((clinical OR clinician? OR consultant? OR (advance? ADJ practic*)) ADJ2 nurs*).tw,kf.

**4. TEXT WORD SEARCHING**

| A -­‐No revisions |  |
| --- | --- |
| B -­‐ Revision(s)suggested | X |
| C -­‐ Revision(s) required |  |

If “B” or “C,” please provide an explanation or example:

Line 43, suggest also considering: team-work*

Line 131, consider adding: spiritual care, healthcare, etc.

**5. SPELLING, SYNTAX, AND LINE NUMBERS**

| A -­‐No revisions | X |
| --- | --- |
| B -­‐ Revision(s)suggested |  |
| C -­‐ Revision(s) required |  |

If “B” or “C,” please provide an explanation or example:

**6. LIMITS AND FILTERS**

| A -­‐No revisions | X |
| --- | --- |
| B -­‐ Revision(s) suggested |  |
| C -­‐ Revision(s) required |  |

If “B” or “C,” please provide an explanation or example:

OVERALL EVALUATION (Note: If one or more “revision required” is noted above, the response below must be “revisions required”.)

| A -­‐No revisions |  |
| --- | --- |
| B -­‐ Revision(s) suggested | X |
| C -­‐ Revision(s) required |  |

Additional comments:

Nicely done, no errors or omissions detected. A couple of suggestions.
