# Supplementary material for: Models of provider care in long-term care: A rapid scoping review
Source: PLoS One. 2021 Jul 16;16(7):e0254527. doi: 10.1371/journal.pone.0254527 (PMC8284811; doi:10.1371/journal.pone.0254527)
Supplement: S4 File — (DOCX) [file pone.0254527.s004.docx]

# S4 File. Final Search Strategy

2020 Jul 9

Ovid Multifile

Database: Embase Classic+Embase <1947 to 2020 July 08>, Ovid MEDLINE(R) ALL <1946 to July 08, 2020>, APA PsycInfo <1806 to June Week 5 2020>, EBM Reviews - Cochrane Central Register of Controlled Trials <June 2020>

Search Strategy:

------------------------------------------------------------

1     Long-Term Care/ (162933)

2     ((longterm or long-term) adj3 (care or facility or facilities or healthcare or health care or institution or institutions)).tw,kf. (71825)

3     (LTC or LTCF or LTCFs or RACF or RACFs).tw,kf. (11856)

4     (care home or care homes).tw,kf. (11786)

5     (care facility or care facilities).tw,kf. (58782)

6     (residential adj (care or facility or facilities or healthcare or health care)).tw,kf. (15671)

7     (SNF or SNFs).tw,kf. (10827)

8     Institutionalization/ (18368)

9     (institutionali#ed or institutionali#ation*).tw,kf. (52292)

10     Homes for the Aged/ (26653)

11     home? for the aged.tw,kf. (4439)

12     home? for the elderly.tw,kf. (4069)

13     ((facility or facilities) adj "for the aged").tw,kf. (70)

14     ((facility or facilities) adj "for the elderly").tw,kf. (52)

15     institution? for the aged.tw,kf. (466)

16     institution? for the elderly.tw,kf. (589)

17     ("old age" adj (facility or facilities or home or homes or institution?)).tw,kf. (1097)

18     (("aged care" or "aged healthcare" or "aged health care") adj3 (facility or facilities or home or homes or institution?)).tw,kf. (3203)

19     (("elder care" or "elder healthcare" or "elder health care") adj3 (facility or facilities or home or homes or institution?)).tw,kf. (181)

20     (("geriatric care" or "geriatric healthcare" or "geriatric health care") adj3 (facility or facilities or home or homes or institution?)).tw,kf. (326)

21     (("psychogeriatric care" or "psychogeriatric healthcare" or "psychogeriatric health care") adj3 (facility or facilities or home or homes or institution?)).tw,kf. (16)

22     (("gerontologic care" or "gerontologic healthcare" or "gerontologic health care") adj3 (facility or facilities or home or homes or institution?)).tw,kf. (0)

23     (("gerontological care" or "gerontological healthcare" or "gerontological health care") adj3 (facility or facilities or home or homes or institution?)).tw,kf. (5)

24     exp Nursing Homes/ (103771)

25     nursing home?.tw,kf. (85593)

26     (nursing facility or nursing facilities).tw,kf. (11474)

27     or/1-26 [LTC] (444185)

28     Models, Organizational/ (64427)

29     (organi#ational adj2 model?).tw,kf. (5015)

30     (model? adj3 (care or healthcare or health care or home*)).tw,kf. (112035)

31     Patient Care Management/ (285337)

32     Delivery of Health Care/ (230543)

33     exp "Delivery of Health Care, Integrated"/ (24368)

34     ((collaborat* or comprehensive* or connect* or coordinat* or co-ordinat* or integrat* or interconnect* or inter-connect*) adj3 (care or healthcare or health care or team?)).tw,kf. (149132)

35     ((interprofessional* or inter-professional*) adj3 (care or collaborat* or connect* or coordinat* or co-ordinat* or healthcare or health care or integrat* or interconnect* or inter-connect* or network* or team?)).tw,kf. (15196)

36     ((colocat* or co-locat*) adj3 (care or healthcare or health care)).tw,kf. (552)

37     Health Services Accessibility/ (122716)

38     (access* adj3 (care or healthcare or health care or health service?)).tw,kf. (117821)

39     Continuity of Patient Care/ (284125)

40     Patient Care Team/ (277697)

41     (team* adj3 (care or healthcare or health care)).tw,kf. (62136)

42     (team* adj3 (approach* or model?)).tw,kf. (29981)

43     (teamwork* or team work*).tw,kf. (39381)

44     Health Services for the Aged/ (54133)

45     (health service? adj3 (aged or elder* or geriatric* or gerontolog* or older*)).tw,kf. (3458)

46     Dental Care for Aged/ (23625)

47     ((dental care or dentistry or oral care or oral health or oral healthcare or oral hygiene) adj3 (aged or elder* or geriatric* or gerontolog* or older*)).tw,kf. (4115)

48     Physicians/ (308844)

49     General Practitioners/ (111384)

50     Physicians, Family/ (114192)

51     Physicians, Primary Care/ (106716)

52     ((clinician? or doctor? or physician? or practitioner?) adj3 family*).tw,kf. (67894)

53     ((FP or FPs or GP or GPs) adj5 (physician? or practitioner*)).tw,kf. (35296)

54     ((clinician? or doctor? or physician? or practitioner?) adj3 primary care).tw,kf. (71620)

55     ((clinician? or doctor? or physician? or practitioner?) adj3 primary healthcare).tw,kf. (674)

56     ((clinician? or doctor? or physician? or practitioner?) adj3 primary health care).tw,kf. (2241)

57     ((PCP or PCPs) adj5 (primary care or primary healthcare or primary health care)).tw,kf. (11236)

58     general practitioner?.tw,kf. (134861)

59     Family Practice/ (149251)

60     General Practice/ (98813)

61     (family medicine or family practice? or general practice?).tw,kf. (144961)

62     (doctor? or physician?).tw,kf. (1354444)

63     Physician Assistants/ (11837)

64     ((clinician? or doctor? or physician? or practitioner?) adj2 (assistant* or extender*)).tw,kf. (13209)

65     feldsher?.tw,kf. (894)

66     Geriatricians/ (2532)

67     (geriatrician* or gerontologist*).tw,kf. (9484)

68     Nurses/ (165341)

69     (nurse or nurses).tw,kf. (673139)

70     Nurse Practitioners/ (38980)

71     (nurs* adj2 practitioner?).tw,kf. (34920)

72     (NP adj5 (nurs* or practitioner?)).tw,kf. (2715)

73     Nursing, Practical/ (3589)

74     (nurs* adj2 practical).tw,kf. (4352)

75     Nursing Assistants/ (9919)

76     (nurs* adj2 assistant?).tw,kf. (9176)

77     nurse* aide?.tw,kf. (2233)

78     nursing auxiliar*.tw,kf. (410)

79     Nursing Staff/ (93441)

80     Nurse Clinicians/ (10302)

81     Advanced Practice Nursing/ (3538)

82     ((clinical or clinician? or consultant? or (advance? adj practic*)) adj2 nurs*).tw,kf. (44697)

83     Allied Health Personnel/ (27606)

84     allied health.tw,kf. (22859)

85     (healthcare assistant? or health care assistant? or healthcare aide? or health care aide? or healthcare worker? or health care worker? or HCW or HCWs).tw,kf. (59895)

86     Licensed Practical Nurses/ (329)

87     (LPN or LPNs).tw,kf. (4146)

88     vocational nurs*.tw,kf. (476)

89     (support worker? or PSW or PSWs).tw,kf. (3971)

90     (care aide? or healthcare aide?).tw,kf. (925)

91     Dentists/ (41830)

92     (dentist or dentists or periodontist? or perio-dontist?).tw,kf. (78520)

93     exp Dental Auxiliaries/ (26538)

94     (dental adj2 auxiliar*).tw,kf. (1302)

95     (dental adj2 assistant*).tw,kf. (3073)

96     denturist*.tw,kf. (192)

97     hygienist*.tw,kf. (11632)

98     Nutritionists/ (13368)

99     (nutritionist* or dietician* or dietitian*).tw,kf. (32683)

100     Occupational Therapists/ (9436)

101     (occupational adj2 therapist*).tw,kf. (21769)

102     Pharmacists/ (94818)

103     pharmacist*.tw,kf. (113576)

104     Physical Therapists/ (22870)

105     Physical Therapist Assistants/ (83)

106     (physical therapist? or physiotherapist? or physio-therapist?).tw,kf. (48717)

107     (podiatrist? or chiropodist?).tw,kf. (2566)

108     ((podiatr* or chiropod* or foot or feet) adj3 (care or healthcare or health care or nurs* or service? or support*)).tw,kf. (8081)

109     psychologist?.tw,kf. (133001)

110     (recreation* adj2 therapist*).tw,kf. (273)

111     Social Workers/ (23708)

112     (socialworker* or social worker* or social service? or social support?).tw,kf. (228260)

113     ((speech* or language*) adj2 (pathologist* or therapist*)).tw,kf. (18441)

114     "Personnel Staffing and Scheduling"/ (71738)

115     Personnel Turnover/ (64976)

116     ((personnel or staff*) adj3 (mix* or compos* or compris*)).tw,kf. (3407)

117     (staff* adj2 level?).tw,kf. (8754)

118     (turnover* or turn* over*).tw,kf. (244594)

119     exp Workforce/ (83769)

120     (workforce? or work force?).tw,kf. (74860)

121     manpower*.tw,kf. (19089)

122     staffing.tw,kf. (38637)

123     ((care or healthcare or health care or health service?) adj3 (mix* or compos* or compris*)).tw,kf. (11235)

124     exp Dementia/nu [nursing] (6288)

125     ((dementia or memory) adj3 (care or healthcare or health care or nurs* or service? or support*)).tw,kf. (30384)

126     Medicalization/ (1213)

127     medicali*.tw,kf. (8571)

128     Mental Health Services/ (123196)

129     (mental health service? or mental health care or mental healthcare).tw,kf. (112159)

130     (mental hygiene service? or mental hygiene care or mental hygiene care).tw,kf. (181)

131     Palliative Care/ (142914)

132     palliat.tw,kf. (57)

133     Pastoral Care/ (3841)

134     (pastor or pastors or pastoral or minister? or priest? or rabbi? or iman?).tw,kf. (435493)

135     (spiritual* adj3 (care or comfort* or support*)).tw,kf. (10744)

136     ((eye? or ocular or optical or vision) adj3 (care or healthcare or health care or service?)).tw,kf. (16146)

137     (hearing adj3 (care or healthcare or health care or service?)).tw,kf. (2477)

138     uninsured.tw,kf. (21200)

139     Health Care Reform/ (209309)

140     (reform* adj3 (care or healthcare or health care)).tw,kf. (27036)

141     or/28-140 (5204177)

142     27 and 141 [LTC - MODELS OF CARE] (134694)

143     (controlled clinical trial or randomized controlled trial or pragmatic clinical trial or equivalence trial).pt. (1184624)

144     "Clinical Trials as Topic"/ (303163)

145     exp "Controlled Clinical Trials as Topic"/ (339954)

146     (randomi#ed or randomi#ation? or randomly or RCT or placebo*).tw,kf. (3587944)

147     ((singl* or doubl* or trebl* or tripl*) adj (mask* or blind* or dumm*)).tw,kf. (718846)

148     trial.ti. (882698)

149     or/143-148 (4515204)

150     142 and 149 [LTC - MODELS OF CARE - RCTS] (12849)

151     controlled clinical trial.pt. (185345)

152     Controlled Clinical Trial/ or Controlled Clinical Trials as Topic/ (573317)

153     (control* adj2 trial).tw,kf. (648701)

154     Non-Randomized Controlled Trials as Topic/ (11590)

155     (nonrandom* or non-random* or quasi-random* or quasi-experiment*).tw,kf. (159742)

156     (nRCT or non-RCT).tw,kf. (924)

157     Controlled Before-After Studies/ (218945)

158     (control* adj3 ("before and after" or "before after")).tw,kf. (744406)

159     Interrupted Time Series Analysis/ (213919)

160     time series.tw,kf. (75776)

161     (pre- adj3 post-).tw,kf. (291760)

162     (pretest adj3 posttest).tw,kf. (22513)

163     Historically Controlled Study/ (229418)

164     (control* adj2 study).tw,kf. (535828)

165     Control Groups/ (125885)

166     (control* adj2 group?).tw,kf. (1546057)

167     trial.ti. (882698)

168     or/151-167 (4252144)

169     142 and 168 [LTC - MODELS OF CARE - nRCTS] (12855)

170     150 or 169 [RCTs, nRCTs] (17561)

171     limit 170 to yr="2010-current" (10871)

172     exp Animals/ not Humans/ (17786270)

173     171 not 172 [ANIMAL-ONLY REMOVED] (7732)

174     (comment or editorial or news or newspaper article).pt. (2089450)

175     (letter not (letter and randomized controlled trial)).pt. (2208236)

176     173 not (174 or 175) [OPINION PIECES REMOVED] (7698)

177     176 use medall [MEDLINE RECORDS] (2345)

178     long term care/ (162933)

179     ((longterm or long-term) adj3 (care or facility or facilities or healthcare or health care or institution or institutions)).tw,kw. (75959)

180     (LTC or LTCF or LTCFs or RACF or RACFs).tw,kw. (11879)

181     (care home or care homes).tw,kw. (11982)

182     (care facility or care facilities).tw,kw. (59777)

183     (residential adj (care or facility or facilities or healthcare or health care)).tw,kw. (15962)

184     (SNF or SNFs).tw,kw. (10889)

185     institutional care/ (5961)

186     institutionalization/ (18368)

187     (institutionali#ed or institutionali#ation*).tw,kw. (52515)

188     home for the aged/ (12855)

189     home? for the aged.tw,kw. (4607)

190     home? for the elderly.tw,kw. (4074)

191     ((facility or facilities) adj "for the aged").tw,kw. (70)

192     ((facility or facilities) adj "for the elderly").tw,kw. (61)

193     institution? for the aged.tw,kw. (466)

194     institution? for the elderly.tw,kw. (590)

195     ("old age" adj (facility or facilities or home or homes or institution?)).tw,kw. (1003)

196     (("aged care" or "aged healthcare" or "aged health care") adj3 (facility or facilities or home or homes or institution?)).tw,kw. (3205)

197     (("elder care" or "elder healthcare" or "elder health care") adj3 (facility or facilities or home or homes or institution?)).tw,kw. (181)

198     (("geriatric care" or "geriatric healthcare" or "geriatric health care") adj3 (facility or facilities or home or homes or institution?)).tw,kw. (344)

199     (("psychogeriatric care" or "psychogeriatric healthcare" or "psychogeriatric health care") adj3 (facility or facilities or home or homes or institution?)).tw,kw. (17)

200     (("gerontologic care" or "gerontologic healthcare" or "gerontologic health care") adj3 (facility or facilities or home or homes or institution?)).tw,kw. (4)

201     (("gerontological care" or "gerontological healthcare" or "gerontological health care") adj3 (facility or facilities or home or homes or institution?)).tw,kw. (5)

202     nursing home/ (71846)

203     nursing home?.tw,kw. (87041)

204     (nursing facility or nursing facilities).tw,kw. (11533)

205     or/178-204 [LTC] (446735)

206     (organi#ational adj2 model?).tw,kw. (5071)

207     (model? adj3 (care or healthcare or health care or home*)).tw,kw. (113418)

208     patient care/ (307856)

209     health care delivery/ (288337)

210     integrated health care system/ (23839)

211     ((collaborat* or comprehensive* or connect* or coordinat* or co-ordinat* or integrat* or interconnect* or inter-connect*) adj3 (care or healthcare or health care or team?)).tw,kw. (149915)

212     ((interprofessional* or inter-professional*) adj3 (care or collaborat* or connect* or coordinat* or co-ordinat* or healthcare or health care or integrat* or interconnect* or inter-connect* or network* or team?)).tw,kw. (15372)

213     ((colocat* or co-locat*) adj3 (care or healthcare or health care)).tw,kw. (551)

214     health care access/ (62542)

215     (access* adj3 (care or healthcare or health care or health service?)).tw,kw. (119385)

216     (team* adj3 (care or healthcare or health care)).tw,kw. (62674)

217     (team* adj3 (approach* or model?)).tw,kw. (30035)

218     teamwork/ (18629)

219     (teamwork* or team work*).tw,kw. (40121)

220     elderly care/ (40484)

221     geriatric care/ (14671)

222     toileting/ (441)

223     (health service? adj3 (aged or elder* or geriatric* or gerontolog* or older*)).tw,kw. (3819)

224     ((dental care or dentistry or oral care or oral health or oral healthcare or oral hygiene) adj3 (aged or elder* or geriatric* or gerontolog* or older*)).tw,kw. (4371)

225     physician/ (400303)

226     general practitioner/ (116855)

227     ((clinician? or doctor? or physician? or practitioner?) adj3 family*).tw,kw. (69075)

228     ((FP or FPs or GP or GPs) adj5 (physician? or practitioner*)).tw,kw. (35324)

229     ((clinician? or doctor? or physician? or practitioner?) adj3 primary care).tw,kw. (71769)

230     ((clinician? or doctor? or physician? or practitioner?) adj3 primary healthcare).tw,kw. (678)

231     ((clinician? or doctor? or physician? or practitioner?) adj3 primary health care).tw,kw. (2474)

232     ((PCP or PCPs) adj5 (primary care or primary healthcare or primary health care)).tw,kw. (11229)

233     general practitioner?.tw,kw. (136660)

234     general practice/ (98813)

235     (family medicine or family practice? or general practice?).tw,kw. (148617)

236     (doctor? or physician?).tw,kw. (1361105)

237     physician assistant/ (13907)

238     ((clinician? or doctor? or physician? or practitioner?) adj2 (assistant* or extender*)).tw,kw. (13294)

239     feldsher?.tw,kw. (895)

240     geriatrician/ (2529)

241     (geriatrician* or gerontologist*).tw,kw. (9508)

242     nurse/ (168841)

243     (nurse or nurses).tw,kw. (674829)

244     exp advanced practice nurse/ (39215)

245     (nurs* adj2 practitioner?).tw,kw. (35172)

246     (NP adj5 (nurs* or practitioner?)).tw,kw. (2721)

247     practical nurse/ (206)

248     (nurs* adj2 practical).tw,kw. (4347)

249     registered nurse/ (43223)

250     staff nurse/ (1112)

251     nursing staff/ (93441)

252     nursing assistant/ (9855)

253     (nurs* adj2 assistant?).tw,kw. (9242)

254     nurse* aide?.tw,kw. (2254)

255     nursing auxiliar*.tw,kw. (414)

256     nurse consultant/ (299)

257     ((clinical or clinician? or consultant? or (advance? adj practic*)) adj2 nurs*).tw,kw. (45417)

258     paramedical personnel/ (26855)

259     allied health.tw,kw. (23064)

260     (healthcare assistant? or health care assistant? or healthcare aide? or health care aide? or healthcare worker? or health care worker? or HCW or HCWs).tw,kw. (60408)

261     licensed practical nurse/ (329)

262     (LPN or LPNs).tw,kw. (4150)

263     vocational nurs*.tw,kw. (476)

264     (support worker? or PSW or PSWs).tw,kw. (3992)

265     (care aide? or healthcare aide?).tw,kw. (926)

266     dentist/ (44792)

267     periodontist/ (18035)

268     (dentist or dentists or periodontist? or perio-dontist?).tw,kw. (78620)

269     dental auxiliary/ (2067)

270     (dental adj2 auxiliar*).tw,kw. (1303)

271     dental assistant/ (17330)

272     (dental adj2 assistant*).tw,kw. (3053)

273     denturist/ (131)

274     denturist*.tw,kw. (192)

275     dental hygienist/ (6132)

276     hygienist*.tw,kw. (11644)

277     dietitian/ (13368)

278     (nutritionist* or dietician* or dietitian*).tw,kw. (32836)

279     occupational therapist/ (9754)

280     (occupational adj2 therapist*).tw,kw. (21839)

281     pharmacist/ (94818)

282     pharmacist*.tw,kw. (114331)

283     physiotherapist/ (23679)

284     physiotherapist assistant/ (69)

285     (physical therapist? or physiotherapist? or physio-therapist?).tw,kw. (49027)

286     podiatrist/ (781)

287     (podiatrist? or chiropodist?).tw,kw. (2577)

288     ((podiatr* or chiropod* or foot or feet) adj3 (care or healthcare or health care or nurs* or service? or support*)).tw,kw. (8201)

289     psychologist/ (38455)

290     psychologist?.tw,kw. (133112)

291     (recreation* adj2 therapist*).tw,kw. (273)

292     social worker/ (25947)

293     (socialworker* or social worker* or social service? or social support?).tw,kw. (230997)

294     ((speech* or language*) adj2 (pathologist* or therapist*)).tw,kw. (18502)

295     personnel management/ (85674)

296     ((personnel or staff*) adj3 (mix* or compos* or compris*)).tw,kw. (3417)

297     (staff* adj2 level?).tw,kw. (8769)

298     (turnover* or turn* over*).tw,kw. (246185)

299     exp workforce/ (83769)

300     (workforce? or work force?).tw,kw. (75705)

301     manpower*.tw,kw. (19778)

302     staffing.tw,kw. (38860)

303     ((care or healthcare or health care or health service?) adj3 (mix* or compos* or compris*)).tw,kw. (11318)

304     ((dementia or memory) adj3 (care or healthcare or health care or nurs* or service? or support*)).tw,kw. (31164)

305     medicalization/ (1213)

306     medicali*.tw,kw. (8758)

307     mental health service/ (93669)

308     (mental health service? or mental health care or mental healthcare).tw,kw. (113831)

309     (mental hygiene service? or mental hygiene care or mental hygiene care).tw,kw. (188)

310     palliative therapy/ (145588)

311     palliat.tw,kw. (57)

312     pastoral care/ (3841)

313     (pastor or pastors or pastoral or minister? or priest? or rabbi? or iman?).tw,kw. (438653)

314     (spiritual* adj3 (care or comfort* or support*)).tw,kw. (10828)

315     ((eye? or ocular or optical or vision) adj3 (care or healthcare or health care or service?)).tw,kw. (16271)

316     (hearing adj3 (care or healthcare or health care or service?)).tw,kw. (2548)

317     uninsured.tw,kw. (21334)

318     (reform* adj3 (care or healthcare or health care)).tw,kw. (27512)

319     or/206-318 (5020529)

320     205 and 319 [LTC - MODELS OF CARE] (131117)

321     exp randomized controlled trial/ or controlled clinical trial/ (1399465)

322     clinical trial/ (1534231)

323     exp "controlled clinical trial (topic)"/ (189161)

324     (randomi#ed or randomi#ation? or randomly or RCT or placebo*).tw,kw. (3648137)

325     ((singl* or doubl* or trebl* or tripl*) adj (mask* or blind* or dumm*)).tw,kw. (745866)

326     trial.ti. (882698)

327     or/321-326 (5102753)

328     320 and 327 [LTC - MODELS OF CARE - RCTS] (15741)

329     controlled clinical trial/ (558605)

330     "controlled clinical trial (topic)"/ (10842)

331     (control* adj2 trial).tw,kw. (967954)

332     (nonrandom* or non-random* or quasi-random* or quasi-experiment*).tw,kw. (160649)

333     (nRCT or non-RCT).tw,kw. (925)

334     (control* adj3 ("before and after" or "before after")).tw,kw. (744411)

335     time series analysis/ (26307)

336     time series.tw,kw. (76752)

337     pretest posttest control group design/ (471)

338     (pre- adj3 post-).tw,kw. (291802)

339     (pretest adj3 posttest).tw,kw. (25468)

340     controlled study/ (7514602)

341     (control* adj2 study).tw,kw. (900048)

342     control group/ (125788)

343     (control* adj2 group?).tw,kw. (1547106)

344     trial.ti. (882698)

345     or/329-344 (10544174)

346     320 and 345 [LTC - MODELS OF CARE - nRCTS] (21957)

347     328 or 346 [RCTs, nRCTs] (27489)

348     limit 347 to yr="2010-current" (16577)

349     exp animal/ or exp animal experimentation/ or exp animal model/ or exp animal experiment/ or nonhuman/ or exp vertebrate/ (53923769)

350     exp human/ or exp human experimentation/ or exp human experiment/ (41587698)

351     349 not 350 (12337828)

352     348 not 351 [ANIMAL-ONLY REMOVED] (16469)

353     editorial.pt. (1193246)

354     letter.pt. not (letter.pt. and randomized controlled trial/) (2208171)

355     352 not (353 or 354) [OPINION PIECES REMOVED] (16371)

356     conference abstract.pt. (3837009)

357     355 not 356 [CONFERENCE ABSTRACTS REMOVED] (13082)

358     357 use emczd [EMBASE RECORDS] (6933)

359     Long-Term Care/ (162933)

360     ((longterm or long-term) adj3 (care or facility or facilities or healthcare or health care or institution or institutions)).tw,id. (70896)

361     (LTC or LTCF or LTCFs or RACF or RACFs).tw,id. (11825)

362     (care home or care homes).tw,id. (11727)

363     (care facility or care facilities).tw,id. (58531)

364     (residential adj (care or facility or facilities or healthcare or health care)).tw,id. (15453)

365     (SNF or SNFs).tw,id. (10707)

366     Institutionalization/ (18368)

367     (institutionali#ed or institutionali#ation*).tw,id. (52169)

368     home? for the aged.tw,id. (4305)

369     home? for the elderly.tw,id. (4069)

370     ((facility or facilities) adj "for the aged").tw,id. (69)

371     ((facility or facilities) adj "for the elderly").tw,id. (49)

372     institution? for the aged.tw,id. (466)

373     institution? for the elderly.tw,id. (588)

374     ("old age" adj (facility or facilities or home or homes or institution?)).tw,id. (974)

375     (("aged care" or "aged healthcare" or "aged health care") adj3 (facility or facilities or home or homes or institution?)).tw,id. (3188)

376     (("elder care" or "elder healthcare" or "elder health care") adj3 (facility or facilities or home or homes or institution?)).tw,id. (181)

377     (("geriatric care" or "geriatric healthcare" or "geriatric health care") adj3 (facility or facilities or home or homes or institution?)).tw,id. (326)

378     (("psychogeriatric care" or "psychogeriatric healthcare" or "psychogeriatric health care") adj3 (facility or facilities or home or homes or institution?)).tw,id. (16)

379     (("gerontologic care" or "gerontologic healthcare" or "gerontologic health care") adj3 (facility or facilities or home or homes or institution?)).tw,id. (0)

380     (("gerontological care" or "gerontological healthcare" or "gerontological health care") adj3 (facility or facilities or home or homes or institution?)).tw,id. (5)

381     exp Nursing Homes/ (103771)

382     nursing home?.tw,id. (84711)

383     (nursing facility or nursing facilities).tw,id. (11409)

384     or/359-383 [LTC] (437545)

385     (organi#ational adj2 model?).tw,id. (4988)

386     (model? adj3 (care or healthcare or health care or home*)).tw,id. (111601)

387     Health Care Delivery/ (288337)

388     Integrated Services/ (3730)

389     ((collaborat* or comprehensive* or connect* or coordinat* or co-ordinat* or integrat* or interconnect* or inter-connect*) adj3 (care or healthcare or health care or team?)).tw,id. (148243)

390     ((interprofessional* or inter-professional*) adj3 (care or collaborat* or connect* or coordinat* or co-ordinat* or healthcare or health care or integrat* or interconnect* or inter-connect* or network* or team?)).tw,id. (14914)

391     ((colocat* or co-locat*) adj3 (care or healthcare or health care)).tw,id. (550)

392     Health Care Access/ (62542)

393     (access* adj3 (care or healthcare or health care or health service?)).tw,id. (115901)

394     Continuum of Care/ (21516)

395     (team* adj3 (care or healthcare or health care)).tw,id. (61634)

396     (team* adj3 (approach* or model?)).tw,id. (29954)

397     (teamwork* or team work*).tw,id. (38931)

398     (health service? adj3 (aged or elder* or geriatric* or gerontolog* or older*)).tw,id. (3277)

399     ((dental care or dentistry or oral care or oral health or oral healthcare or oral hygiene) adj3 (aged or elder* or geriatric* or gerontolog* or older*)).tw,id. (4007)

400     Physicians/ (308844)

401     General Practitioners/ (111384)

402     Family Physicians/ (18355)

403     ((clinician? or doctor? or physician? or practitioner?) adj3 family*).tw,id. (67756)

404     ((FP or FPs or GP or GPs) adj5 (physician? or practitioner*)).tw,id. (35270)

405     ((clinician? or doctor? or physician? or practitioner?) adj3 primary care).tw,id. (71526)

406     ((clinician? or doctor? or physician? or practitioner?) adj3 primary healthcare).tw,id. (671)

407     ((clinician? or doctor? or physician? or practitioner?) adj3 primary health care).tw,id. (2238)

408     ((PCP or PCPs) adj5 (primary care or primary healthcare or primary health care)).tw,id. (11216)

409     general practitioner?.tw,id. (134522)

410     Family Medicine/ (12223)

411     (family medicine or family practice? or general practice?).tw,id. (141825)

412     (doctor? or physician?).tw,id. (1349729)

413     ((clinician? or doctor? or physician? or practitioner?) adj2 (assistant* or extender*)).tw,id. (13159)

414     feldsher?.tw,id. (893)

415     (geriatrician* or gerontologist*).tw,id. (9476)

416     Nurses/ (165341)

417     (nurse or nurses).tw,id. (670740)

418     (nurs* adj2 practitioner?).tw,id. (34730)

419     (NP adj5 (nurs* or practitioner?)).tw,id. (2709)

420     (nurs* adj2 practical).tw,id. (4315)

421     (nurs* adj2 assistant?).tw,id. (9153)

422     nurse* aide?.tw,id. (2102)

423     nursing auxiliar*.tw,id. (408)

424     ((clinical or clinician? or consultant? or (advance? adj practic*)) adj2 nurs*).tw,id. (44424)

425     Allied Health Personnel/ (27606)

426     allied health.tw,id. (22632)

427     (healthcare assistant? or health care assistant? or healthcare aide? or health care aide? or healthcare worker? or health care worker? or HCW or HCWs).tw,id. (59578)

428     (LPN or LPNs).tw,id. (4135)

429     vocational nurs*.tw,id. (476)

430     (support worker? or PSW or PSWs).tw,id. (3944)

431     (care aide? or healthcare aide?).tw,id. (917)

432     Dentists/ (41830)

433     (dentist or dentists or periodontist? or perio-dontist?).tw,id. (78288)

434     (dental adj2 auxiliar*).tw,id. (1293)

435     (dental adj2 assistant*).tw,id. (3027)

436     denturist*.tw,id. (192)

437     hygienist*.tw,id. (11571)

438     (nutritionist* or dietician* or dietitian*).tw,id. (32517)

439     Occupational Therapists/ (9436)

440     (occupational adj2 therapist*).tw,id. (21750)

441     Pharmacists/ (94818)

442     pharmacist*.tw,id. (113244)

443     Physical Therapists/ (22870)

444     (physical therapist? or physiotherapist? or physio-therapist?).tw,id. (48661)

445     (podiatrist? or chiropodist?).tw,id. (2562)

446     ((podiatr* or chiropod* or foot or feet) adj3 (care or healthcare or health care or nurs* or service? or support*)).tw,id. (8067)

447     Psychologists/ (45982)

448     psychologist?.tw,id. (132959)

449     (recreation* adj2 therapist*).tw,id. (272)

450     Social Workers/ (23708)

451     (socialworker* or social worker* or social service? or social support?).tw,id. (225875)

452     Speech Therapists/ (1179)

453     ((speech* or language*) adj2 (pathologist* or therapist*)).tw,id. (18424)

454     Human Resource Management/ (11456)

455     Employee Turnover/ (10380)

456     Personnel Supply/ (366)

457     Work Scheduling/ (1624)

458     ((personnel or staff*) adj3 (mix* or compos* or compris*)).tw,id. (3404)

459     (staff* adj2 level?).tw,id. (8742)

460     (turnover* or turn* over*).tw,id. (243873)

461     (workforce? or work force?).tw,id. (73667)

462     manpower*.tw,id. (18758)

463     staffing.tw,id. (38466)

464     ((care or healthcare or health care or health service?) adj3 (mix* or compos* or compris*)).tw,id. (11230)

465     ((dementia or memory) adj3 (care or healthcare or health care or nurs* or service? or support*)).tw,id. (30305)

466     medicali*.tw,id. (8477)

467     Mental Health Services/ (123196)

468     (mental health service? or mental health care or mental healthcare).tw,id. (111058)

469     (mental hygiene service? or mental hygiene care or mental hygiene care).tw,id. (98)

470     Palliative Care/ (142914)

471     palliat.tw,id. (57)

472     Pastoral Counseling/ (2314)

473     (pastor or pastors or pastoral or minister? or priest? or rabbi? or iman?).tw,id. (434814)

474     (spiritual* adj3 (care or comfort* or support*)).tw,id. (10670)

475     ((eye? or ocular or optical or vision) adj3 (care or healthcare or health care or service?)).tw,id. (16120)

476     (hearing adj3 (care or healthcare or health care or service?)).tw,id. (2473)

477     uninsured.tw,id. (21140)

478     Health Care Reform/ (209309)

479     (reform* adj3 (care or healthcare or health care)).tw,id. (26520)

480     or/385-479 (4717509)

481     384 and 480 [LTC - MODELS OF CARE] (113803)

482     Randomized Controlled Trials/ (316771)

483     Randomized Clinical Trials/ (147)

484     Clinical Trials/ (105547)

485     Placebo/ (368415)

486     (randomi#ed or randomi#ation? or randomly or RCT or placebo*).tw,id. (3585510)

487     ((singl* or doubl* or trebl* or tripl*) adj (mask* or blind* or dumm*)).tw,id. (718735)

488     trial.ti. (882698)

489     or/482-488 (4182228)

490     481 and 489 [LTC - MODELS OF CARE - RCTS] (11312)

491     (control* adj2 trial).tw,id. (645025)

492     (nonrandom* or non-random* or quasi-random* or quasi-experiment*).tw,id. (159549)

493     (nRCT or non-RCT).tw,id. (923)

494     (control* adj3 ("before and after" or "before after")).tw,id. (744397)

495     Time Series/ (26831)

496     time series.tw,id. (75176)

497     (pre- adj3 post-).tw,id. (291661)

498     (pretest adj3 posttest).tw,id. (22507)

499     (control* adj2 study).tw,id. (535066)

500     Experiment Controls/ (918)

501     (control* adj2 group?).tw,id. (1545689)

502     trial.ti. (882698)

503     or/491-502 (3576683)

504     481 and 503 [LTC - MODELS OF CARE - nRCTS] (10300)

505     490 or 504 [RCTs, nRCTs] (14775)

506     limit 505 to yr="2010-current" (9577)

507     limit 506 to ("comment/reply" or dissertation or editorial or letter) [Limit not valid in Embase,Ovid MEDLINE(R),Ovid MEDLINE(R) Daily Update,Ovid MEDLINE(R) In-Process,Ovid MEDLINE(R) Publisher,CCTR; records were retained] (141)

508     506 not 507 [OPINION PIECES, DISSERTATIONS REMOVED] (9436)

509     508 use medall,emczd,cctr (8844)

510     508 not 509 [PSYCINFO RECORDS] (592)

511     Long-Term Care/ (162933)

512     ((longterm or long-term) adj3 (care or facility or facilities or healthcare or health care or institution or institutions)).ti,ab,kw. (75151)

513     (LTC or LTCF or LTCFs or RACF or RACFs).ti,ab,kw. (11874)

514     (care home or care homes).ti,ab,kw. (11878)

515     (care facility or care facilities).ti,ab,kw. (59467)

516     (residential adj (care or facility or facilities or healthcare or health care)).ti,ab,kw. (15235)

517     (SNF or SNFs).ti,ab,kw. (10853)

518     Institutionalization/ (18368)

519     (institutionali#ed or institutionali#ation*).ti,ab,kw. (51274)

520     Homes for the Aged/ (26653)

521     home? for the aged.ti,ab,kw. (4592)

522     home? for the elderly.ti,ab,kw. (4074)

523     ((facility or facilities) adj "for the aged").ti,ab,kw. (69)

524     ((facility or facilities) adj "for the elderly").ti,ab,kw. (57)

525     institution? for the aged.ti,ab,kw. (466)

526     institution? for the elderly.ti,ab,kw. (590)

527     ("old age" adj (facility or facilities or home or homes or institution?)).ti,ab,kw. (972)

528     (("aged care" or "aged healthcare" or "aged health care") adj3 (facility or facilities or home or homes or institution?)).ti,ab,kw. (3193)

529     (("elder care" or "elder healthcare" or "elder health care") adj3 (facility or facilities or home or homes or institution?)).ti,ab,kw. (178)

530     (("geriatric care" or "geriatric healthcare" or "geriatric health care") adj3 (facility or facilities or home or homes or institution?)).ti,ab,kw. (341)

531     (("psychogeriatric care" or "psychogeriatric healthcare" or "psychogeriatric health care") adj3 (facility or facilities or home or homes or institution?)).ti,ab,kw. (17)

532     (("gerontologic care" or "gerontologic healthcare" or "gerontologic health care") adj3 (facility or facilities or home or homes or institution?)).ti,ab,kw. (4)

533     (("gerontological care" or "gerontological healthcare" or "gerontological health care") adj3 (facility or facilities or home or homes or institution?)).ti,ab,kw. (5)

534     exp Nursing Homes/ (103771)

535     nursing home?.ti,ab,kw. (86297)

536     (nursing facility or nursing facilities).ti,ab,kw. (11498)

537     or/511-536 [LTC] (447241)

538     Models, Organizational/ (64427)

539     (organi#ational adj2 model?).ti,ab,kw. (4855)

540     (model? adj3 (care or healthcare or health care or home*)).ti,ab,kw. (112996)

541     Patient Care Management/ (285337)

542     Delivery of Health Care/ (230543)

543     exp "Delivery of Health Care, Integrated"/ (24368)

544     ((collaborat* or comprehensive* or connect* or coordinat* or co-ordinat* or integrat* or interconnect* or inter-connect*) adj3 (care or healthcare or health care or team?)).ti,ab,kw. (149095)

545     ((interprofessional* or inter-professional*) adj3 (care or collaborat* or connect* or coordinat* or co-ordinat* or healthcare or health care or integrat* or interconnect* or inter-connect* or network* or team?)).ti,ab,kw. (15230)

546     ((colocat* or co-locat*) adj3 (care or healthcare or health care)).ti,ab,kw. (551)

547     Health Services Accessibility/ (122716)

548     (access* adj3 (care or healthcare or health care or health service?)).ti,ab,kw. (118671)

549     Continuity of Patient Care/ (284125)

550     Patient Care Team/ (277697)

551     (team* adj3 (care or healthcare or health care)).ti,ab,kw. (62464)

552     (team* adj3 (approach* or model?)).ti,ab,kw. (29787)

553     (teamwork* or team work*).ti,ab,kw. (39569)

554     Health Services for the Aged/ (54133)

555     (health service? adj3 (aged or elder* or geriatric* or gerontolog* or older*)).ti,ab,kw. (3690)

556     Dental Care for Aged/ (23625)

557     ((dental care or dentistry or oral care or oral health or oral healthcare or oral hygiene) adj3 (aged or elder* or geriatric* or gerontolog* or older*)).ti,ab,kw. (4361)

558     Physicians/ (308844)

559     General Practitioners/ (111384)

560     Physicians, Family/ (114192)

561     Physicians, Primary Care/ (106716)

562     ((clinician? or doctor? or physician? or practitioner?) adj3 family*).ti,ab,kw. (68813)

563     ((FP or FPs or GP or GPs) adj5 (physician? or practitioner*)).ti,ab,kw. (35316)

564     ((clinician? or doctor? or physician? or practitioner?) adj3 primary care).ti,ab,kw. (71574)

565     ((clinician? or doctor? or physician? or practitioner?) adj3 primary healthcare).ti,ab,kw. (676)

566     ((clinician? or doctor? or physician? or practitioner?) adj3 primary health care).ti,ab,kw. (2448)

567     ((PCP or PCPs) adj5 (primary care or primary healthcare or primary health care)).ti,ab,kw. (11227)

568     general practitioner?.ti,ab,kw. (135962)

569     Family Practice/ (149251)

570     General Practice/ (98813)

571     (family medicine or family practice? or general practice?).ti,ab,kw. (148015)

572     (doctor? or physician?).ti,ab,kw. (1358734)

573     Physician Assistants/ (11837)

574     ((clinician? or doctor? or physician? or practitioner?) adj2 (assistant* or extender*)).ti,ab,kw. (13274)

575     feldsher?.ti,ab,kw. (895)

576     Geriatricians/ (2532)

577     (geriatrician* or gerontologist*).ti,ab,kw. (9496)

578     Nurses/ (165341)

579     (nurse or nurses).ti,ab,kw. (672782)

580     Nurse Practitioners/ (38980)

581     (nurs* adj2 practitioner?).ti,ab,kw. (35021)

582     (NP adj5 (nurs* or practitioner?)).ti,ab,kw. (2721)

583     Nursing, Practical/ (3589)

584     (nurs* adj2 practical).ti,ab,kw. (4319)

585     Nursing Assistants/ (9919)

586     (nurs* adj2 assistant?).ti,ab,kw. (9212)

587     nurse* aide?.ti,ab,kw. (2223)

588     nursing auxiliar*.ti,ab,kw. (414)

589     Nursing Staff/ (93441)

590     Nurse Clinicians/ (10302)

591     Advanced Practice Nursing/ (3538)

592     ((clinical or clinician? or consultant? or (advance? adj practic*)) adj2 nurs*).ti,ab,kw. (45218)

593     Allied Health Personnel/ (27606)

594     allied health.ti,ab,kw. (22983)

595     (healthcare assistant? or health care assistant? or healthcare aide? or health care aide? or healthcare worker? or health care worker? or HCW or HCWs).ti,ab,kw. (60260)

596     Licensed Practical Nurses/ (329)

597     (LPN or LPNs).ti,ab,kw. (4146)

598     vocational nurs*.ti,ab,kw. (467)

599     (support worker? or PSW or PSWs).ti,ab,kw. (3957)

600     (care aide? or healthcare aide?).ti,ab,kw. (919)

601     Dentists/ (41830)

602     (dentist or dentists or periodontist? or perio-dontist?).ti,ab,kw. (78560)

603     exp Dental Auxiliaries/ (26538)

604     (dental adj2 auxiliar*).ti,ab,kw. (1301)

605     (dental adj2 assistant*).ti,ab,kw. (3048)

606     denturist*.ti,ab,kw. (192)

607     hygienist*.ti,ab,kw. (11633)

608     Nutritionists/ (13368)

609     (nutritionist* or dietician* or dietitian*).ti,ab,kw. (32820)

610     Occupational Therapists/ (9436)

611     (occupational adj2 therapist*).ti,ab,kw. (21662)

612     Pharmacists/ (94818)

613     pharmacist*.ti,ab,kw. (114276)

614     Physical Therapists/ (22870)

615     Physical Therapist Assistants/ (83)

616     (physical therapist? or physiotherapist? or physio-therapist?).ti,ab,kw. (48986)

617     (podiatrist? or chiropodist?).ti,ab,kw. (2573)

618     ((podiatr* or chiropod* or foot or feet) adj3 (care or healthcare or health care or nurs* or service? or support*)).ti,ab,kw. (8194)

619     psychologist?.ti,ab,kw. (129752)

620     (recreation* adj2 therapist*).ti,ab,kw. (267)

621     Social Workers/ (23708)

622     (socialworker* or social worker* or social service? or social support?).ti,ab,kw. (225176)

623     ((speech* or language*) adj2 (pathologist* or therapist*)).ti,ab,kw. (18352)

624     "Personnel Staffing and Scheduling"/ (71738)

625     Personnel Turnover/ (64976)

626     ((personnel or staff*) adj3 (mix* or compos* or compris*)).ti,ab,kw. (3396)

627     (staff* adj2 level?).ti,ab,kw. (8747)

628     (turnover* or turn* over*).ti,ab,kw. (245781)

629     exp Workforce/ (83769)

630     (workforce? or work force?).ti,ab,kw. (75035)

631     manpower*.ti,ab,kw. (19706)

632     staffing.ti,ab,kw. (38668)

633     ((care or healthcare or health care or health service?) adj3 (mix* or compos* or compris*)).ti,ab,kw. (11304)

634     exp Dementia/nu [nursing] (6288)

635     ((dementia or memory) adj3 (care or healthcare or health care or nurs* or service? or support*)).ti,ab,kw. (30872)

636     Medicalization/ (1213)

637     medicali*.ti,ab,kw. (8596)

638     Mental Health Services/ (123196)

639     (mental health service? or mental health care or mental healthcare).ti,ab,kw. (109549)

640     (mental hygiene service? or mental hygiene care or mental hygiene care).ti,ab,kw. (182)

641     Palliative Care/ (142914)

642     palliat.ti,ab,kw. (57)

643     Pastoral Care/ (3841)

644     (pastor or pastors or pastoral or minister? or priest? or rabbi? or iman?).ti,ab,kw. (437339)

645     (spiritual* adj3 (care or comfort* or support*)).ti,ab,kw. (10702)

646     ((eye? or ocular or optical or vision) adj3 (care or healthcare or health care or service?)).ti,ab,kw. (16239)

647     (hearing adj3 (care or healthcare or health care or service?)).ti,ab,kw. (2514)

648     uninsured.ti,ab,kw. (21306)

649     Health Care Reform/ (209309)

650     (reform* adj3 (care or healthcare or health care)).ti,ab,kw. (27107)

651     or/538-650 (5207786)

652     537 and 651 [LTC - MODELS OF CARE] (135582)

653     limit 652 to yr="2010-current" (64722)

654     conference abstract.pt. (3837009)

655     "Journal: Conference Abstract".pt. (157141)

656     653 not (654 or 655) [CONFERENCE ABSTRACTS REMOVED] (54334)

657     656 use cctr [CENTRAL RECORDS] (3026)

658     177 or 358 or 510 or 657 [ALL DATABASES] (12896)

659     limit 658 to yr="2017-current" (5526)

660     remove duplicates from 659 (4172)

661     limit 658 to yr="2012-2016" (5569)

662     remove duplicates from 661 (3879)

663     658 not (659 or 661) (1801)

664     remove duplicates from 663 (1259)

665     660 or 662 or 664 (9310) [TOTAL UNIQUE RECORDS]

666     665 use medall [MEDLINE UNIQUE RECORDS] (2324)

667     665 use emczd [EMBASE UNIQUE RECORDS] (5004)

668     665 use medall,emczd,cctr (9193)

669     665 not 668 [PSYCINFO UNIQUE RECORDS] (117)

670     665 use cctr [CENTRAL UNIQUE RECORDS] (1865)
