# Supplementary material for: Models of provider care in long-term care: A rapid scoping review
Source: PLoS One. 2021 Jul 16;16(7):e0254527. doi: 10.1371/journal.pone.0254527 (PMC8284811; doi:10.1371/journal.pone.0254527)
Supplement: S5 File — This includes the extraction forms details for the mapping and charting extractions. (DOCX) [file pone.0254527.s005.docx]

# S5 File. Data collection forms

## Mapping

The following information was collected:

- Country where the study took place
- Intervention/Model/Program/Study name or keyword description
- Mapped to either healthcare service delivery or implementation strategy
- Type of care provided/ intervention delivered including a description of the care providers involved
- A brief narrative description of the model, service or intervention
- Study design: RCT/non-RCT (including a controlled before-after study), a CBA or ITS, or a comparative cohort
- What category the intervention should be mapped to (i.e., primary care specialists/ team members, primary care, direct patient care, allied health care providers, hospital-related focus/outcomes, support multidisciplinary teams, targeting specific conditions/risk factors). These categories were not mutually exclusive.
- What condition should the intervention be mapped to (e.g., oral health care, dementia care, cognitive/ mental health, diabetes, exercise)
- For healthcare service delivery interventions: were there hospital-related outcomes reported (e.g., transfer, ED visits, acute care admission length of stay)?
- Would need to follow-up with the author to determine if new member/change to existing role? (this would help determine those that were unclear if they should be mapped to healthcare service delivery or implementation strategies)

## Charting

**Healthcare service delivery**

- Details of the residents (e.g., number of residents)
- Details of the intervention
  - Number of LTCH
  - Number of participants
  - Brief description of the intervention
- Details of the comparison
  - Number of LTCH
  - Number of participants
  - Brief description of the comparison group
- Outcomes reported (e.g., quality of life, quality of care, health outcomes)
- Summary of the main conclusions
