# Supplementary material for: Models of provider care in long-term care: A rapid scoping review
Source: PLoS One. 2021 Jul 16;16(7):e0254527. doi: 10.1371/journal.pone.0254527 (PMC8284811; doi:10.1371/journal.pone.0254527)
Supplement: S6 File — (DOCX) [file pone.0254527.s006.docx]

# S6 File. Included studies mapping and details

Contents

[S6 Appendix. Studies mapping and Study details 1](#_Toc65096772)

[S6 Appendix, Section 1: Studies mapping 2](#_Toc65096773)

[S6 Appendix, Section 2: Healthcare Service Delivery studies 6](#_Toc65096774)

[Table A: Access to specialty physician care within long-term care 6](#_Toc65096775)

[Table B: Models for primary care within Long-Term Care 11](#_Toc65096776)

[Table C: Models for direct patient care 15](#_Toc65096777)

[Table D: Allied health care teams 20](#_Toc65096778)

[Table E: Models for preventing acute care hospital admission and readmission 35](#_Toc65096779)

[Table F: Models of care focused on specific conditions/interventions 36](#_Toc65096780)

[S6 Appendix, Section 3: Healthcare Services Delivery: multidisciplinary studies 38](#_Toc65096781)

[Table A&B: Specialist & Primary care 38](#_Toc65096782)

[Table A&C: Specialist & Direct patient care 40](#_Toc65096783)

[Table A&D: Specialist & Allied health care 42](#_Toc65096784)

[Table A&C&D: Specialist & Direct patient care & Allied health care 45](#_Toc65096785)

[Table B&C: Primary care & Direct patient care 46](#_Toc65096786)

[Table B&D: Direct patient care & Allied health care 51](#_Toc65096787)

[Bibliography of included studies 52](#_Toc65096788)

## Section 1: Studies mapping

Table 1 presents the 92 included studies, sorted in alphabetical order. It provides which tables each study may be located.

Table 1 - Studies mapping

| Author Year | Country | Study Design | Table | Subsection |
| --- | --- | --- | --- | --- |
| Arendts 2018 (1) | Australia | RCT/non-RCT | B (E) | Overall care |
| Azermai 2017† (2) | Belgium | CBA or ITS | C&D (G) | Optimal/appropriate medication use |
| Balsom 2019 (3) | Canada | RCT/non-RCT | D | Optimal/appropriate medication use |
| Barbe 2019 (4) | Germany | RCT/non-RCT | D (H) | Oral health |
| Barbe 2020 (5) | Germany | RCT/non-RCT | D (H) | Oral health |
| Beaupre 2020 (6) | Canada | Comparative Cohort | D | Hip fracture |
| Beck 2016 (7) | Denmark | RCT/non-RCT | D | Nutrition |
| Boorsma 2011 (8) | The Netherlands | RCT/non-RCT | C | Overall care |
| Borbasi 2011 (9) | Australia | Comparative Cohort | F | Dementia care, incl. agitation |
| Boyd 2014 (10) | New Zealand | RCT/non-RCT | C (E) | Overall care |
| Brett 2019† (11) | Australia | RCT/non-RCT | D | Exercise/mobility |
| Chapman 2018 (12) | Australia | RCT/non-RCT | B&C (E) | Palliative care |
| Connolly 2015 (13) | New Zealand | RCT/non-RCT | C (E) | Overall care |
| Connolly 2016 (14) | New Zealand | RCT/non-RCT | C (E) | Overall care |
| Connolly 2018 (15) | New Zealand | CBA or ITS | C (E) | Overall care |
| Conway 2015 (16) | Australia | CBA or ITS | C, E | Overall care |
| Cool 2018 (17) | France | RCT/non-RCT | A (G) | Optimal/appropriate medication use |
| Cordato 2018 (18) | Australia | RCT/non-RCT | A&B (E) | Overall care |
| Craswell 2020 (19) | Australia | Comparative Cohort | B (E) | Overall care |
| Crotty 2019 (20) | Australia | RCT/non-RCT | A&D | Hip fracture |
| D'Arcy 2013 (21) | USA | Comparative Cohort | A (E) | Overall care |
| De Luca 2016 (22) | Italy | RCT/non-RCT | A&D | Overall care |
| de Souto Barreto 2016 (23) | France | RCT/non-RCT | A (G) | Optimal/appropriate medication use |
| Doernberg 2015 (24) | USA | CBA or ITS | A&D | Optimal/appropriate medication use |
| Dorfman 2020 (25) | Canada | Comparative Cohort | D | Optimal/appropriate medication use |
| El Haddad 2020 (26) | France | Comparative Cohort | A (G) | Optimal/appropriate medication use |
| Forbat 2020 (27) | Australia | RCT/non-RCT | B&C (E) | Palliative care |
| Gloth 2011 (28) | USA | Comparative Cohort | A (E) | Overall care |
| Grabowski 2014 (29) | USA | Comparative Cohort | B&C (E) | Overall care |
| Guion 2018 (30) | France | RCT/non-RCT | A (G) | Optimal/appropriate medication use |
| Haines 2020 (31) | Australia | RCT/non-RCT | B&C (E) | Overall care |
| Harvey 2014 (32) | Australia | RCT/non-RCT | A&C (E) | Overall care |
| Hashimoto 2020 (33) | Japan | RCT/non-RCT | D | Optimal/appropriate medication use |
| Henskens 2017 (34) | The Netherlands | RCT/non-RCT | F | Dementia care, incl. agitation |
| Hewitt 2019† (35) | Australia | RCT/non-RCT | D | Exercise/mobility |
| Hopper 2016 (36) | Canada | RCT/non-RCT | D | Hearing care |
| Hullick 2016 (37) | Australia | CBA or ITS | C (E) | Overall care |
| Hutchinson 2015 (38) | Australia | CBA or ITS | A&C (E) | Overall care |
| Justine 2010 (39) | Malaysia | Comparative Cohort | D | Exercise/mobility |
| Kaasalainen 2016† (40) | Canada | CBA or ITS | B (F) | Pain management |
| Kane 2017 (41) | USA | RCT/non-RCT | E | Overall care |
| Killington 2020 (42) | Australia | RCT/non-RCT | A&D | Hip fracture |
| Kobewka 2020 (43) | Canada | Comparative Cohort | B (E) | Overall care |
| Kulakçi 2013 (44) | Turkey | CBA or ITS | C | Overall care |
| Lacny 2016 (45) | Canada | CBA or ITS | B (E) | Overall care |
| Laffon de Mazières 2019 (46) | France | RCT/non-RCT | A (G) | Optimal/appropriate medication use |
| Lin 2010 (47) | Taiwan | RCT/non-RCT | D (F) | Nutrition |
| Lindelof 2013 (48) | Sweden | RCT/non-RCT | D | Exercise/mobility |
| Liu 2020 (49) | Australia | RCT/non-RCT | B&C (E) | Palliative care |
| Man 2020 (50) | Australia | RCT/non-RCT | D | Vision care |
| Marchini 2018 (51) | USA | RCT/non-RCT | D (H) | Oral health |
| McCarthy 2019 (52) | UK | CBA or ITS | B&C (E) | Overall care |
| McDerby 2019 (53) | Australia | RCT/non-RCT | D | Optimal/appropriate medication use |
| McDerby 2020 (54) | Australia | RCT/non-RCT | D | Optimal/appropriate medication use |
| McSweeney 2012 (55) | Australia | RCT/non-RCT | A&D | Depression |
| Miller 2016 (56) | USA | Comparative Cohort | A&B (E) | Palliative care |
| Miller 2017 (57) | USA | Comparative Cohort | A&B (E) | Palliative care |
| Morciano 2020 (58) | UK | RCT/non-RCT | B&C (E) | Overall care |
| Morino 2014 (59) | Japan | RCT/non-RCT | D | Oral health |
| Moyle 2014 (60) | Australia | RCT/non-RCT | D (F) | Dementia care, incl. agitation |
| Moyle 2014 (61) | Australia | RCT/non-RCT | D (F) | Dementia care, incl. agitation |
| Moyle 2016 (62) | Australia | RCT/non-RCT | F (G) | Dementia care, incl. agitation |
| Nishiyama 2010 (63) | Japan | RCT/non-RCT | D | Oral health |
| Patterson 2010 (64) | Northern Ireland | RCT/non-RCT | D | Optimal/appropriate medication use |
| Pedersen 2017 (65) | Denmark | RCT/non-RCT | B&C (E) | Overall care |
| Pedersen 2018 (66) | Denmark | RCT/non-RCT | B&C (E) | Overall care |
| Rantz 2018† (67) | USA | Comparative Cohort | C | Overall care |
| Rapp 2013† (68) | Germany | RCT/non-RCT | A&C (F) | Dementia care, incl. agitation |
| Rodriguez-Mansilla 2013 (69) | Spain | RCT/non-RCT | D | Dementia care, incl. agitation |
| Rolland 2016 (70) | France | RCT/non-RCT | A (G) | Optimal/appropriate medication use |
| Rolland 2020 (71) | France | RCT/non-RCT | A (E) | Overall care |
| Sackley 2016 (72) | UK | RCT/non-RCT | D | Overall care: stroke-related disabilities |
| Seleskog 2018 (73) | Sweden | RCT/non-RCT | D | Oral health |
| Sluggett 2020 (74) | Australia | RCT/non-RCT | D | Optimal/appropriate medication use |
| Snider 2012 (75) | USA | RCT/non-RCT | A | Exercise/Mobility |
| Sumi 2010 (76) | Japan | RCT/non-RCT | D | Oral health |
| Telenius 2015 (77) | Norway | RCT/non-RCT | D | Exercise/mobility |
| Telenius 2015 (78) | Norway | RCT/non-RCT | D | Exercise/mobility |
| Temkin-Greener 2017 (79) | USA | RCT/non-RCT | B (G) | Palliative care |
| Temkin-Greener 2018 (80) | USA | RCT/non-RCT | B (G) | Palliative care |
| Törmä 2015 (81) | Sweden | Comparative Cohort | D | Nutrition |
| Travers 2017 (82) | Australia | RCT/non-RCT | D | Depression |
| Tynan 2018 (83) | Australia | CBA or ITS | D | Oral health |
| Verkaik 2011 (84) | The Netherlands | RCT/non-RCT | C (F) | Depression |
| Verrue 2012 (85) | Belgium | RCT/non-RCT | A&D | Optimal/appropriate medication use |
| Weatherall 2019 (86) | Denmark | Comparative Cohort | B (E) | Overall care |
| Wenborn 2013 (87) | UK | RCT/non-RCT | D | Activity involvement |
| Wikström 2017 (88) | Sweden | RCT/non-RCT | D (H) | Oral health |
| Wu 2010 (89) | Taiwan | RCT/non-RCT | A&C&D | Overall care |
| Wylie 2017 (90) | Scotland | RCT/non-RCT | D | Foot care |
| Zenthofer 2013 (91) | Germany | RCT/non-RCT | D (H) | Oral health |
| Zenthofer 2016 (92) | Germany | RCT/non-RCT | D (H) | Oral health |

## Section 2: Healthcare Service Delivery studies

### Table A: Access to specialty physician care within long-term care

| **Author Year**  **Study design**  **Country** | **Participants, Intervention, Comparison, Outcomes (PICO)** | **Outcomes** | **Main conclusions** |
| --- | --- | --- | --- |
| **exercise/mobility** | | | |
| **Snider 2012,** USA  RCT/non-RCT | **Inclusion:** Men and women aged 65 to 100 years who were residents of 1 of 2 area nursing homes in Kirksville, Missouri, were eligible.  **Profit status of NH:** NR  **Intervention:** 2 nursing homes, 8 residents in Osteopathic manipulative treatment (OMT), 6 residents in light touch (LT). Participants in the OMT (provided by licensed osteopathic physicians) and LT groups received a focused musculoskeletal physical examination twice a month for 5 months (10 visits). This physical examination included evaluation of the cervical, thoracic, and lumbar spine; sacrum; pelvis; and ribs.  **Comparison:** N/A, 7 residents. Treatment as usual. | **QoC/health outcomes:** Activities of daily living (ADL) dependence, cognition, delirium, mood, passive range of motion and voluntary movement, falls, pain symptoms, hospitalizations, emergency room visits, mortality, stability of conditions/ diseases, number of medications, overall changes in care needs. | - In the current pilot study, OMT and LT protocols that were administered twice per month reduced hospitalizations and decreased medication usage in elderly nursing home participants. - A future study with a larger number of participants and refined protocols is needed to determine the impact of OMT on the morbidity and mortality of this population. |
| **Optimal/appropriate medication use** | | | |
| **Cool 2018,** France  RCT/non-RCT  *G. Imp. Strategies to support multi-disciplinary teams* | **Inclusion:** Residents available at the follow-up time point.  **Profit status of NH:** Mix of for profit and not for profit.  **Intervention**: 77 nursing homes, 459 residents. Audit and feedback intervention on quality indicators associated to cooperative work meetings between hospital geriatricians and NH staff.  **Comparison:** 86 nursing homes, 515 residents. Audit and feedback only. | **QoC/health outcomes**: Contraindications and drug–drug interactions; potentially inappropriate drug prescribing (defined as the presence of at least one of four criteria); each component of the primary outcome. | - IQUARE significantly reduced potentially inappropriate drug prescribing in NH residents. - This study provides important aspects that should be considered when constructing further new studies seeking to change prescribing patterns and to reduce the total number of drugs taken, but also to determine the final impact of these changes on clinical outcomes. |
| **De Souto Barreto 2016,** France  RCT/non-RCT  *G. Imp. Strategies to support multi-disciplinary teams* | **Inclusion:** Residents available at Wave 2 follow-up.  **Profit status of NH:** NR  **Intervention:** 163 NHs (total), 1822 residents. IQUARE (Impact d’une hospital QUAlité sur l’évolution des pratiques et le déclin fonctionnel des Résidents en EHPAD): Audit and feedback intervention on quality indicators associated to cooperative work meetings between hospital geriatricians and NH staff.  **Comparison:** NR, 2151 residents. Audit and feedback only. | **QoC/Health outcomes:** Benzodiazepine (BZD) use at the follow-up time-point; the use of long-acting BZDs at Wave two; new users of BZD (people who were not on BZD at Wave1 but who were on these drugs at Wave2); discontinuation in the use of BZD (people who were on BZD at Wave1 but who were no longer taking these drugs at Wave2). | - Compared to controls, the intervention group have had higher reductions in both BZD and long-acting BZD consumption, these differences were not significant. - An intervention aiming to improve NH quality indicators through education and support of NH staff did not reduce the consumption of both BZD and long-acting BZD among NH residents. |
| **El Haddad 2020,** France  Cohort  *G. Imp. Strategies to support multi-disciplinary teams* | **Inclusion:** Residents from the IQUARE study database who lived for ≥30 days in their institution and had data from two questionnaires (at baseline, at 18-months post-intervention).  **Profit status of NH:** Mix of for profit and not for profit.  **Intervention**: 85 homes, 1683 residents. IQUARE to improve polypharmacy: strong intervention involved an audit and feedback providing descriptive statistics to each NH regarding its own structure, resident health status, and quality indicators and the same descriptive statistics for the health administration subarea and the region; NHs had their quality report critically discussed during cooperative work meetings with a volunteer public hospital geriatrician from their health administration subarea and NH leadership staff, with the weaknesses and strengths of the QoC that each NH provided were identified, and strategies for overcoming weaknesses were developed.  **Comparison:** 90 homes, 2026 residents. Light intervention group received the audit and feedback intervention only, but interpreted these data without external help. | **QoC/health outcomes: n**umber of medication variation over time: total number of medications, psychotrops, anti-psychotics, anti-depressants, analgesics, opioids, paracetamol megapolypharmacy rate. | - No additional benefits for decreasing the number of medications and polypharmacy in NH residents compared to audit and feedback only. - There was unchanged quantity of prescription, there was a reduction in the number of psychotropic drugs in both study groups. |
| **Guion 2018,** France  RCT/non-RCT  *G. Imp. Strategies to support multi-disciplinary teams* | **Inclusion:** Residents of French NHs in subregions of health, with presence of a hospital with a geriatric department.  **Profit status of NH:** NR  **Intervention**: 85 homes, 1797 residents. IQUARE as described in El Haddad 2020, with a focus on impact on pain. Hospital geriatricians spent 2 to 6 half-days in each NH to provide the individual support.  **Comparison:** 90 homes, 2133 residents. Light intervention (see El Haddad 2020). | **QoC/health outcomes:** patients with pain complaint; failure estimates of the gold standard pain management; number of patients receiving gold standard pain management; number of patients receiving other pain management | - Results support nonspecific, collaborative, educational, and organizational interventions in NHs to decrease residents’ pain complaint and improve pain management. |
| **Laffon De Mazières 2019,** France  RCT/non-RCT  *G. Imp. Strategies to support multi-disciplinary teams* | **Inclusion:** All residents with ≥1 inappropriate prescription of antipsychotics at baseline who were still in residence at 18-months post intervention.  **Profit status of NH:** Mix of for profit and not for profit.  **Intervention**: 175 nursing homes, 464 residents. IQUARE to reduce potentially inappropriate prescription of antipsychotics: quality assurance and feedback audit (same as the control group), plus a geriatrician working in a department at the closest public hospital organized ≥5 collaborative work meetings (with NH nurse, coordinating physician, NH leadership/director) to support the implementation of action based on the audit results.  **Comparison:** 175 nursing homes, 555 residents. Quality assurance audit and feedback on baseline data. Each NH received NH-specific descriptive statistics, resident clinical data, quality indicator data and the same descriptive statistical analyses for all NHs in the regional health authority. | **QoC/health outcomes:** antipsychotic drug prescribing rates | - Collaborative work meetings with a geriatrician does not provide significant added value to a global quality assurance approach towards potentially inappropriate prescription of antipsychotics. - Individual feedback to each nursing home appears to have a substantial impact on decreasing potentially inappropriate prescription of antipsychotics. |
| **Rolland 2016,** France  RCT/non-RCT  *G. Imp. Strategies to support multi-disciplinary teams* | **Inclusion:** Randomly selected residents who resided in the NH for ≥30 days, who did not have a refusal to participate by their GP.  **Profit status of NH:** Mix of for profit and not for profit.  **Intervention**: 85 homes, 3017 residents. Quality improvement initiative on nursing practices and functional decline in NH Residents (IQUARE): an audit and feedback intervention, followed by cooperative work meetings with a volunteer public hospital geriatrician from their health administration subarea and NH leadership staff (coordinating physician, coordinating nurse, NH director) to develop strategies for improvement.  **Comparison:** 90 homes, 3258 residents. Received the audit and feedback intervention only, but interpreted these data without external help. | **QoC/health outcomes:** rate of ED transfers; ADL score, physical function quality indicators (e.g., dementia residents without formal diagnosis of dementia); systematic tracking of falls; annual review. | - The intervention had a significant positive effect on the prevalence of assessment of pressure ulcer risk, depression, pain, and prevalence of ED transfers. - It had no significant effect on functional decline. |
| **Overall care** | | | |
| **D’Arcy 2013,** USA  Cohort  *E. Preventing acute care hospital admissions or readmissions* | **Inclusion:** Fee-for-service Medicare beneficiaries ≥66 years diagnosed with ≥1 geriatric conditions; ≥3 consecutive months with ≥1 NH Carrier claim & no skilled nursing facility claims for ≥1 of those months.  **Profit status of NH:** NR  **Intervention**: NR homes, 2477 residents. Residents treated by a geriatrician. Each physician with ≥2 visits coded as geriatric medicine in one year was considered to be a geriatrician for all office, home and/or NH visits/consultations in that year.  **Comparison:** NR homes, 64074 residents. Residents treated by other physicians | **QoC/health outcomes:** Any ED use | - Geriatric care was associated with estimated annual decreases of 133 ED visits/1000 NH residents - Geriatric consultative care in collaboration with primary care providers may be as effective in reducing ED use as geriatric primary care - Could allow the existing supply of geriatricians to reach a larger number of patients. |
| **Gloth 2011,** USA  Cohort  *E. Preventing acute care hospital admissions or readmissions* | **Inclusion:** Nursing home residents.  **Profit status of NH:** For profit.  **Intervention**: 1 home, 390 residents. Dedicated post-acute care hospitalist (PACH) in the NH: a single physician (geriatrician) hired independently to provide the bulk of care for the residents in the facility. The physician came to the facility ≥3 days per week and attended on ≥70% of the residents in that NH throughout the study period.  **Comparison:** 1 home, 364 residents. Traditional model with a cadre of community physicians who provided care for the residents in the nursing facility.  Both facilities had a NP hired to oversee all residents as needed for the first 6 months of the evaluation, before instituting the PACH model. Only the PACH facility retained a NP for the second phase of evaluation. | **QoC/health outcomes:** unplanned discharges; falls; medication errors | - Significant increase in laboratory costs and no improvement in fall rates with intervention. - A nonsignificant reduction in medication errors and pharmacy costs. - Results support the hypothesis that a PACH model may lead to greater clinician involvement, which may be associated with an increase in clinical testing and a decrease in pharmacy costs and medication errors. If true, the latter would likely far offset any costs associated with additional laboratory testing. |
| **Rolland 2020,** France  RCT/non-RCT  *E. Preventing acute care hospital admissions or readmissions* | **Inclusion:** Residents ≥60 years, without diagnosed or documented dementia, not bedridden, living in the NH for ≥1 month, with a life expectancy of ≥1 year.  **Profit status of NH:** Mix of for profit and not for profit.  **Intervention**: 32 homes, 599 residents. Impact of Systematic Tracking of Dementia Cases on the Rate of Hospitalization in Emergency Care Units (IDEM): two multidisciplinary team meetings aimed to identify residents with dementia and to discuss an appropriate care plan; led by a coordinating physician who is a geriatrician; each resident was thus discussed twice at an interval of approximately 1 year.  **Comparison:** 32 homes, 829 residents. Usual practice. | **QoC/health outcomes:** transfer to the ED (proportion of residents with at least one ED transfer); incidence rate of ED transfers during the 12 months and 18 months of follow-up for 100 person-years); inappropriate hospitalizations (residents with ≥1 hospital admission judged as inappropriate) | - The findings do not support implementation of multidisciplinary team meetings for systematic dementia screening of all NH residents, beyond the national recommendations for dementia diagnosis, to reduce ED transfers. |

### Table B: Models for primary care within Long-Term Care

| **Author Year**  **Study design**  **Country** | **Participants, Intervention, Comparison, Outcomes (PICO)** | **Outcomes** | **Main conclusions** |
| --- | --- | --- | --- |
| **overall care** | | | |
| **Arendts 2018,** Australia  RCT/non-RCT  *E. Preventing acute care hospital admissions or readmissions* | **Inclusion:** Permanent (non-respite) resident of the RACF, aged ≥65 years, with a life expectancy of >180 days.  **Profit status of NH:** NR  **Intervention**: 3 homes, 101 residents. Assigned to NP that worked with general practitioners in a collaborative arrangement; NP used a best practice resource folder for the care processes being delivered and coordinated as part of the trial; NPs had an autonomous scope of practice that included independent diagnosis and prescribing, but conferred with the primary care physician as needed.  **Comparison:** 3 homes, 99 residents. Usual care: residents assigned to GPs who were responsible for their care. | **QoL:** Health Utilities Index Mark 2/3; EuroQol (EQ-5D-3L)  **QoC/health outcomes:** unplanned transfer to a hospital via the ED; death; hospital inpatient admissions; total hospital bed-days. | - Nurse practitioner care coordination resulted in no statistically significant change in rates of ED transfer or health care utilization, but better maintained resident quality of life. |
| **Craswell 2020,** Australia  Cohort  *E. Preventing acute care hospital admissions or readmissions* | **Inclusion:** Residents aged ≥70 years residing in aged care facilities who were transferred to the ED within study periods.  **Profit status of NH:** NR  **Intervention**: 1 home, 325 residents. Enhanced primary care provided by a NP candidate who provided triage, assessment, preliminary diagnosis and primary care for acutely unwell or deteriorating residents to reduce potentially preventable transfer to hospital; the NP candidate identified or was referred to acutely unwell residents, providing early intervention via advanced assessment; NP candidate collaborated with the GP in the care of residents, promoted ACP, and provided opportunistic education to staff.  **Comparison:** 1056 residents. Standard care. | **QoC/health outcomes:** transfer to ED; major diagnoses (reason for ED transfer); admitted to hospital; hospital length of stay; all cause re-presentation to ED; same cause re-presentation to ED | - Introduction of a NP candidate working with GPs improved most outcomes for residents transferred to hospital and increased documentation of advance care planning. |
| **Kobewka 2020,** Canada  Cohort  *E. Preventing acute care hospital admissions or readmissions* | **Inclusion:** Residents of LTCH  **Profit status of NH:** Mix of for profit and not for profit.  **Intervention**: 52 homes, 5617 residents. Same-day physician access in the LTC home when there is an acute change in resident health status  **Comparison:** 109 homes, 15007 residents. Physician visits the next day or later | **QoC/health outcomes:** ED visits; hospitalizations | - Residents of LTC homes with same-day physician access experience lower hospitalization and ED visit rates than residents in homes that wait longer for physicians, even after adjusting for important resident and home characteristics. |
| **Lacny 2016,** Canada  CBA/ITS  *E. Preventing acute care hospital admissions or readmissions* | **Inclusion:** Residents ≥65 years; had to have been alive for at least some of the time in each time period (before and after the intervention).  **Profit status of NH:** Not for profit.  **Intervention**: 1 homes, 45 residents. Nurse Practitioner-Family Physician (NP-FP) model of care: NP working in a collaborative practice agreement with 3 house physicians; practiced independently and reported directly to the Director of Nursing; collaborating with FPs during jointly conducted weekly rounds and consulting on an as-needed basis; participated in most interdisciplinary care meetings and communicated on an ongoing basis with families.  **Comparison:** 2 homes, 135 residents. Internal control: the NP, pharmacist, nursing staff and sometimes the FPs participated in joint medication review meetings for all residents. External control: medication review meetings were also held in the external control site with the pharmacist, nursing staff and sometimes FPs. | **QoC/health outcomes:** Emergency department transfer rates | - A NP-FP model of care resulted in smaller increases ED transfers per person-month between the before and after time periods. - Compared with the external control group, the NP-FP model resulted in a slightly larger increase in ED transfers per person-month. - Unable to make a definitive conclusion regarding the cost-effectiveness of the nurse practitioner-family physician model. |
| **Weatherall 2019,** Denmark  Cohort  *E. Preventing acute care hospital admissions or readmissions* | **Inclusion:** Nursing home residents who were ≥65 years.  **Profit status of NH:** NR  **Intervention**: 7 homes, 339 residents. A GP was assigned to be the dedicated GP for that home. Residents were encouraged, but not required, to select the dedicated GP.  **Comparison:** 783 homes, 26446 residents. No dedicated GP in NHs. | **QoC/health outcomes:** all hospitalizations; preventable hospitalizations; hospital readmission | - Assigning a dedicated GP was associated with a reduction in the probability a resident experienced a preventable hospitalization (26%) or a readmission (25%). - No significant effect on overall admissions. |
| **pain management** | | | |
| **Kaasalainen 2016,** Canada  CBA/ITS  *F. Specific condition/ intervention* | **Inclusion:** LTC residents.  **Profit status of NH:** Mix of for profit and not for profit.  **Intervention**: 4 (2 full and 2 partial intervention), 139 residents (full), 108 residents (partial). Full intervention: NPs led educational initiatives on pain management with their interprofessional teams (onsite pharmacists, physicians, licensed nurses, personal support workers, social workers, physiotherapists) and facilitated the implementation of evidence-based pain assessment tools and protocols; NPs participated in organizational-level interventions. Partial intervention: NP, but no pain management team; NP engaged in normal activities, and was contacted by the LTC home on a consultative basis without the added support of an IP pain management team.  **Comparison:** 2 homes, 98 residents. No NP, no pain management team. | **QoC/health outcomes:** pain; functional status; depression; agitation; clinical practice behaviours (e.g., documentation of pain assessments); quality of pain medication prescribing practices/Pain Management Index (PMI) scores | - Implementing a nurse practitioner-led pain team can significantly improve resident pain and functional status as well as clinical practice behaviours of LTC staff. - LTC homes should employ a NP, ideally located onsite as opposed to an offsite consultative role, to enhance inter-professional collaboration and facilitate more consistent and timely access to pain management. |
| **palliative care** | | | |
| **Temkin-Greener 2017; Temkin-Green 2018,** USA  RCT/non-RCT  *G. Imp. Strategies to support multi-disciplinary teams* | **Inclusion:** NH residents.  **Profit status of NH:** Mix of for profit and not for profit.  **Intervention:** 14 NHs, 2852 decedent residents. Improving Palliative Care Through Teamwork” (IMPACTT): a facility-level intervention involving a multi-component strategy that included implementing facility-based palliative care teams and providing staff with palliative and end-of-life geriatric training.  **Comparison:** 11 NHs; 609 non-randomized facilities, 2978 decedents; 119,486 decedents. No intervention. | **QoC/health outcomes:** Death in a hospital; Self-reported moderate to severe pain; Depressive symptoms; Number of hospital stays in the last 90 days  **Staff outcomes:** team communication; team cohesion; perceived palliative care competency; palliative care education; organizational readiness; staff satisfaction | - Did not demonstrate a significant impact of the intervention on residents’ risk-adjusted outcomes, when treating the findings in the traditional RCT framework. - As homes vary in their ability to adopt new care practices, and in their capacity to sustain them, reforms to create the environment in which effective palliative care can become broadly implemented are needed. |

### Table C: Models for direct patient care

| **Author Year**  **Study design**  **Country** | **Participants, Intervention, Comparison, Outcomes (PICO)** | **Outcomes** | **Main conclusions** |
| --- | --- | --- | --- |
| **depression** | | | |
| **Verkaik 2011,** The Netherlands  RCT/non-RCT  *F. Specific conditions/ interventions* | **Inclusion:** Residents of the participating NHs with dementia and co-morbid depression.  **Profit status of NH:** NR  **Intervention**: 9 homes, 62 residents. Introduction of the nursing guideline on depression in addition to usual care; training was provided by a trainers of the Centre for Training and Expertise and focused on CNAs; nursing team manager and activity therapist were invited to attend training sessions; “promotion group” consisting of the nursing team manager, activity therapist and two CNAs was installed, with a view to encouraging and supporting the team in following the guideline.  **Comparison:** 9 homes, 35 residents. Usual care. | **QoC/health outcomes:** depression severity (Minimum Data Set/Resident Assessment Instrument-Depression Rating Scale-Dutch version and Cornell Scale for Depression in Dementia-Dutch version); observed mood during morning care and residence in the living room | - Statistically significant reductions in depression severity can be obtained with the introduction of the nursing guideline on comorbid depression in dementia on psychogeriatric nursing home wards. - Effects could probably be enlarged if non-CNAs and nursing helpers are also trained, and managers pay more attention to the necessary conditions for successful introduction. |
| **overall care** | | | |
| **Boorsma 2011,** The Netherlands  RCT/non-RCT | **Inclusion:** Residents from residential care facilities.  **Profit status of NH:** Not for profit (primarily)  **Intervention**: 5 homes, 201 residents. Multidisciplinary integrated care, targeted to nurse assistants, but also involved family physician, geriatrician or psychologist; focused on identification and monitoring of the functional disabilities caused by chronic diseases; 3 basic elements correspond to those of the disease management model.  **Comparison:** 5 homes, 139 residents. Family physician was responsible for medical care and offered it on request; neither coordination nor structured planning of care; multidisciplinary meetings were mostly not attended by the family physicians. | **QoL:** Rand Health Insurance Study questionnaire  **QoC/health outcomes:** 32 risk-adjusted quality-of-care indicators (e.g., urinary tract infection, use of antipsychotic agents, decline in cognitive ability, increase in number of falls); mortality; admissions to hospital; activities of daily living | - Multidisciplinary integrated care resulted in improved quality of care for elderly people in residential care facilities compared with usual care. |
| **Boyd 2014,** New Zealand  RCT/non-RCT  *E. Preventing acute care hospital admissions or readmissions* | **Inclusion**: Residents of aged care facilities.  **Profit status of NH**: NR  **Intervention**: 29 homes, 1425 residents. Residential Aged Care Integration Program (RACIP): supports residential aged care staff and includes onsite support, education, clinical coaching, and care coordination provided by GNSs through on-site visits every other month and delivery of standardized gerontology education sessions for RAC nurses and care assistants. The RN Care Guides: quick evidence-based reference for common geriatric problems and to provide guidance about when to seek medical or advanced nursing consultation; education sessions specifically targeted at staff were held every 3 months at a central location; sessions facilitated staff peer support across aged care facilities; access to a wound care clinical nurse specialist.  **Comparison**: 25 homes, 1128 residents. The RN Care Guides (as in intervention group). | **QoC/health outcomes**: all resident hospitalizations and subgroups classified as medical or surgical admissions | - RACIP provided an efficient means of integrating advanced gerontology nursing expertise to support registered nursing and care assistant staff across several facilities. - Improved staff skill and knowledge may have translated into a significant difference in hospitalization rates for intervention facility residents. - Results may also indicate that the RACIP program had more of an effect on hospital admissions that were potentially avoidable (medical admissions), such as congestive heart failure, than for surgical admissions such as fractures, upon which staff may not have any preventive effect. |
| **Connolly 2015,** New Zealand  RCT/non-RCT  *E. Preventing acute care hospital admissions or readmissions* | **Inclusion:** All residents of selected facilities in 4 levels of RAC: rest-home care, private hospital care, dementia care, psychogeriatric care.  **Profit status of NH:** Mix of for profit and not for profit.  **Intervention**: 18 homes, 1123 residents. Aged Residential Care Healthcare Utilization Study (ARCHUS); comprised GNS-led staff education, facility bench-marking, GNS resident review, and multidisciplinary (geriatrician, primary-care physician, pharmacist, GNS, and facility nurse) discussion of residents selected using standard criteria, education and clinical coaching for nurses and caregivers.  **Comparison:** 18 homes, 875 residents. Usual supports and services from District Health Boards. | **QoC/health outcomes:** Acute ambulatory sensitive hospitalizations admissions; all acute admissions; all deaths; acute hospital bed-days | - There is no evidence that a multidisciplinary, non-disease-specific intervention into RAC results in overall reduction in acute hospitalizations, mortality, or hospital bed-days. - There remain unanswered questions over type and duration of interventions. |
| **Connolly 2016,** New Zealand  RCT/non-RCT  *E. Preventing acute care hospital admissions or readmissions* | **Inclusion:** All facility residents from LTC facilities with above average rates of potentially avoidable hospitalization in Auckland.  **Profit status of NH:** NR  **Intervention:** 18 homes, NR residents. ARCHUS intervention (see Connolly 2015).  **Comparison:** 18 home with NR residents. See Connolly 2015. | **QoC/health outcomes:** Time to first ‘big five’ (ischaemic heart disease, cardiac failure, stroke, COPD and pneumonia) admission; time to death; admissions for ‘big five’ conditions; non-‘big five’ acute admissions; all deaths | - A complex, multicomponent intervention may reduce acute hospitalizations for the conditions of ischaemic heart disease, cardiac failure, stroke, COPD and pneumonia. |
| **Connolly 2018,** New Zealand  CBA/ITS  *E. Preventing acute care hospital admissions or readmissions* | **Inclusion:** All residents from selected facilities with above-average rates of hospital presentations during the three-month period excluding the calendar month prior to intervention start were selected for inclusion.  **Profit status of NH:** Mix of for profit and not for profit.  **Intervention:** 21 homes, 1258 residents. Aged Residential Care Intervention Project (ARCHIP): team including facility senior nurse, resident’s general practitioner, GNS, geriatrician, and pharmacist; baseline facility assessment to identify areas of need and facility care plan developed by the interdisciplinary team; monitoring and benchmarking of resident indicators linked to quality of care provided; team meetings, including medication review by health providers; gerontology education and clinical coaching for nurses and care-givers.  **Comparison:** 42 homes, 1934 residents. Usual district health board support (no elements of the intervention group). | **QoC/health outcomes:** ED visits; ED visit due to congestive heart failure; chronic obstructive pulmonary disease; ischaemic heart disease, stroke and pneumonia, mortality | - A gerontology nurse specialist-led multidisciplinary team outreach intervention, targeted at selected conditions, decreases avoidable ED admissions of high-risk residents from selected facilities. |
| **Conway 2015,** Australia  CBA/ITS  *E. Preventing acute care hospital admissions or readmissions* | **Inclusion:** Residents over the age of 75 of the Residential Aged Care Facilities.  **Profit status of NH:** NR  **Intervention:** 4 homes, NR residents. Nurse-led telephone support service to RACFs for ED transfers; Aged Care Emergency Advanced Practice Nurse (ACE APN) with experience in the ED and care of older people; to support and improve emergency care for residents of RACFs through guidance, direction and support to clinical staff in RACFs, including telephone triage when transfer of an older person to ED was considered; clinical decision making processes of the ACE APN and RACF staff were guided by evidence based clinical guidelines adapted for RACFs in consultation with nurses from RACFs, the health service staff and Primary Care Medical Providers.  **Comparison:** 8 homes, NR residents. Usual care in RACFs. | **QoC/health outcomes:** ED presentations; ED admissions; total inpatient days; length of hospital inpatient stay; ED length of stay  **Staff:** staff confidence | - Reduction in calls to the service and transfers to ED while maintaining the role of the Primary Care Medical Provider in supporting effective management residents in RACFs. - Shared concerns among residents, families, RACF staff and ED staff about the extent to which an ED presentation is desirable for residents and this study has led to enhanced understanding of the extent to which contextual factors moderate the impact of ED telephone support, guidelines and education provided to RACF staff. |
| **Hullick 2016,** Australia  CBA/ITS  *E. Preventing acute care hospital admissions or readmissions* | **Inclusion:** Residents of aged care facilities.  **Profit status of NH:** Mix of for profit and not for profit.  **Intervention**: 4 homes, 453 residents. The Aged Care Emergency Service (ACE) service model of care comprised of an ED advanced practice nurse with aged care skills who led and coordinated the service, clinical care manual to support care, a nurse led telephone triage line, education, establishing goals of care prior to ED transfer, case management when in the ED, and development of collaborative relationships between stakeholders (i.e., residential aged care facilities, ambulance, Eds, GPs and the primary care organization).  **Comparison:** 8 homes, 836 residents. Usual care. | **QoC/health outcomes:** ED presentations; length of stay; hospital admission; 28-day readmission | - No overall reduction in ED presentations with ACE. - When compared to the controls, the intervention group reduced their ED length of stay by 45 min, and was 40 % less likely to be admitted to hospital. - A complex multi-strategy intervention coordinated by nursing staff can successfully reduce hospital admissions those living in RACF. |
| **Kulakçi 2013,** Turkey  CBA/ITS | **Inclusion:** Older adults who did not have any severe sensory or perceptual impairments that could create communication problems and who could perform their ADLs independently.  **Profit status of NH:** NR  **Intervention**: 1 home, 30 residents. Tailored individualized nursing care: performed in accordance with the Omaha System with someone who specializes in geriatric nursing. The Omaha System is a standardized framework for classifying nursing care elements. It incorporates client data and direct care delivery and uses a multidisciplinary approach.  **Comparison:** quasi-experimental design with repeated measures in a single group. | **QoL:** Self-Efficacy Scale; Healthy Life-Style Behaviours Scale II | - Tailored individualized nursing care services performed in accordance with individual requirements increase the self-efficacy perceptions and healthy lifestyle behaviors of older adults living in a NH. |
| **Rantz 2018**  Cohort  USA | **Inclusion:** Nursing home residents.  **Profit status of NH:** Mix of for profit and not for profit.  **Intervention**: 16 homes, NR residents. Missouri Quality Initiative (MOQI): advanced practice RNs working full-time in NHs; focused on quality improvement strategies with potential to influence healthcare outcomes.  **Comparison:** 27 homes, NR residents. Matched NHs from facilities in the same counties as the intervention homes, similar baseline quality measure scores, size, and ownership. | **QoC/health outcomes:** falls; pressure ulcers; urinary tract infections; indwelling catheters; restraint use; activities of daily living; weight loss; antipsychotic medication use | - Full-time advanced practice RNs in NHs can positively influence QoC - Positive impact on quality measure outcomes for the majority of the MOQI NHs, indicating budgeting for advanced practice RN services can be a successful strategy. |

### Table D: Allied health care teams

| **Author Year**  **Study design**  **Country** | **Participants, Intervention, Comparison, Outcomes (PICO)** | **Outcomes** | **Main conclusions** |
| --- | --- | --- | --- |
| **Activity involvement** | | | |
| **Wenborn 2013,** UK  RCT/non-RCT | **Inclusion:** Residents who meet the DSM-IV criteria for dementia and score less than 25 on the Mini Mental Status Examination.  **Profit status of NH:** NR  **Intervention**: 8 care homes, 104 residents. Occupational therapy intervention consisting of: assessment of the care home physical environment and an education programme aimed at enhancing staff knowledge, attitude and skill.  **Comparison**: 8 care homes, 106 residents. Usual care with no limitation on training or introducing new activity provision and were offered an abbreviated intervention once data collection was complete. | **QoL:** Quality of Life in Alzheimer’s Disease - Patient and Caregiver Report (QOL-AD)  **QoC/health outcomes:** dependency, challenging behaviour, depression, anxiety, severity of dementia, number and type of medication | - Demonstrated that it is feasible to provide a training and coaching intervention with staff in care homes. - Despite positive feedback from staff, overall there was no evidence to suggest that the intervention group improved relative to the controls. - Staff-rated quality of life for people with dementia decreased more within the intervention homes than the control homes. |
| **dementia care, including agitation** | | | |
| **Moyle 2014(A);  Moyle 2014,** Australia  RCT/non-RCT  *F. Focus on specific conditions/ interventions* | **Inclusion:** Residents with moderate to severe dementia and a history of agitated behaviour according to the Pittsburgh Agitation Scale.  **Profit status of NH:** NR  **Intervention:** 5 LTC facilities, 26 residents. Intervention assistants were trained massage therapists who provided participants with a standardised 5-min massage on each foot. Light pressure massage with long, gliding, rhythmical strokes and flexion, extension and rotation of the toes, foot and ankle was used. Unscented Sorbolene was applied as a lubricant for the massage.  **Comparison:** 5 LTC facilities (same as intervention), 29 residents. Trained assistants sat quietly near the participant’s feet for 10 min without talking or making physical contact with the resident. The quiet presence assistants were instructed that no deliberate touching or conversation was to occur during this time. | **QoL:** Cohen-Mansfield Agitation Inventory (CMAI); Observed Emotion Rating Scale (OERS)  **QoC:** physiological stress response (i.e., blood pressure [systolic and diastolic] and heart rate) | - Foot massage did not significantly reduce physiological stress, reduce agitation or improve mood when compared with the control group. - Instead agitation (measured by CMAI) increased in both the intervention and the control groups whilst mood (measured by OERS) was unchanged during the trial period. - Both foot massage and quiet presence demonstrated a significant reduction in post-intervention blood pressure scores in comparison to pre-intervention scores. - The close presence of another person may in fact reduce anxiety and promote comfort, thereby improving BP and HR measures. |
| **Rodríguez-Mansilla 2013,** Spain  RCT/non-RCT | **Inclusion:** Residents diagnosed with dementia according to the Diagnostic and Statistical Manual of Mental Disorders (DMS) VI criteria at least one year before the commencement of the study.  **Profit status of NH:** NR  **Intervention:** 3 'CARE' elderly care residential homes, Ear acupuncture: 40 residents; Massage therapy: 40 residents. Acupuncture: use of Shenmen (TF 4), 159.C Myorelaxant (the 159 Muscle relaxant located in the peripheral inferior concha, close to the spleen and liver Chinese points), and Xin (Co 15), with adhesive herbal seeds of Wangbuliuxing. Massage: every day from Monday to Friday. The massage was applied in the back and lower limbs for 20 min. The massage techniques used were superficial effleurage and deep kneading with moisturizing cream.  **Comparison**: N/A, 40 residents. No experimental treatment. | **QoC/health outcomes:** behaviour alterations, sleep disturbances, participation in therapy and in eating | - Massage therapy and ear acupuncture can improve behavior and sleep disturbances, and increase the participation in eating and rehabilitation organized in residential homes, in dementia patients. |
| **depression** | | | |
| **Travers 2017,** Australia  RCT/non-RCT | **Inclusion:** Residents with mild to moderate dementia (Mini-Mental State Examination score (sMMSE) 10), and evidence of symptoms of depression (12-item Geriatric Depression Scale (GDS-12R) (scores 4 indicate the presence of such symptoms).  **Profit status of NH:** Mix of for profit and not for profit.  **Intervention:** 4 NHs total, 10 residents. A Mental Health Therapist (MHT), worked individually with each resident participant to identify pleasant events, and develop an individually tailored plan to increase the availability and frequency of those events; training sessions focused on providing staff with an understanding of depression and dementia in nursing home residents, as well as practical ways to assist residents.  **Comparison:** 4 NHs total, 8 residents. A facility volunteer spent 30 min of one-to-one time, walking and talking with each resident each week. | **QoL:** depression (GDS-12R) and QOL (QOL-AD-NH)  **QoC:** Pleasant activities completed | - BE-ACTIV was well-accepted by both residents and staff and importantly, staff did not consider the intervention to be onerous. - Participants in the BE-ACTIV group demonstrated a significant increase in the number of pleasant activities they engaged in, however, statistically significant improvements on measures of depression and QOL were not found, perhaps due to small sample size. - Participants in the Walking and Talking group also showed significant improvement on a measure of QOL, but not depression. |
| **exercise/mobility** | | | |
| **Brett 2019,** Australia  RCT/non-RCT | **Inclusion:** Diagnosis of dementia, physically able to participate in physical exercise.  **Profit status of NH:** NR  **Intervention:** 2 nursing homes, (1) Intervention Group 1 = 20 residents; (2) Intervention Group 2 = 20 residents. (1) Intervention Group 1: physical exercise intervention for 45 min, once a week; (2) Intervention Group 2: physical exercise intervention for 15 min, three times a week.  **Comparison:** 2 nursing homes (same as intervention), 20 residents. Optional low intensity group activities running for 30–60 min, once or twice a day. All usual care activities were chair-based, such as bingo, observing concerts, watching movies, carpet bowls, quizzes, and gentle range of movement exercises. | **QoC/health outcomes:** reported fall incidents | - Demonstrated that physical exercise appears beneficial in helping manage physical decline. - Conclusions were cautiously drawn, including comparing the parameters of IG1 and IG2; both had a positive effect, but the majority of the results were not statistically significant. - Suggests that as little as 45 min of physical exercise per week could help manage or delay physical decline and prevent an increase in the number of reported falls for individuals living with dementia in nursing homes. |
| **Hewitt 2019,** Australia  RCT/non-RCT | **Inclusion:** Residents of RACF deemed able to participate safely in a group gym-based exercise programme.  **Profit status of NH:** NR  **Intervention:** 8 RACFs, 113 residents. Progressive resistance training (using HUR Health and Fitness Equipment), and high-level balance exercise. Sessions were one hour and conducted in small group settings, two days per week. The second stage was a maintenance programme conducted two days per week for 30 minutes.  **Comparison:** 8 RACFs, 108 residents. ‘Usual care’ continued without the programme. | **QoL:** QoL using the Short Form-36  **QoC/health outcomes:** Fall rate (falls per person year); Functional mobility measured using the short physical performance battery; Ambulance attendance at RACF; Ambulance transport to ER; Ambulance and ER visit; Admitted patients - no fracture; Admitted patients - fracture | - The SUNBEAM programme can be considered cost-effective in the context of other economic analyses performed alongside fall-prevention interventions. - The strongly significant reduction in fall rates found in the SUNBEAM trial has driven the incremental cost-effectiveness ratio calculations and resulted in the programme returning improved cost-effectiveness outcomes. |
| **Justine 2010,** Malaysia  Cohort | **Inclusion:** Residents living in a publicly funded shelter home who were able to walk 6 or more meters with or without any walking devices.  **Profit status of NH:** Not for profit  **Intervention:** 1 shelter home, 23 residents. The program conducted three times per week; Class consisted of warm-up, aerobic, resistance training, balance, stretching, and cool down exercises.  **Comparison: 1 shelter home (same as intervention), 20 residents.** A one-time health education talk, which covered topics such as the importance of: participating in regular physical activity and exercise, preventing falls, maintaining a balanced diet, and using correct posture during activities. | **QoL: Malay Version of the** Geriatric Depression Scale (GDS); life satisfaction or general well-being (Cantril’s Self-Anchoring Scale) | - After the 12-week intervention, both groups showed no significant changes in GDS score. - Improvement in life satisfaction was evidenced by an increase of 10.74% in Cantril’s Self-Anchoring Scale following the 12-week intervention, whereas the control group demonstrated a decrease of 11.26%. - However, the results did not reach statistical significance after the 12-week intervention for the exercise group. |
| **Lindelöf 2013,** Sweden  RCT/non-RCT | **Inclusion:** Residents dependent on assistance from a person in one or more personal ADL, a Mini Mental State Examination score of ≥10, approval of resident’s physician.  **Profit status of NH:** NR  **Intervention:** NR, 20 residents. High-Intensity Exercise Program (the HIFE Program): all exercises were performed in a functional weight-bearing position, for example, squats, walking over obstacles, reaching for objects while standing, and climbing stairs.  **Comparison:** NR, 28 residents. Activities performed while sitting e.g. watching films, singing, reading, and conversation. | **QoL:** cognitive functions (Mini Mental State Examination); morale (Philadelphia Geriatric Center Morale Scale); depressive symptoms (Geriatric Depression Scale)  **QoC/health outcomes:** balance capacity (Berg Balance Scale); gait ability | - Positive perceptions of participating in the high-intensity functional exercise program. - Compared with a social activity control group, more people in the exercise group perceived positive effects on tiredness, lower-limb strength, balance, and their feeling of safety and security when mobile. |
| **Telenius 2015(A); Telenius 2015,** Norway  RCT/non-RCT | **Inclusion:** Residents having dementia of mild or moderate degree as measured by the Clinical Dementia Rating scale (CDR 1 or 2).  **Profit status of NH:** NR  **Intervention:** 18 nursing homes, 87 residents. High Intensity Functional Exercises (HIFE)—program: exercise twice a week for 12 weeks (50-60 minutes per session); warm-up, strengthening exercises and balance exercises.  **Comparison:** 18 nursing homes (same as intervention),83 residents. Activities twice a week for 50-60 minutes; light physical activity, reading, playing games, listening to music and conversations. | **QoL:** QoL in late-stage dementia scale (QUALID); Clinical Dementia Rating Scale (CDR); Mini-Mental State Examination; Cornell Scale for Depression in Dementia  **QoC/health outcomes:** Activities of Daily Living; Cognition; Severity of behavioural and neuropsychiatric symptoms; depression; balance | - Demonstrates that people with mild and moderate dementia are capable to perform high intensity functional exercises and stay motivated to attend exercise over a period of time. - High intensity functional strength and balance exercises were effective to improve balance and strength and reduce apathy and agitation (trend) in the targeted population. - The differences between groups were significant for the agitation sub-score. |
| **foot care** | | | |
| **Wylie 2017,** Scotland  RCT/non-RCT | **Inclusion:** Residents who experienced one or more falls in the previous year, who have a foot problem within the scope of practice of a UK trained podiatrist.  **Profit status of NH:** NR  **Intervention:** 6 care homes, 23 residents. Core podiatry (as in control group); foot orthoses provision, footwear assessment and provision and a course of foot and ankle exercises.  Comparison: N/A, 20 residents. Core podiatry only (routine nail and callus maintenance). | **QoL:** EQ-5D  **QoC/health outcomes**: falls, falls self-efficacy, current foot problems, balance function, mobility, activities of daily living, ankle joint muscle strength | - Conducting an RCT of a podiatry intervention to reduce falls in care homes is feasible with regard to recruitment, retention, and intervention delivery. - The effectiveness of the intervention cannot be determined from our results (pilot study). - Data reported here support testing the intervention in a definitive full scale multicentre trial to test effectiveness. |
| **hearing care** | | | |
| **Hopper 2016**  RCT/non-RCT  Canada | **Inclusion:** Residents with Alzheimer’s disease, vascular dementia, or mixed dementia; had visual function sufficient to read 24- to 28- point font; mild-to-moderate hearing impairment.  **Profit status of NH**: NR  **Intervention**: 5 homes, 25 residents. Hearing ability measured by an audiologist according to pure-tone audiometry  **Comparison:** NA (participants received both hearing assessments). Hearing ability as recorded by LTC staff. | **QoC/health outcomes:** hearing ability | - Health care staff completing the assessments were able to recognize hearing loss even among residents with early- to middle-stage dementia, and that they were able to distinguish between cognitive-communication limitations caused by dementia and those caused by hearing loss - 11 of 25 participants (44%) had hearing loss that was either unidentified (n = 7) or underestimated (n = 4). |
| **hip fracture** | | | |
| **Beaupre 2020,** Canada  Cohort | **Inclusion:** Ambulatory nursing home residents who sustained a hip fracture.  **Profit status of NH:** NR  **Intervention:** NR, 46 residents. 30 sessions of rehabilitation more than 10 weeks in their nursing home after hospital discharge; Usual rehabilitation services that would normally be provided by the long-term-care facility were discontinued during this time.  **Comparison:** NR, 31 residents. Usual post-fracture care in their nursing home after hospital discharge. | **QoL:** quality-adjusted life years (QALYs), using the EQ5D  **QoC/health outcomes:** outpatient visits, physician claims, and inpatient readmissions | - A rehabilitation program (10 weeks) may be cost-saving from a health care payer perspective, primarily due to a reduction in post-fracture hospital readmissions. - Indicates a modest, but sustained mobility benefit. - Results should be considered preliminary, but support further work evaluating the impact and value of post-fracture rehabilitation for this group of patients. |
| **nutrition** | | | |
| **Beck 2016,** Denmark  RCT/non-RCT | **Inclusion:** Residents in NHs with Eating Validation Scheme (EVS) made by the nursing staff caregivers.  **Profit status of NH:** NR  Intervention: 2 NHs, 9 residents. Educated nutrition coordinator (as in control group); new model for multidisciplinary nutrition support; focus on individual treatment of the potentially modifiable nutritional risk factors.  **Comparison**: N/A, 22 residents. Education of the nutrition coordinator. | **QoL:** EuroQol-5D-3L  **QoC/health outcomes:** physical performance, nutritional status, oral care, fall incidents, hospitalization, mortality | - Multidisciplinary nutritional support focusing on individual treatment of potentially modifiable nutritional risk factors identified with the EVS, could have a positive effect on quality of life, muscle strength, and oral care. |
| **Lin 2010,** Taiwan  RCT/non-RCT  *F. Focus on specific condition/ intervention* | **Inclusion:** LTC facility residents.  **Profit status of NH:** NR  **Intervention**: 4 LTCs, 125 residents. Case management model, with a hospital-based, multidisciplinary care-team; a dietitian gave each resident their dietary suggestions.  **Comparison**: 4 LTCs, 249 residents. Usual care. | **QoC/health outcomes:** malnutrition (including hypoalbuminemia, hypocholesterolemia, low hemoglobin, underweight, waist circumference) | - Aggressive nutritional support by dietitians and other team members is essential to improve the quality of medical care for the elderly. - Results suggest that multidisciplinary team-care with a case management model, could be best used to care for the elderly living in long-term care facilities. |
| **Törmä 2015,** Sweden  Cohort | **Inclusion:** Participants were required to reside in a nursing home and to not have a terminal illness with an expected survival <6 months.  **Profit status of NH:** NR  **Intervention:** 2 NHs, 94 residents. Multifaceted intervention that included support, guidance, practice audits, and feedback.  **Comparison**: 2 NHs, 78 residents. Educational outreach visit (EOV) strategy: 3-hour lecture at one occasion regarding the operationalised nutritional guidelines. | **QoL:** Health-related quality of life (EQ-5D)  **QoC/health outcomes:** nutritional status, functional ability/activities of daily living, cognitive function, biochemical markers | - The use of two different strategies to implement nutritional guidelines in a NH setting did not result in any differences in nutritional status or physical function among the NH residents over an observation period of 1.5 years. - Despite a cautious interpretation, it was still observed that the external facilitator approach was associated with delayed cognitive deterioration in the NH residents who underwent cognitive testing before and after the intervention. |
| **optimal/appropriate medication use** | | | |
| **Balsom 2019,** Canada  RCT/non-RCT | **Inclusion:** Residents ≥65 years of age, residing on one floor of the LTC facility.  **Profit status of NH**: NR  **Intervention**: 1 home, 22 residents. Participants received an in-depth medication review, by pharmacy students, discussions with nursing care team, and involvement of physician, with focus on deprescribing unnecessary or potentially harmful medications.  **Comparison:** 1 home, 23 residents. Participants continued to have their medications reviewed and reordered by the physician on a quarterly basis and the pharmacist completed an annual medication review to assess for drug interactions, dose adjustments, lab monitoring and any modifications to therapy required (i.e. not specifically deprescribing-focused), in addition to pharmacist consultation services as required. | **QoL:** Resident Assessment Instrument (including the following scales: cognitive performance, depression, pain, social engagement, health status, and activities of daily living)  **QoC/health outcomes:** number of prescribed regular and PRN medication | - A pharmacist-led deprescribing intervention can reduce the number of unnecessary and potentially harmful medications taken by long term care residents. |
| **Dorfman 2020,** Canada  Cohort | **Inclusion:** Residents prescribed ≥2 medications with pharmacogenetic indications.  **Profit status of NH**: NR  **Intervention**: 4 homes, 90 residents. Clinical pharmacists reviewed the residents’ medications in the context of their pharmacogenetic profile to identify potential DTPs and provided written recommendations (Pharmacists’ Opinion letter) for therapy adjustments; letters were shared with the NH pharmacists and physicians who implemented therapy changes where warranted.  **Comparison:** 4 homes, 897 residents. Managed according to the current standard medication management program in the NHs. | **QoC/health outcomes:** Relative Fraction of all Dispensed Medication with PGx Indications; average medication load | - Supports the implementation of pharmacogenetic testing for residents on multiple medications and illustrates the potential of personalized medication optimization to improve the management of vulnerable patient populations. - Has the potential to reduce the utilization of medications, which pose a higher risk of adverse side effects, guide deprescribing of medications that have reduced clinical efficacy, and potentially improve the patients’ QoL. |
| **Hashimoto 2020,** Japan  RCT/non-RCT | **Inclusion:** Residents taking five or more medications.  **Profit status of NH**: Not for profit.  **Intervention**: 2 homes, 32 residents. One pharmacist (>10 years of experience) visited the NHs once a week; checked residents whose prescriptions had changed, physical condition had changed, and checked for adverse events; reviewed residents’ eating, excretory functions, sleep and motor status to identify potential issues and solutions; consulted with the physician; and shared information with resident staff who were responsible for undertaking follow-up.  **Comparison:** 2 homes, 36 residents. Usual care. | **QoL:** SF12 v2 Standard, Japanese Version 2.0  **QoC/health outcomes:** potentially inappropriate medication; falls; proportion of participants whose prescriptions were changed because of the pharmacist’s recommendation; activities of daily living; sleep status; adverse events | - Results suggested a trend toward fewer potentially inappropriate medication and falls in the intervention group. - There was no difference in ADL, QoL, and sleep scores. |
| **McDerby 2019,** Australia  RCT/non-RCT | **Inclusion:** Residential aged care home hospital of homes with high‐level care (e.g., advanced dementia) residents.  **Profit status of NH**: Not for profit.  **Intervention**: 1 home, 74 residents. A residential care pharmacist position was implemented at the study site; the pharmacist collaborated with the nursing and management teams, and their activities included medication reviews and quality improvement activities to optimize quality use of medicines for residents and medication administration practices for staff.  **Comparison:** 1 home, 43 residents. No residential care pharmacist, but received medication management reviews from an accredited pharmacist on a visitational basis. | **QoC/health outcomes:** number of: residents administered medication, residents administered medication with at least one dose form modification, modified dosage forms, inappropriately modified dosage forms; time spent per resident on medication rounds; medication incidents; monthly medication incident reporting rate; allergy and adverse drug reaction documentation rates | - Demonstrated the inclusion of a pharmacist in RAC homes can improve medication administration practices by reducing inappropriate dosage form modification and staff time spent on medication administration rounds, and increasing the documentation of resident allergies, adverse drug reactions and medication incidents. |
| **McDerby 2020,** Australia  RCT/non-RCT | **Inclusion:** Residents of aged care homes.  **Profit status of NH**: Not for profit.  **Intervention**: 1 homes, 58 residents. The residential care pharmacist was already performing medication management reviews at both residential aged care homes on a visitational basis. The pharmacist was employed part-time (0.4 full time equivalent, two consecutive days per week) for six months. The pharmacist collaborated with the care team and documented all their activities performed for the study.  **Comparison:** 1 home, 39 residents. Usual care. | **QoC/health outcomes:** polypharmacy; drug burden index; antipsychotic and benzodiazepine use; hospital admission rates and length of stay; ED presentation rates; inappropriate dosage form modification; allergies, adverse drug reaction  **Staff:** staff time spent on medication administration rounds | - A residential care pharmacist may positively influence indicators of medication use quality and improve medication administration practices by reducing inappropriate dosage form modification, staff time spent on medication administration rounds, and increasing the documentation of resident allergies, adverse drug reactions and medication incidents. |
| **Patterson 2010,** Northern Ireland  RCT/non-RCT | **Inclusion:** All nursing home residents aged ≥65.  **Profit status of NH**: Mix of for profit and not for profit.  **Intervention**: 11 homes, 173 residents. Fleetwood Northern Ireland model of pharmaceutical care: pharmacists visited each NH monthly; collected clinical background information from residents’ NH records and the GP practices and from local community pharmacists when discrepancies in records were identified; assessed the pharmaceutical care needs of each resident by interviewing the residents, their named nurses and their family members or caregivers. Potential and actual medication-related problems were identified, recommendations for intervention were recorded.  **Comparison:** 11 homes, 161 residents. Usual care. | **QoC/health outcomes:** the proportion of residents prescribed one or more inappropriate psychoactive medications; falls, change in the number of inappropriate psychoactive medications | - An adapted U.S. model of care targeting specific drugs could be successfully implemented in NHs in Northern Ireland, demonstrating an effect on inappropriate psychoactive medication. - Challenge will be to extend this model to other therapeutic areas to provide a holistic approach to pharmaceutical care in this setting. |
| **Sluggett 2020,** Australia  RCT/non-RCT | **Inclusion:** All permanent residents of RACFs taking ≥1 regular medication.  **Profit status of NH**: Not for profit.  **Intervention**: 4 homes, 99 residents. Simplification of Medications Prescribed to Long-Term care Residents (SIMPLER): an experienced clinical pharmacist applied the validated, 5-item Medication Regimen Simplification Guide to each resident to identify opportunities for medication simplification; prepared a report with recommendations for medication regimen simplification for the GP and senior nursing staff; discussed report with the nurse consultant and GP when possible; recommendations were implemented at the discretion of the residential services manager or senior RN at the RACF and the resident’s GP.  **Comparison:** 4 homes, 143 residents. Routine care. | **QoL:** Quality of Life in Alzheimer’s disease (QoL-AD) scale score  **QoC/health outcomes:** Number of medication administration times; Short Assessment of Patient Satisfaction scale score; rate of falls; rate of overnight hospitalizations; rate of mortality; rate of medication incidents | - Provides new evidence that one-off application of a new 5-step medication simplification intervention reduces the number of medication administration times for residents without negatively affecting resident quality of life, satisfaction, or other important health outcomes. - The effect size is modest but significant, and would release staff for other resident interactions or care activities for a meaningful period of time. |
| **oral health** | | | |
| **Barbe 2019,** Germany  RCT/non-RCT  *H. Imp. Strat. targeting specific conditions/ risk factors* | **Inclusion:** NH residents with ≥4 remaining teeth.  **Profit status of NH**: Not for profit.  **Intervention**: 1 home, 25 residents. Every two weeks, a dental nurse performed one brushing session and an oral examination; final examination was performed after 3 months; all NH staff received an in-house training session regarding oral hygiene recommendations for daily practice.  **Comparison:** 1 home (same NH as intervention group), 25 residents. Usual care: self‐brushing (manual toothbrush) or using interdental devices without supervision by staff, brushing by staff (manual toothbrush) or supervision of resident’s brushing (manual toothbrush) by staff; same initial training session as the intervention group; besides in‐house training of NH staff and pretreatment of professional dental cleaning session, no other parameters were changed regarding their oral hygiene practices from before study. | **QoC/health outcomes:** oral health indices (plaque index, gingivitis index, Quigley‐Hein index, papilla bleeding index, oral hygiene index, Volpe‐Manhold Index); food debris (vestibulum, upper prostheses, lower prostheses); number of teeth; number of lost teeth | - Regular professional brushing every 2 weeks by a dental nurse can be recommended for NH residents to improve oral health parameters and to help reduce root caries incidence as a basis to preserve the number of teeth. - Such oral hygiene procedures will maintain and improve the oral health of NH residents. |
| **Barbe 2020,** Germany  RCT/non-RCT  *H. Imp. Strat. targeting specific conditions/ risk factors* | **Inclusion:** NH residents at a senior’s residence.  **Profit status of NH**: Not for profit.  **Intervention**: 1 home, 40 residents. Professional brushing with a three headed brush by a dental nurse every three weeks for three months. NH staff administered the usual daily oral hygiene as before, and received in-house training regarding oral hygiene knowledge and practice guidelines.  **Comparison:** 1 home, 25 residents. Professional dental cleaning session by the dental nurse in the presence of the dentist in the NH received two-week brushing interval with a manual toothbrush. | **QoC/health outcomes:** oral health (e.g., plaque index, gingivitis index, root caries index); volpe-manhold index; cognition nutrition | - Professional brushing performed regularly by a dental nurse is an efficient method to improve oral hygiene in NH residents and may contribute to better nutritional status and QoL. - Brushing every three weeks with a three-headed brush was not effective at improving gingivitis or plaque and was inferior to the two-week brushing interval with a manual toothbrush. |
| **Marchini 2018,** USA  RCT/non-RCT  *H. Imp. Strat. targeting specific conditions/ risk factors* | **Inclusion:** Nursing facility residents.  **Profit status of NH**: Mix of for profit and not for profit.  **Intervention**: NR homes, 31 residents each in Group B and C. Group B: educational program: a one-hour dental hygienist-delivered interview with a sample of caregivers at each facility; a one-hour tailored lecture aiming to address the specific issues raised by the caregivers from each NF (e.g., working with combative residents, how to perform oral hygiene in a time-efficient manner) and hands-on training, followed by the hygienist visits to the NF every other week; at visits, oral hygiene instructions were reinforced and the hygienist brushed patients’ teeth. Group C: educational program as given to Group B, plus 1% chlorhexidine varnish monthly application.  **Comparison:** NR homes, 19 residents. Current oral hygiene practice. | **QoL:** SF-36  **QoC/health outcomes:** nutritional status; oral health (oral lesions, denture status, number of teeth, dental plaque index, denture plaque index, bleeding on brushing, gingival bleeding index, coronal DMFS, root DMFS, and self-reported dry mouth); oral health impact; geriatric oral health assessment index; microbiological outcomes | - There were no statistically or clinically significant differences among the intervention groups at 6 months for any of the recorded clinical or microbiological outcomes. |
| **Morino 2014,** Japan  RCT/non-RCT | **Inclusion:** NH residents >64 years old. Participants had to have ≥1 tooth, but they could have partial dentures.  **Profit status of NH**: NR  **Intervention**: 1 home, 17 residents. Received short-term professional oral health care after breakfast once per week for 1 month from two dental hygienists. They performed no dental scaling and no thorough cleaning, but simply brushed.  **Comparison:** 1 home, 17 residents. Normal oral hygiene procedures (self-care or care with help from nursing home staff) in the follow-up period (baseline to 5 months). The two groups differed only in who was delivering the oral care. | **QoC/health outcomes:** oral health (e.g., oral microbial parameters, dental plaque index); functional dependence | - The short-term professional oral health care can improve geriatric oral health. |
| **Nishiyama 2010,** Japan  RCT/non-RCT | **Inclusion:** Residents dependently living in long-term nursing care.  **Profit status of NH**: NR  **Intervention**: 1 home, 26 residents. All subjects who were able used the sink facilities in their room performed daily care 2–3 times a day after each meal; dental hygienists provided professional care for 20 min once per week (i.e. dental brushing of teeth surfaces, mucosal cleaning with a sponge brush, tongue cleaning with tongue brush, denture cleaning, and oral washing with tap water in addition to the daily oral care); mucosal cleaning was performed by sponge brush soaked in top water for 1 min.  **Comparison:** 1 home, 24 residents. Same oral care, but no additional mucosal cleaning. | **QoC/health outcomes:** Numbers of mutans streptococci in saliva; plaque and tongue samples; numbers of subjects which detected Candida species | - Demonstrates the beneficial effects of mucosal care on infection control of pathogens such as mutans streptococci and Candida during oral professional care - Oral professional care is limited by the high costs of odontologic assistance, the oral and general health status of the elderly, the lack of time available for caregivers to carry out the tasks, in the institutionalized elderly. |
| **Seleskog 2018,** Sweden  RCT/non-RCT | **Inclusion:** Nursing home residents.  **Profit status of NH**: NR  **Intervention**: 1 home, 17 residents. Study dental hygienists gave weekly support over the course of 3 months: (1) participation in staff meetings with the director of nursing and nursing staff at the beginning, after 6 weeks and at the end, (2) individualized and customized theoretical and hands-on guidance and support for each resident (1x week), (3) individualized and customized written oral hygiene prescriptions for each specific resident for special oral hygiene devices procedures or products.  **Comparison:** 1 home, 25 residents. The director was informed that she could contact the two study dental hygienists if needed. Otherwise, oral care was performed as usual. | **QoC/health outcomes:** oral health assessment (including: gum and lip assessments, dental plaque levels and gingival bleeding) | - Revised Oral Assessment Guide gums and lips scores showed a tendency to decrease in the intervention group, but remained high in the control group. - Plaque levels improved significantly after intervention, and a trend towards less gingival bleeding was observed. - Oral health care support in NHs may have to be organized in new ways with long-term regular presence and guidance by dental hygienists to the nursing staff. |
| **Sumi 2010,** Japan  RCT/non-RCT | **Inclusion:** Nursing home residents requiring care.  **Profit status of NH**: NR  **Intervention**: 1 home, 27 residents. A dentist provided oral care (3x week); professional oral care was given by the dentist using an oral care support instrument developed at the National Center for Geriatrics and Gerontology.  **Comparison:** 1 home (same as intervention), 26 residents. Usual care (oral cleaning was done following the oral care methods of the nursing home). | **QoC/health outcomes:** nutritional status (body weight, body mass index, serum albumin, and high-density lipoprotein cholesterol) | - The intervention of oral care alone can serve to maintain the nutritional status of older people who require care. |
| **Tynan 2018,** Australia  CBA/ITS | **Inclusion:** Residents of RACFs.  **Profit status of NH**: NR  **Intervention**: 4 (3 RACFs, one multi-purpose health services that include residential aged care beds), 111 residents. Integrated oral health program utilizing tele-dentistry and visiting Oral Health Therapists: incorporates a visiting oral health therapist for screening, education, and referral to a dentist for a remote real-time oral examination; oral health therapists can simultaneously communicate with a remotely located dentist; an appointment at the oral health facilities is made, if required.  **Comparison:** 5 (including 3 RACFs and 2 MPHs), 141 residents. No integrated oral health program. | **QoL:** Geriatric Oral Health Assessment Index | - Oral health therapists and tele-dentistry shows potential to improve the oral health outcomes of residents of RACFs. - Improvements for managing oral health of residents with high care needs were observed. - RACFs without easy access to an oral health service will also likely benefit from the increased support and training opportunities that the program enables. |
| **Wikström 2017,** Sweden  RCT/non-RCT  *H. Imp. Strat. targeting specific conditions/ risk factors* | **Inclusion:** Dependent elderly residents with ≥10 natural teeth and no removable dentures.  **Profit status of NH**: NR  **Intervention**: 1 homes, 33 residents. Professional oral care (1x week), by a dental hygienist; oral care was carried out in the residents’ private rooms; treatment included brushing of the teeth, at labial and lingual sides, with use of an electric toothbrush and a 1100 ppm sodium fluoride dentifrice; information and training in oral hygiene procedures were given; each participant as well as their main responsible nursing aid received an electric toothbrush as the same kind as was used by the two dental hygienists, and instructions and training on how to use the brush.  **Comparison:** 1 home, 35 residents. Usual care (the oral health care procedures followed the ordinary routines for the department). | **QoC/health outcomes:** number of prescription medicines; number of teeth; labial minor gland secretion; supragingival plaque score; tongue samples (total viable microbial count and proportions of streptococci, lactobacilli, F. nucleatum, P. intermedia/ nigrescens, P. gingivalis, C. albicans, S. aureus and enteric rods) | - Weekly professional oral hygiene care in dentate, dependent elderly residents resulted in a decreased plaque score and a decrease in bacteria associated with periodontal diseases. - No affect in the proportion of bacteria with acidogenic and aciduric potential, the frequency of micro-organisms associated with oral mucosal infections and aspiration pneumonia. - Decrease in the proportion of bacteria associated with good oral health. - Assisted oral hygiene care alone is not sufficient to regain an oral microbial flora associated with good oral health in dentate. |
| **Zenthöfer 2013,** Germany  RCT/non-RCT  *H. Imp. Strat. targeting specific conditions/ risk factors* | **Inclusion**: Residents at care level 1 or with no care level.  **Profit status of NH**: NR  **Intervention**: NR, No remotivation group = 26 residents; Dentist remotivation group = 27 residents; Staff remotivation group = 26 residents. Teeth and dentures were cleaned professionally and individual instruction was given. One of these groups was also re-instructed and remotivated by a dentist; one also received help from, and was remotivated by, staff educated in dental hygiene; third therapy group was not remotivated after professional cleaning of teeth and dentures.  **Comparison**: NR, 23 residents. Oral hygiene was performed in the usual way. | **QoC/health outcomes:** mean plaque, gingival bleeding, and denture hygiene indices | - All three types of intervention were clinically successful, and improved oral hygiene in comparison with the control group. - Comparison of the results from the three therapy groups with each other revealed small differences only. - Professional cleaning of teeth and dentures, with individual instruction, can be recommended to improve oral hygiene. - The effect decreases over time and renewal of the intervention is necessary. |
| **Zenthöfer 2016,** Germany  RCT/non-RCT  *H. Imp. Strat. targeting specific conditions/ risk factors* | **Inclusion:** Residents who had natural remaining teeth and/or dentures.  **Profit status of NH**: NR  **Intervention**: 8 NHs, 144 residents. Comprehensive education program for the carers and the implementation of ultrasound baths for denture cleaning  **Comparison**: 6 NHs, 75 residents. No intervention. | **QoC/health outcomes:** oral hygiene (Plaque Control Record (PCR), Gingival Bleeding Index (GBI), Community Periodontal Index of Treatment Needs (CPITN), Denture Hygiene Index (DHI)) | - Carers’ education improves oral health of care-dependent nursing home residents with and without dementia. - The implementation of ultrasound baths for denture cleaning is a simple but effective measure to improve denture hygiene. - Improved communication between carers, residents, relatives and health care professionals may be helpful and improves residents’ (dental) care quality. |
| **overall care: stroke-related disabilities** | | | |
| **Sackley 2016,** UK  RCT/non-RCT | **Inclusion:** Residents with a history of stroke or transient ischaemic attack.  **Profit status of NH:** Mix of for profit and not for profit.  **Intervention**: 114 care homes, 568 residents. OT package for residents targeted towards maintaining abilities in functional activity; in particular, personal activities of daily living (ADL) such as feeding, dressing, toileting, transferring and mobilizing; followed a patient-centred goal-setting approach.  **Comparison**: 114 care homes, 474 residents. Usual care. | **QoL:** European Quality of Life-5 Dimensions, three levels (EQ-5D-3L)  **QoC/health outcomes:** Activities of daily living, functional mobility, mood, adverse events | - We did not find evidence to suggest that a 3-month OT package designed for an older care home population with stroke-related disabilities is clinically beneficial, or that it provides a cost-effective use of resources. |
| **vision care** | | | |
| **Man 2020**  RCT/non-RCT  Australia | **Inclusion:** LTC residents (≥12 months stay); visually impaired with moderate cognitive functioning or better from in urban and rural Victoria, Australia.  **Profit status of NH**: NR  **Intervention**: NR homes, 95 residents. Residential ocular care (ROC) model: visually impaired residents underwent an on-site eye examination by trained optometrists and were referred for appropriate follow-up care.  **Comparison:** NR homes, 83 residents. Usual care, including referral to an external eyecare provider. | **QoL:** EuroQoL 5-Dimension Index  **QoC/health outcomes:** Distance Presenting visual acuity (PVA) in the better eye; near PVA in the better eye; Cornell Scale for Depression in Dementia; Impact of vision impairment-residential care reading, mobility, emotional; quality of vision frequency, severity, bother; number of: falls & injurious falls in past 9 months | - The residential ocular care model was effective in improving clinical visual outcomes, specifically near vision, as well as subjective quality of vision and emotional well-being for residents living in residential care facilities in Australia. |

### Table E: Models for preventing acute care hospital admission and readmission

| **Author Year**  **Study design**  **Country** | **Participants, Intervention, Comparison, Outcomes (PICO)** | **Outcomes** | **Main conclusions** |
| --- | --- | --- | --- |
| **Overall care** | | | |
| **Kane 2017,** USA  RCT/non-RCT | **Inclusion:** NHs needed to have the ability to safely manage acute changes in resident condition on-site, and availability of technical support for training and data submission; residents were Medicare beneficiaries with fee-for-service coverage.  **Profit status of NH:** Mix of for profit and not for profit.  **Intervention**: 33 homes, 9050 residents. Interventions to Reduce Acute Care Transfers (INTERACT): tools that help NH staff identify and evaluate acute changes in NH resident condition and document communication between physicians; care paths to avoid hospitalization when safe and feasible; and advance care planning and quality improvement tools.  **Comparison:** 52 homes, 14428 residents. A combination of patients receiving usual care with no contact and those receiving additional attention. | **QoC/health outcomes:** all hospitalizations; potentially avoidable hospitalizations; ED visits without hospital admission; 30-day readmission rate; all-cause admissions within 30 days of NH admission; all admissions >31 days after NH admission; mortality; composite outcome (NH deaths, hospitalizations, and observations stays) | - Training and support for INTERACT implementation as carried out in this study had no effect on hospitalization or ED visit rates in the overall population of residents in the participating NHs. - Results have several important implications for implementing quality improvement initiatives in NHs. |

### Table F: Models of care focused on specific conditions/interventions

| **Author Year**  **Study design**  **Country** | **Participants, Intervention, Comparison, Outcomes (PICO)** | **Outcomes** | **Main conclusions** |
| --- | --- | --- | --- |
| **Dementia care, including agitation** | | | |
| **Borbasi 2011,** Australia  Cohort | **Inclusion:** Staff caring for residents of RACFs that are experiencing cognitive deterioration as a result of dementia.  **Profit status of NH:** NR  **Intervention**: 7 homes, NR residents. DEMOS (dementia outreach service): a multidisciplinary healthcare team who work to assess and manage residents that are experiencing cognitive deterioration as a result of dementia; led by a NP who specializes in dementia care, assisted by a clinical nurse, endorsed enrolled nurse, assistant-in-nursing, clinical facilitator, social worker and administrative assistant; working with staff to coach them in how best to manage the referred resident’s behaviour; implement tailored interventions suited to the resident’s needs.  **Comparison:** 13 homes, NR residents. No DEMOS intervention. | **QoL:** amended version of the Dementia Quality of Life  **QoC/health outcomes:** presentations and admissions to the ED  **Staff:** Stress; dementia knowledge; level of self confidence | - Increases the knowledge and confidence of carers and their ability to manage residents with BPSD - Has the capacity to increase staff awareness of resident feelings and diminish levels of stress in carers faced with the aggressive behaviours associated with BPSD - Has positive effects on improving staff awareness of the various symptoms of dementia and how these are linked to residents’ behaviours - Leads to reductions in aggressive and difficult behaviours in residents with BPSD, and a reduction in the number of referrals to other services. |
| **Henskens 2017,** The Netherlands  RCT/non-RCT | **Inclusion:** NH residents ≥65 years of age with moderate to severe dementia living in a psychogeriatric ward for ≥3 weeks.  **Profit status of NH:** For profit.  **Intervention**: 1 home, 37 residents. Movement-oriented restorative care (MRC): derived from the concept of function focused care (FFC) and restorative care; focuses on the integration of physical activity in the daily lives of NH residents with dementia; includes nursing staff, department heads, physiotherapists, occupational therapists, psychologists, geriatricians, and activity leaders, volunteers and family members; an individualized approach for the resident. **Comparison:** 1 home, 24 residents. Usual care. | **QoL:** Qualidem  **QoC/health outcomes:** Activities of daily living | - Intervention did not demonstrate significant improvements in ADL or QoL. - After a 12-months, residents who received the intervention showed higher scores on positive self-image compared to the control group. |
| **Moyle 2016,** Australia  RCT/non-RCT  *G. Imp. Strat. to support multi-disciplinary teams* | **Inclusion:** Residents with confirmed diagnosis of dementia, score of 18 to 27 on the Mini-Mental State Examination, or a score of 3 on the Psychogeriatric Assessment Scales – Cognitive Impairment Scale.  **Profit status of NH**: Not for profit  **Intervention**: 3 LTC facilities, 51 staff, 37 family members. Face to face didactic education component focused on the CMDC, care planning, review of current practice, and dementia.  **Comparison**: 1 LTC facility, 30 staff, 11 family members. A single session of 4 hours of education in person-centered care. | **QoL:** Quality of life - Alzheimer's Disease (QOL-AD)  **QoC/health outcomes**: cognitive functioning | - Results suggest that the CMDC appears to be an effective evidence-based model of LTC facility dementia care, which can successfully contribute to improvements in QOL for people with dementia, and the development of more positive attitudes and work experiences of LTC facility staff. |

## Section 3: Healthcare Services Delivery: multidisciplinary studies

### Table A&B: Specialist & Primary care

| **Author Year**  **Study design**  **Country** | **Participants, Intervention, Comparison, Outcomes (PICO)** | **Outcomes** | **Main conclusions** |
| --- | --- | --- | --- |
| **Overall care** | | | |
| **Cordato 2018,** Australia  RCT/non-RCT  *E. Preventing acute care hospital admissions or readmissions* | **Inclusion:** Permanent residents of one of the participating NHs who had a hospital admission to the Department of Aged Care of St George Hospital during the study enrolment period.  **Profit status of NH:** NR  **Intervention**: 21 homes, 22 residents. Regular Early Assessment Post-Discharge (REAP) protocol of coordinated care, consisting of usual post-discharge care (as for the control group) in addition to regular conjoint geriatrician and NP evaluations in the participant’s usual place of residence, for a period of 6 months.  **Comparison:** 21 homes, 21 residents. Usual post-discharge care administered by their usual general practitioner (or primary care physician) and nursing staff at their NH. | **QoC/health outcomes:** hospital readmissions and inpatient days; ED episodes of care; readmitted for same diagnosis as screening admission; days from screening admission to readmission; deaths; acute diagnoses precipitating admission at baseline and re-admission: fall ±fracture, pneumonia, urinary tract infection, sepsis other, GI disturbance, exacerbation of congestive cardiac failure/ COPD, pressure area/ limb ischemia, concurrent delirium | - Findings demonstrate that with implementation of REAP, significant, cost-effective reductions in hospital readmissions and utilization of other medical and related services over 6 months can be achieved. |
| **palliative care** | | | |
| **Miller 2016,** USA  Cohort  *E. Preventing acute care hospital admissions or readmissions* | **Inclusion**: NH decedents in 2006–10, who were Medicare-eligible and not enrolled in Medicare Advantage in the last year of life  **Profit status of NH**: Mix of for profit and not for profit.  **Intervention**: 46 nursing homes, 477 residents. Receipt of an initial palliative care consult within 180 days of death.  **Comparison**: 46 nursing homes (same NHs as intervention), 1174 residents. Decedents eligible to serve as controls came from the same NHs and time frames, but did not receive a palliative care consult. | **QoC/health outcomes:** hospitalization in last: 7 days of life, 30 days of life, 60 days of life; ER visits in last: 30 days of life, 60 days of life; days between death and initial PC visit; rate of potentially burdensome transitions | - The first empirical evidence of the value of palliative care consults provided in NHs. - Shows a robust association between NH residents’ receipt of consults and lower rates of end-of-life acute care use and potentially burdensome care transitions. - Suggests Medicare and provider policy supporting concurrent specialty palliative care consults in NHs may lead to reductions in costly and often unsettling and discretionary hospital use. |
| **Miller 2017,** USA  Cohort  *E. Preventing acute care hospital admissions or readmissions* | **Inclusion:** NH residents with moderate to very severe dementia (Cognitive Performance Scale score ≥3).  **Profit status of NH**: Mix of for profit and not for profit.  **Intervention**: 31 homes, 203 residents. Consults are ordered by physicians often at the request of NH staff or families; palliative care consultation visits are made by NP palliative care specialists, under the supervision of certified palliative care physicians; initial consultations typically include a review of diagnoses and prognoses and address symptom control needs; specialists also review advance directives, if available, and discuss goals of care. Family meetings are a key component of many consultations.  **Comparison:** 31 homes, 429 residents. Stratified into two treatment groups by days between the initial consultation and death to enable examination of the timing of palliative care: earlier initial consultations (31– 180 days prior to death) and later initial consultations (1–30 days prior to death). | **QoC/health outcomes:** any hospital or acute care use 7 and 30 days before death; (potentially) burdensome end-of-life transitions (i.e., hospital or hospice admission 3 days before death or two plus acute care transitions 30 days before death) | - Consultations appear to reduce acute care use and (potentially) burdensome transitions for dying residents with dementia. - Reductions were greater when consultations were earlier. |

### Table A&C: Specialist & Direct patient care

| **Author Year**  **Study design**  **Country** | **Participants, Intervention, Comparison, Outcomes (PICO)** | **Outcomes** | **Main conclusions** |
| --- | --- | --- | --- |
| **DEMENTIA CARE, INCLUDING AGITATION** | | | |
| **Rapp 2013,** Germany  RCT/non-RCT  *F. Specific conditions/ interventions* | **Inclusion:** Nursing home residents with dementia  **Profit status of NH:** NR  **Intervention:** 9 nursing homes, 163 residents. A complex guideline-based intervention: Association of Geriatric Psychiatry comprise the training of nursing home staff, including the implementation of structured clinical assessments, the implementation of nonpharmacological interventions, and the optimization of pharmacological interventions aimed at reducing behavioral symptoms in patients with dementia living in nursing homes.  **Comparison:** 9 nursing homes, 141 residents. Treatment as usual. | **QoC/health outcomes**: agitation symptoms; number of neuroleptics, cholinesterase inhibitors (ChEIs), and antidepressants prescribed; adverse events | - Complex guideline-based interventions are effective in reducing agitated and disruptive behavior in nursing home residents with dementia. - Increased prescription of ChEIs and antidepressants together with decreased neuroleptic prescription suggests an effect toward guideline-based pharmacotherapy. |
| **overall care** | | | |
| **Harvey 2014,** Australia  RCT/non-RCT  *E. Preventing acute care hospital admissions or readmissions* | **Inclusion:** Patients aged ≥65 years admitted to hospital from RCF and being discharged to RCF.  **Profit status of NH:** NR  **Intervention**: 45 facilities, 57 residents. Residential Care Intervention Program in the Elderly (RECIPE): team comprised two part-time geriatricians and an aged care nurse consultant; all patients were reviewed in the RCF within four days of discharge; at the first visit, a comprehensive assessment and a tailored care plan was developed; services were provided and patients were offered further visits for review of intercurrent illness; service also provided education and support to RCF staff and the patients’ primary care physician.  **Comparison:** 45 facilities, 59 residents. Usual care: managed by the treating medical unit according to standard hospital protocols and received standard discharge planning, with follow-up at the RCF by their primary care physician service. | **QoL:** Quality of Life-Alzheimer’s Disease; Abbreviated Mental Test Score or Mini Mental State Examination; Barthel Index; Short Zung Interviewer-assisted Depression Scale  **QoC/health outcomes:** mortality; number of bed-days; inpatient admission; number of need to attend medical outpatient clinics; hospital length of stay; number of medications as predictor factors | - It is feasible and acceptable to provide a post-discharge outreach service to frail older people living in RCFs. - The hypothesis that a post-discharge, multidisciplinary assessment and management program could reduce readmissions and improve quality of life could not be proven, in part related to the unexpectedly small sample size. |
| **Hutchinson 2015,** Australia  CBA/ITS  *E. Preventing acute care hospital admissions or readmissions* | **Inclusion:** Aged care facility residents at high risk of acute care readmission.  **Profit status of NH:** NR  **Intervention**: 73 homes, 1327 residents. Post-RECIPE (Residential Care Intervention Program in the Elderly) enrolment (4 years following enrolment); provides expert comprehensive assessment and management by geriatricians and aged care nurse specialists to RCF residents who are at imminent risk of requiring acute care management; includes comprehensive care planning, management of inter-current illness and rapid access to acute care substitution services; capacity to make referrals to other clinical disciplines.  **Comparison:** time series analysis. Pre-RECIPE enrolment; quarters 1–8 (2 years prior to enrolment) | **QoC/health outcomes:** acute healthcare utilization (measured by mean admission rate per patient, mean length of stay, mean annual bed days per patient and time from enrolment to first readmission); patient mortality | - This study demonstrates that a geriatrician-led outreach service for RCF patients who are at high risk of acute care readmission may have a significant impact on reducing acute hospital utilization rates. |

### Table A&D: Specialist & Allied health care

| **Author Year**  **Study design**  **Country** | **Participants, Intervention, Comparison, Outcomes (PICO)** | **Outcomes** | **Main conclusions** |
| --- | --- | --- | --- |
| **DEpression** | | | |
| **McSweeney 2012,** Australia  RCT/non-RCT | **Inclusion:** Residents with dementia, 60 years of age or older, proficient in English, and who screened positive for depression.  **Profit status of NH:** NR  **Intervention:** NR (20 aged care facilities in total but unclear how many received the intervention), 21 residents. The specialist mental health consultation involved the provision of multidisciplinary specialist consultation regarding the best-practice management of depression in dementia. Consultations involved individually tailored medical and psychosocial recommendations provided to care staff and general practitioners.  **Comparison:** NR, 23 residents. Participated in the assessment component of the study, but no advice was offered regarding the management of depression during the intervention phase; offered educative seminars concerning assessment and management of depression and dementia at the conclusion of the study. | **QoC/health outcomes:** depression | - Findings support the utility of specialist mental health consultation in improving outcomes for depressed residents with dementia. - Replication with a larger sample size would enable the individual contribution of psychosocial and medical interventions to be determined, and provide the necessary impetus to improve access to mental health consultation in older residential care settings. |
| **hip fracture** | | | |
| **Crotty 2019; Killington 2020,** Australia  RCT/non-RCT | **Inclusion:** People aged 70 years and older who were recovering from hip fracture surgery and were walking prior to hip fracture.  **Profit status of NH:** NR  **Intervention:** NR (participants recruited from 3 hospitals), 121 residents. 4-week ambulatory geriatric rehabilitation program (delivered in the NCF): received visits from a hospital outreach team who provided a Comprehensive Geriatrics Assessment, physiotherapy and nutritional assessment and care plan. The intervention was low intensity and involved 13 h of input.  **Comparison:** NR, 119 residents. Usual care. | **QoL:** DEMQOL, DEMQOL-Proxy, EuroQol five dimension–five level questionnaire (EQ-5D-5L)  **QoC/health outcomes:** physical dependency, cognition, confusion or delirium, depression, pain, nutrition, adverse events, deaths | - Showed improved mobility, nutritional status and survival compared to usual care at 4 weeks. - These improvements did not persist at one year but there were small quality of life gains at 12 months in the survivors. - The outreach rehabilitation program was not cost-effective. - Further studies could investigate whether a longer-term or NCF-based rehabilitation approach following hip fracture is cost-effective. |
| **Optimal/appropriate medication use** | | | |
| **Doernberg 2015,** USA  CBA/ITS | **Inclusion:** Subjects included any resident of the skilled nursing or subacute sections of these LTCFs being treated for UTI with an antibiotic at the time of the ID pharmacist visit each week.  **Profit status of NH:** NR  **Intervention:** 3 community LTC facilities, 104 residents. The antimicrobial stewardship program (ASP) team (infectious diseases (ID) pharmacist and ID physician) performed weekly prospective audit and feedback of consecutive prescriptions for UTI.  **Comparison:** N/A, 292 prescriptions. During the pre-intervention phase, baseline information on facility-level antimicrobial susceptibility patterns and antimicrobial utilization were collected from each LTCF. | **QoC/health outcomes:** antibiotic prescriptions, resistant organisms (Clostridium difficile, ceftriaxone-resistant Enterobacteriacaeae, fluoroquinolone-resistant Pseudomonas aeruoginosa, and vancomycin-resistant Enterococci) | - Weekly prospective audit and feedback antimicrobial stewardship program in three community LTCFs over 6 months resulted in antibiotic utilization decreases but many lost opportunities for intervention. |
| **Verrue 2012,** Belgium  RCT/non-RCT | **Inclusion:** Nursing home residents.  **Profit status of NH**: NR  **Intervention**: 1 home, 69 residents. Pharmacist-conducted medication review: for each resident, the clinical pharmacist prepared a record with clinical (obtained via GP) and pharmaceutical (taken from medication in NH) data; appropriateness of prescribing was determined, and recommendations to optimize treatment were formulated; pharmacist consulted these recommendations with a geriatrician, whereafter they were communicated to the treating GP who could accept or reject the recommendation.  **Comparison:** 1 home, 79 residents. Usual care. | **QoC/health outcomes:** appropriateness of prescribing; underuse; use of potentially harmful drugs in older adults (‘overuse’) | - The pharmacist-conducted medication review only modestly improved the appropriateness of prescribing. - May be attributed to the low implementation rate of the pharmacist recommendation. |
| **Overall care** | | | |
| **De Luca 2016(A),** Italy  RCT/non-RCT | **Inclusion:** Residents of the nursing homes Oasi S Antoni located in Furci Siculo and Casa Pia, in Messina, Sicily, Italy.  **Profit status of NH:** Not for profit.  **Intervention**: 2 homes, 32 residents. Monitored for vital signs by an electronic box which consisted of a pulse oximeter, aneroid sphygmomanometer, and electrocardiograph (3x week); received a weekly tele-consultation either by a neurologist or a psychologist.  **Comparison:** 2 homes, 27 residents. Standard care. | **QoL:** EUROQoL-5D  **QoC/health outcomes:** Activities of Daily Living; Instrument Activities of Daily Living scale; Geriatric Depression Scale; Bedford Alzheimer Nursing Severity Scale; Brief Psychiatric Rating Scale; heart rate; systolic artery pressure; diastolic artery pressure; SPO2 pulse oximetry; admission to health care service | - Telemedicine can be considered as an important tool in improving health and quality of life in the elderly living in nursing homes, and potentially reducing healthcare service access, hospitalization, and costs. |

### Table A&C&D: Specialist & Direct patient care & Allied health care

| **Author Year**  **Study design**  **Country** | **Participants, Intervention, Comparison, Outcomes (PICO)** | **Outcomes** | **Main conclusions** |
| --- | --- | --- | --- |
| **overall care** | | | |
| **Wu 2010(A),** Taiwan  RCT/non-RCT  *E. Preventing acute care hospital admissions or readmissions* | **Inclusion:** Severely disabled (mean Barthel index = 0) residents of LTCFs in Northern Taipei City.  **Profit status of NH:** For profit.  **Intervention**: 4 homes, 42 residents. Introduction of an interdisciplinary team that actively participated in the daily care with onsite staff; composed by a geriatrician, nurses, physical therapists, dietitians, and social workers, which is supported by a municipal hospital; team members actively visited the LTCF residents and had team meeting with staff on monthly basis.  **Comparison:** 3 homes, 32 residents. Usual nursing and personal care with some professional care (i.e., physician, physical therapist, and dietitian visits) when necessary. | **QoC/health outcomes:** Unplanned feeding tube replacement; unplanned urinary catheter replacement; ED visits; hospitalizations; pneumonia; urinary tract infection; serum levels of albumin and hemoglobin; mini nutrition assessment score | - The clinical effectiveness of integrated care model among severely disabled LTCF residents is minimal. - Further cost- effectiveness study of integrated care model among severely disabled LTCF residents is needed to provide optimal care in this setting. |

### Table B&C: Primary care & Direct patient care

| **Author Year**  **Study design**  **Country** | **Participants, Intervention, Comparison, Outcomes (PICO)** | **Outcomes** | **Main conclusions** |
| --- | --- | --- | --- |
| **overall care** | | | |
| **Grabowski 2014,** USA  Cohort  *E. Preventing acute care hospital admissions or readmissions* | **Inclusion:** Nursing home residents.  **Profit status of NH:** For profit.  **Intervention**: 6 homes, 178 residents. Off-hours physician coverage by a telemedicine service; training sessions for direct care staff members and physicians to prior to intervention; introducing into the NH a cart with equipment for two-way videoconferencing and a high-resolution camera for use in wound care; when off-hours medical problem occurred, a staff member brought the cart into the resident’s room and contacted the telemedicine service, staffed by a medical secretary, a RN, a NP, and a physician.  **Comparison:** 5 homes, 140 residents. On-call coverage (evening or weekend calls were directed to the covering physician in the group practice, with off-hours care typically provided by telephone from a remote location). | **QoC/health outcomes:** number of residents hospitalized | - Switching from on-call to telemedicine physician coverage during off hours could reduce hospitalizations and therefore generate cost savings to Medicare in excess of the facility’s investment in the service. - Savings were evident only at the study NHs that used the telemedicine service to a greater extent, compared to the other study facilities. |
| **Haines 2020,** Australia  RCT/non-RCT  *E. Preventing acute care hospital admissions or readmissions* | **Inclusion:** Residents of Bupa Aged Care residential facilities.  **Profit status of NH:** NR  **Intervention**: 15 facilities, NR residents. A clinical manager was appointed to support the GP (at care facility) in managing medical practice; a registered nurse-in-charge was designated, and a RN or endorsed enrolled nurse was selected as team leader for a small group of personal care attendants responsible for a “community” of residents.  **Comparison:** 15 homes, NR residents. Residents were seen by external GPs not directly linked with facility staff, facility care managers had predominantly administrative roles, registered nurses undertook medication rounds but provided little direct care other than more complex procedural care, and direct care was largely the responsibility of Certificate III qualified or enrolled nurses. | **QoC/health outcomes:** Occupied bed-days; falls; fall-related fractures; unplanned hospital transfers; unplanned hospital admissions; days in hospital; out-of-hours GP call-outs; total infections; UTI, GI, and respiratory; pressure areas; skin tears; patient/family complaints; episodes of resident aggression; deaths; medication errors; polypharmacy; medications per resident; psychotropics prescribed; “as required” medications prescribed; antibiotics prescribed  **Staff:** Staff members leaving per time block; facility staff satisfaction | - Recruiting GPs to work directly in RACF may reduce the burden of unplanned presentations to hospitals and increase the reporting of adverse events. - In-house GPs identified reductions of about 50% in the numbers of unplanned transfers and admissions of residents to hospitals, days in hospital for unplanned admissions, out-of-hours GP call-outs, and complaints by residents and their families, as well as reduced prescribing of “as required” medications - A significant rise in the number of falls reported, increased rates of infection and medication errors. |
| **McCarthy 2019,** UK  CBA/ITS  *E. Preventing acute care hospital admissions or readmissions* | **Inclusion:** Care home residents: an individual with an age of ≥80 who lived within the post-code area of the care homes in Gateshead.  **Profit status of NH:** NR  **Intervention**: 34 residential and nursing care homes with 1503 beds. The short term model of the Gateshead Enhanced Health in Care Homes: the vanguard’s purpose was to increase collaborative working and establish partnerships between health and care providers to improve the health and wellbeing of residents and thereby reduce pressure on primary, secondary and social care services.  **Comparison:** 34 residential and nursing care homes with 1503 beds. The long-term model of the Gateshead Enhanced Health in Care Homes. | **QoC/health outcomes:** secondary care use; total numbers of Accident & Emergency attendances; non-elective admissions (excluding ambulatory care); outpatient appointments; bed-days in secondary care use | - Findings regarding reductions in secondary care resource utilization are consistent with findings from other evidence regarding the impact of Enhanced Health in Care Homes. - These complex interventions may require longer time frames before changes occur e.g. through a phase transition where there are long periods with little change in outcomes then large sudden changes in these outcomes occur. |
| **Morciano 2020,** UK  RCT/non-RCT  *E. Preventing acute care hospital admissions or readmissions* | **Inclusion:** Residents of enhanced care home Vanguard sites or non-Vanguard sites with emergency admissions and bed-days.  **Profit status of NH:** Not for profit.  **Intervention**: 6 homes, NR residents. Vanguard sites aimed at improving the quality and the coordination of health, care and rehabilitation services by promoting collaborations of the different institutions involved.  **Comparison:** 196 homes, NR residents. An entire Clinical Commissioning Group (CCG) where there is no Vanguard, or a set of practices not part of a Vanguard site in a CCG where there is a Vanguard site. | **QoC/health outcomes:** Emergency admission rates; total bed-days | - Vanguard initiative slowed the rise in emergency admissions, especially in care home sites and in the third and final year of the programme. - The introduction of these Vanguard prototypes was not associated with an overall reduction in total bed-days. - Integrated care policies should not be relied upon to make large reductions in hospital activity in the short-run, especially for population-based models. |
| **Pedersen 2017,** Denmark  RCT/non-RCT  *E. Preventing acute care hospital admissions or readmissions* | **Inclusion:** Patients ≥75 years, acutely admitted to the ED with an admission diagnosis of: pneumonia, COPD, dehydration, delirium, constipation, anaemia, heart failure, urinary tract infection or other infections.  **Profit status of NH:** NR  **Intervention:** NR homes, 281 residents. Patients received a follow-up home visit from a geriatric team (doctor and nurse) the first working day after discharge from the hospital or geriatric ward; patients had the possibility to phone the team; a team nurse could visit to assist the NH staff in managing intravenous antibiotics or collecting of blood samples.  **Comparison:** NR homes, 287 residents. At discharge, patients were screened to determine the need for a follow-up visit from their GP and the municipal nurse. After screening, the GP decided if the home visit was required. The patients discharged from the ED received a telephone call from the geriatric department the first working day after discharge. When necessary, a short follow-up was done. | **QoC/health outcomes:** 30-day mortality, 90-day mortality | - An early geriatric follow-up visit after discharge is a safe way of reducing readmissions and length of stay among the geriatric patients acutely admitted to hospital. - Patients living in their own home can even benefit further form the intervention with a reduction in mortality. |
| **Pedersen 2018,** Denmark  RCT/non-RCT  *E. Preventing acute care hospital admissions or readmissions* | **Inclusion:** NH residents aged ≥75 years admitted to hospital with one of nine medical diagnoses.  **Profit status of NH:** Not for profit.  **Intervention**: 52 homes, 330 residents. See Pedersen 2017 (above).  **Comparison:** 318 residents. Usual care; patients offered a follow-up visit by their general practitioner 7–14 days after discharge if the GP thought it necessary. | **QoC/health outcomes:** hospital readmission; length of hospital stay; mortality 30 and 90 days after admission, activities of the Geriatric Department during the first week after discharge (e.g., person-to-person contact between the nursing home staff and the hospital staff, hospital-initiated changes in medication) | - Early follow-up visits by the multidisciplinary Geriatric Team reduced the number of readmissions among NH residents admitted to the ED with one of nine medical diagnoses. - Mortality within 30 and 90 days after admission was not affected by this intervention. |
| **palliative care** | | | |
| **Chapman 2018,** Australia  RCT/non-RCT  *E. Preventing acute care hospital admissions or readmissions* | **Inclusion:** Nursing home residents.  **Profit status of NH:** NR  **Intervention:** 4 residential care facilities, 104 residents. New monthly onsite clinical meeting known as the Palliative Care Needs Round; provided a structure to improve communication and relationships between specialist palliative care (SPC) and the residential facility through case-based education.  **Comparison:** N/A (historical control group from the same NHs), 173 residents. All residents who died during the 3-year period prior to the intervention starting; involved general practitioners referring residents to SPC if they were identified as having unmet palliative care needs on a case-by-case basis. | **QoC/health outcomes:** resident hospitalization, achievement of preferred place of death | - The integration of SPC with a palliative approach as delivered in residential facilities has been demonstrated in our data to reduce admission of residents to acute care facilities such as hospitals. |
| **Forbat 2020,** Australia  RCT/non-RCT  *E. Preventing acute care hospital admissions or readmissions* | **Inclusion:** Care home residents.  **Profit status of NH:** NR.  **Intervention:** 12 care homes, NR residents. Consisted of direct support (clinical work with residents) and indirect support in the form of ‘Needs Rounds’ (run by specialist palliative care staff (two nurse practitioners and a clinical nurse consultant, who had access to advice from palliative medicine specialists for clinical decision making)), including a checklist to guide practice; up to 10 residents who are at greatest risk of dying without a plan in place and who have a high symptom burden are discussed; discussion of residents frequently led to initiating case conferences (attended by the resident, general practitioner and care home staff); completion of ACP with resident input, management of current and anticipatory medicines, and identifying legally appointed alternate decision makers.  **Comparison:** Cross-over study, all 12 care homes received intervention. Usual care, consisted of the specialist palliative care clinicians providing ad hoc reactive clinical consultations when referred by facility staff. | **QoC/health outcomes:** length of stay in hospital; number of admissions; quality of death and dying; place of death; adverse events; completion of advance care plans and appointment of medical power of attorneys  **Staff:** staff confidence/capability | - Needs Rounds offer a robust proactive approach to reducing length of stay in hospital and number of hospitalizations, by focusing on those with greatest symptom burden, providing specialist clinical care, education and anticipatory planning, including access to medications needed at end of life. - Needs Rounds were also associated with staff perceptions that residents had a better quality of death and dying and increased staff self-reported perceptions of capability. |
| **Liu 2020,** Australia  RCT/non-RCT  *E. Preventing acute care hospital admissions or readmissions* | **Inclusion:** Care homes for older people in the Australian Capital Territory. All residents, with the exception of respite residents.  **Profit status of NH:** NR  **Intervention**: 12 homes, 1700 residents. Needs Rounds (as described above in Forbat 2020).  **Comparison:** Cross-over study, all 12 care homes received intervention. Usual care consisted of the specialist palliative care clinicians providing ad-hoc clinical consultations when requested by facility staff or general practitioners. | **QoL:** Quality of Death and Dying Inventory short form (QODD)  **QoC/health outcomes:** length of stay in hospital; completion of advance care plans and appointment of medical power of attorneys.  **Staff:** staff self-reported capability and confidence using Capacity to Adopt a Palliative Approach (CAPA) tool | - Demonstrated improvements in quality of death and dying from a specialist palliative care outreach approach to supporting care home residents. - Needs Rounds act as a substantial tool to meet this recognized international marker of quality end-of-life experience. - Effective in improving staff awareness and capability in supporting residents in their final months of life. |

### Table B&D: Direct patient care & Allied health care

| **Author Year**  **Study design**  **Country** | **Participants, Intervention, Comparison, Outcomes (PICO)** | **Outcomes** | **Main conclusions** |
| --- | --- | --- | --- |
| **optimal/appropriate medication use** | | | |
| **Azermai 2017,** Belgium  CBA/ITS  *G. Imp. Strategies to support multi-disciplinary teams* | **Inclusion:** All consenting nursing home residents residing in the nursing home at time of the baseline collection, were included.  **Profit status of NH:** NR  **Intervention**: 1 nursing home, 118 residents. Three educational courses at the start covering sleeping problems (focusing on hypnosedatives), old age depression (focusing on antidepressants) and on behavioural problems (focusing on antipsychotic agents). The main intervention was the transition to person-centered care, through professional support.  **Comparison**: 1 nursing home, 275 residents. Same educational courses as intervention group. There was no transition towards person-centered care. | **QoC/health outcomes:** mean medication use, polypharmacy, nervous system drugs, alimentary medications, cardiovascular medications, blood medications, respiratory medications, musculo-skeletal medications, psychotropic drug use (hypno-sedative, antidepressant, antipsychotics) | - Management of common behavioural and psychological problems in older adults (e.g. sleeping problems, depression, agitation) requires a multidisciplinary approach. - A transition towards a person-centred care to a significant decrease in the use of psychotropic drugs, even after 1-year follow-up. - Education only had a limited effect. - Although areas of uncertainty remain, this quality improvement project has the potential to be employed as a guideline in other nursing homes. |

## Bibliography of included studies

1. Arendts G, Deans P, O’Brien K, Etherton-Beer C, Howard K, Lewin G, et al. A clinical trial of nurse practitioner care in residential aged care facilities. Arch Gerontol Geriatr. 2018;77:129–32.

2. Azermai M, Wauters M, De Meester D, Renson L, Pauwels D, Peeters L, et al. A quality improvement initiative on the use of psychotropic drugs in nursing homes in Flanders. Acta Clin Belg Int J Clin Lab Med. 2017;72(3):163–71.

3. Balsom C, Pittman N, King R, Kelly D. Impact of a pharmacist-administered deprescribing intervention on nursing home residents: a randomized controlled trial. Int J Clin Pharm. 2019;03:03.

4. Barbe AG, Kottmann HE, Derman SHM, Noack MJ. Efficacy of regular professional brushing by a dental nurse for 3 months in nursing home residents-A randomized, controlled clinical trial. Int J Dent Hyg. 2019;17(4):327–35.

5. Barbe AG, Kupeli LS, Hamacher S, Noack MJ. Impact of regular professional toothbrushing on oral health, related quality of life, and nutritional and cognitive status in nursing home residents. Int J Dent Hyg. 2020;18(3):238–50.

6. Beaupre LA, Lier D, Magaziner JS, Jones CA, Johnston DWC, Wilson DM, et al. An Outreach Rehabilitation Program for Nursing Home Residents after Hip Fracture may be Cost-Saving. J Gerontol A Biol Sci Med Sci. 2020;75(10):e159–65.

7. Beck AM, Christensen AG, Hansen BS, Damsbo-Svendsen S, Moller TK. Multidisciplinary nutritional support for undernutrition in nursing home and home-care: A cluster randomized controlled trial. Nutrition. 2016;32(2):199–205.

8. Boorsma M, Frijters DH, Knol DL, Ribbe ME, Nijpels G, van Hout HP. Effects of multidisciplinary integrated care on quality of care in residential care facilities for elderly people: a cluster randomized trial. CMAJ Can Med Assoc J. 2011;183(11):E724-732.

9. Borbasi S, Emmanuel E, Farrelly B, Ashcroft J. Report of an evaluation of a nurse-led dementia outreach service for people with the behavioural and psychological symptoms of dementia living in residential aged care facilities. Perspect Public Health. 2011;131(3):124–30.

10. Boyd M, Armstrong D, Parker J, Pilcher C, Zhou L, McKenzie-Green B, et al. Do gerontology nurse specialists make a difference in hospitalization of long-term care residents? Results of a randomized comparison trial. J Am Geriatr Soc. 2014;62(10):1962–7.

11. Brett L, Stapley P, Meedya S, Traynor V. Effect of physical exercise on physical performance and fall incidents of individuals living with dementia in nursing homes: a randomized controlled trial. Physiother Theory Pract. 2019;1–14.

12. Chapman M, Johnston N, Lovell C, Forbat L, Liu WM. Avoiding costly hospitalisation at end of life: findings from a specialist palliative care pilot in residential care for older adults. BMJ Support Palliat Care. 2018;8(1):102–9.

13. Connolly MJ, Boyd M, Broad JB, Kerse N, Lumley T, Whitehead N, et al. The Aged Residential Care Healthcare Utilization Study (ARCHUS): a multidisciplinary, cluster randomized controlled trial designed to reduce acute avoidable hospitalizations from long-term care facilities. J Am Med Dir Assoc. 2015;16(1):49–55.

14. Connolly MJ, Broad JB, Boyd M, Zhang TX, Kerse N, Foster S, et al. The “Big Five”. Hypothesis generation: a multidisciplinary intervention package reduces disease-specific hospitalisations from long-term care: a post hoc analysis of the ARCHUS cluster-randomised controlled trial. Age Ageing. 2016;45(3):415–20.

15. Connolly MJ, Broad JB, Bish T, Zhang X, Bramley D, Kerse N, et al. Reducing emergency presentations from long-term care: A before-and-after study of a multidisciplinary team intervention. Maturitas. 2018;117:45–50.

16. Conway J, Higgins I, Hullick C, Hewitt J, Dilworth S. Nurse-led ED support for residential aged care facility staff: an evaluation study. Int Emerg Nurs. 2015;23(2):190–6.

17. Cool C, Cestac P, McCambridge C, Rouch L, e Souto Barreto P, Rolland Y, et al. Reducing potentially inappropriate drug prescribing in nursing home residents: effectiveness of a geriatric intervention. Br J Clin Pharmacol. 2018;84(7):1598–610.

18. Cordato NJ, Kearns M, Smerdely P, Seeher KM, Gardiner MD, Brodaty H. Management of Nursing Home Residents Following Acute Hospitalization: Efficacy of the “Regular Early Assessment Post-Discharge (REAP)” Intervention. J Am Med Dir Assoc. 2018;19(3):276.e211-276.e219.

19. Craswell A, Wallis M, Coates K, Marsden E, Taylor A, Broadbent M, et al. Enhanced primary care provided by a nurse practitioner candidate to aged care facility residents: A mixed methods study. Collegian. 2020;27(3):281–7.

20. Crotty M, Killington M, Liu E, Cameron ID, Kurrle S, Kaambwa B, et al. Should we provide outreach rehabilitation to very old people living in Nursing Care Facilities after a hip fracture? A randomised controlled trial. Age Ageing. 2019;48(3):373–80.

21. D’Arcy LP, Stearns SC, Domino ME, Hanson LC, Weinberger M. Is geriatric care associated with less emergency department use? J Am Geriatr Soc. 2013;61(1):4–11.

22. De Luca R, Bramanti A, De Cola MC, Trifiletti A, Tomasello P, Torrisi M, et al. Tele-health-care in the elderly living in nursing home: the first Sicilian multimodal approach. Aging-Clin Exp Res. 2016;28(4):753–9.

23. de Souto Barreto P, Lapeyre-Mestre M, Cestac P, Vellas B, Rolland Y. Effects of a geriatric intervention aiming to improve quality care in nursing homes on benzodiazepine use and discontinuation. Br J Clin Pharmacol. 2016;81(4):759–67.

24. Doernberg SB, Dudas V, Trivedi KK. Implementation of an antimicrobial stewardship program targeting residents with urinary tract infections in three community long-term care facilities: a quasi-experimental study using time-series analysis. Antimicrob Resist Infect Control. 2015;4:54.

25. Dorfman R, London Z, Metias M, Kabakchiev B, Mukerjee G, Moser A. Individualized Medication Management in Ontario Long-Term Care Clinical Impact on Management of Depression, Pain, and Dementia. J Am Med Dir Assoc. 2020;21(6):823-829.e825.

26. El Haddad K, e Souto Barreto P, e Mazieres CL, Rolland Y. Effect of a geriatric intervention aiming to improve polypharmacy in nursing homes. Eur Geriatr Med. 2020;

27. Forbat L, Liu WM, Koerner J, Lam L, Samara J, Chapman M, et al. Reducing time in acute hospitals: A stepped-wedge randomised control trial of a specialist palliative care intervention in residential care homes. Palliat Med. 2020;34(5):571–9.

28. Gloth FM, 3rd G. A comparative effectiveness trial between a post-acute care hospitalist model and a community-based physician model of nursing home care. J Am Med Dir Assoc. 2011;12(5):384–6.

29. Grabowski DC, O’Malley AJ. Use of telemedicine can reduce hospitalizations of nursing home residents and generate savings for medicare. Health Aff (Millwood). 2014;33(2):244–50.

30. Guion V, De Souto Barreto P, Sourdet S, Rolland Y. Effect of an Educational and Organizational Intervention on Pain in Nursing Home Residents: A Nonrandomized Controlled Trial. J Am Med Dir Assoc. 2018;19(12):1118-1123.e1112.

31. Haines TP, Palmer AJ, Tierney P, Si L, Robinson AL. A new model of care and in-house general practitioners for residential aged care facilities: a stepped wedge, cluster randomised trial. Med J Aust. 2020;212(9):409–15.

32. Harvey P, Storer M, Berlowitz DJ, Jackson B, Hutchinson A, Lim WK. Feasibility and impact of a post-discharge geriatric evaluation and management service for patients from residential care: the Residential Care Intervention Program in the Elderly (RECIPE). BMC Geriatr. 2014;14:48.

33. Hashimoto R, Fujii K, Shimoji S, Utsumi A, Hosokawa K, Tochino H, et al. Study of pharmacist intervention in polypharmacy among older patients: Non-randomized, controlled trial. Geriatr Gerontol Int. 2020;20(3):229–37.

34. Henskens M, Nauta IM, Scherder EJA, Oosterveld FGJ, Vrijkotte S. Implementation and effects of Movement-oriented Restorative Care in a nursing home - a quasi-experimental study. BMC Geriatr. 2017;17:1–11.

35. Hewitt J, Saing S, Goodall S, Henwood T, Clemson L, Refshauge K. An economic evaluation of the SUNBEAM programme: a falls-prevention randomized controlled trial in residential aged care. Clin Rehabil. 2019;33(3):524–34.

36. Hopper T, Slaughter SE, Hodgetts B, Ostevik A, Ickert C. Hearing loss and cognitive-communication test performance of long-term care residents with dementia: Effects of amplification. [References]. J Speech Lang Hear Res. 2016;59(6):1533–42.

37. Hullick C, Conway J, Higgins I, Hewitt J, Dilworth S, Holliday E, et al. Emergency department transfers and hospital admissions from residential aged care facilities: a controlled pre-post design study. BMC Geriatr. 2016;16:102.

38. Hutchinson AF, Parikh S, Tacey M, Harvey PA, Lim WK. A longitudinal cohort study evaluating the impact of a geriatrician-led residential care outreach service on acute healthcare utilisation. Age Ageing. 2015;44(3):365–70.

39. Justine M, Hamid TA, Kamalden TFT, Ahmad Z. A multicomponent exercise program’s effects on health-related quality of life of institutionalized elderly. Top Geriatr Rehabil. 2010;26(1):70–9.

40. Kaasalainen S, Wickson-Griffiths A, Akhtar-Danesh N, Brazil K, Donald F, Martin-Misener R, et al. The effectiveness of a nurse practitioner-led pain management team in long-term care: A mixed methods study. Int J Nurs Stud. 2016;62:156–67.

41. Kane RL, Huckfeldt P, Tappen R, Engstrom G, Rojido C, Newman D, et al. Effects of an Intervention to Reduce Hospitalizations From Nursing Homes: A Randomized Implementation Trial of the INTERACT Program. JAMA Intern Med. 2017;177(9):1257–64.

42. Killington M, Davies O, Crotty M, Crane R, Pratt N, Mills K, et al. People living in nursing care facilities who are ambulant and fracture their hips: description of usual care and an alternative rehabilitation pathway. BMC Geriatr. 2020;20(1):128.

43. Kobewka DM, Kunkel E, Hsu A, Talarico R, Tanuseputro P. Physician Availability in Long-Term Care and Resident Hospital Transfer: A Retrospective Cohort Study. J Am Med Dir Assoc. 2020;21(4):469-475.e1.

44. Kulakci H, Emiroglu ON. Impact of nursing care services on self-efficacy perceptions and healthy lifestyle behaviors of nursing home residents. Res Gerontol Nurs. 2013;6(4):242–52.

45. Lacny S, Zarrabi M, Martin-Misener R, Donald F, Sketris I, Murphy AL, et al. Cost-effectiveness of a nurse practitioner-family physician model of care in a nursing home: controlled before and after study. J Adv Nurs. 2016;72(9):2138–52.

46. Laffon de Mazieres C, Lapeyre-Mestre M, Vellas B, e Souto Barreto P, Rolland Y. Impact of a geriatric intervention conducted in nursing homes on inappropriate prescriptions of antipsychotics. Eur Geriatr Med. 2019;10(2):285–93.

47. Lin WY, Huang HY, Liu CS, Li CI, Lee SD, Lin CC, et al. A hospital-based multidisciplinary approach improves nutritional status of the elderly living in long-term care facilities in middle Taiwan. Arch Gerontol Geriatr. 2010;50(Suppl 1):S22-26.

48. Lindelof N, Rosendahl E, Gustafsson S, Nygaard J, Gustafson Y, Nyberg L. Perceptions of participating in high-intensity functional exercise among older people dependent in activities of daily living (ADL). Arch Gerontol Geriatr. 2013;57(3):369–76.

49. Liu WM, Koerner J, Lam L, Johnston N, Samara J, Chapman M, et al. Improved Quality of Death and Dying in Care Homes: A Palliative Care Stepped Wedge Randomized Control Trial in Australia. J Am Geriatr Soc. 2020;68(2):305–12.

50. Man REK, Gan ATL, Constantinou M, Fenwick EK, Holloway E, Finkelstein EA, et al. Effectiveness of an innovative and comprehensive eye care model for individuals in residential care facilities: results of the residential ocular care (ROC) multicentred randomised controlled trial. Br J Ophthalmol. 2020;

51. Marchini L, Recker E, Hartshorn J, Cowen H, Lynch D, Drake D, et al. Iowa nursing facility oral hygiene (INFOH) intervention: A clinical and microbiological pilot randomized trial. Spec Care Dentist. 2018;38(6):345–55.

52. McCarthy A, McMeekin P, Haining S, Bainbridge L, Laing C, Gray J. Rapid evaluation for health and social care innovations: challenges for “quick wins” using interrupted time series. BMC Health Serv Res. 2019;19(1):964.

53. McDerby N, Kosari S, Bail K, Shield A, Peterson G, Naunton M. The effect of a residential care pharmacist on medication administration practices in aged care: A controlled trial. J Clin Pharm Ther. 2019;44(4):595–602.

54. McDerby N, Kosari S, Bail K, Shield A, Peterson G, Naunton M. Residential Aged Care Pharmacist: An Australian Pilot Trial Exploring the Impact on Quality Use of Medicines Indicators. Medicines. 2020;7(4):20.

55. McSweeney K, Jeffreys A, Griffith J, Plakiotis C, Kharsas R, O’Connor DW. Specialist mental health consultation for depression in Australian aged care residents with dementia: a cluster randomized trial. Int J Geriatr Psychiatry. 2012;27(11):1163–71.

56. Miller SC, Lima JC, Intrator O, Martin E, Bull J, Hanson LC. Palliative Care Consultations in Nursing Homes and Reductions in Acute Care Use and Potentially Burdensome End-of-Life Transitions. J Am Geriatr Soc. 2016;64(11):2280–7.

57. Miller SC, Lima JC, Intrator O, Martin E, Bull J, Hanson LC. Specialty Palliative Care Consultations for Nursing Home Residents With Dementia. J Pain Symptom Manage. 2017;54(1):9-16.e5.

58. Morciano M, Checkland K, Billings J, Coleman A, Stokes J, Tallack C, et al. New integrated care models in England associated with small reduction in hospital admissions in longer-term: A difference-in-differences analysis. Health Policy. 2020;124(8):826–33.

59. Morino T, Ookawa K, Haruta N, Hagiwara Y, Seki M. Effects of professional oral health care on elderly: randomized trial. Int J Dent Hyg. 2014;12(4):291–7.

60. Moyle W, Cooke ML, Beattie E, Shum DH, O’Dwyer ST, Barrett S, et al. Foot massage and physiological stress in people with dementia: a randomized controlled trial. J Altern Complement Med N Y N. 2014;20(4):305–11.

61. Moyle W, Cooke ML, Beattie E, Shum DHK, O’Dwyer ST, Barrett S. Foot massage versus quiet presence on agitation and mood in people with dementia: A randomised controlled trial. Int J Nurs Stud. 2014;51(6):856–64.

62. Moyle W, Venturato L, Cooke M, Murfield J, Griffiths S, Hughes J, et al. Evaluating the capabilities model of dementia care: a non-randomized controlled trial exploring resident quality of life and care staff attitudes and experiences. Int Psychogeriatr. 2016;28(7):1091–100.

63. Nishiyama Y, Inaba E, Uematsu H, Senpuku H. Effects of mucosal care on oral pathogens in professional oral hygiene to the elderly. Arch Gerontol Geriatr. 2010;51(3):e139-143.

64. Patterson SM, Hughes CM, Crealey G, Cardwell C, Lapane KL. An evaluation of an adapted U.S. model of pharmaceutical care to improve psychoactive prescribing for nursing home residents in northern ireland (fleetwood northern ireland study). J Am Geriatr Soc. 2010;58(1):44–53.

65. Pedersen LH, Gregersen M, Barat I, Damsgaard EM. Early geriatric follow-up after discharge reduces mortality among patients living in their own home. A randomised controlled trial. Eur Geriatr Med. 2017;8(4):330–6.

66. Pedersen LH, Gregersen M, Barat I, Damsgaard EM. Early geriatric follow-up visits to nursing home residents reduce the number of readmissions: a quasi-randomised controlled trial. Eur Geriatr Med. 2018;9(3):329–37.

67. Rantz MJ, Popejoy L, Vogelsmeier A, Galambos C, Alexander G, Flesner M, et al. Impact of Advanced Practice Registered Nurses on Quality Measures: The Missouri Quality Initiative Experience. J Am Med Dir Assoc. 2018;19(6):541–50.

68. Rapp MA, Mell T, Majic T, Treusch Y, Nordheim J, Niemann-Mirmehdi M, et al. Agitation in nursing home residents with dementia (VIDEANT trial): effects of a cluster-randomized, controlled, guideline implementation trial. J Am Med Dir Assoc. 2013;14(9):690–5.

69. Rodriguez-Mansilla J, Gonzalez-Lopez-Arza MV, Varela-Donoso E, Montanero-Fernandez J, Jimenez-Palomares M, Garrido-Ardila EM. Ear therapy and massage therapy in the elderly with dementia: a pilot study. J Tradit Chin Med. 2013;33(4):461–7.

70. Rolland Y, Mathieu C, Piau C, Cayla F, Bouget C, Vellas B, et al. Improving the Quality of Care of Long-Stay Nursing Home Residents in France. J Am Geriatr Soc. 2016;64(1):193–9.

71. Rolland Y, Tavassoli N, De Souto Barreto P, Perrin A, Laffon De Mazieres C, Rapp T, et al. Systematic Dementia Screening by Multidisciplinary Team Meetings in Nursing Homes for Reducing Emergency Department Transfers: the IDEM Cluster Randomized Clinical Trial. JAMA Netw Open. 2020;3(2).

72. Sackley CM, Walker MF, Burton CR, Watkins CL, Mant J, Roalfe AK, et al. An Occupational Therapy intervention for residents with stroke-related disabilities in UK Care Homes (OTCH): cluster randomised controlled trial with economic evaluation. Health Technol Assess Winch Engl. 2016;20(15):1–138.

73. Seleskog B, Lindqvist L, Wardh I, Engstrom A, von Bultzingslowen I. Theoretical and hands-on guidance from dental hygienists promotes good oral health in elderly people living in nursing homes, a pilot study. Int J Dent Hyg. 2018;16(4):476–83.

74. Sluggett JK, Chen EYH, Ilomaki J, Corlis M, Van Emden J, Hogan M, et al. Reducing the Burden of Complex Medication Regimens: SImplification of Medications Prescribed to Long-tErm care Residents (SIMPLER) Cluster Randomized Controlled Trial. J Am Med Dir Assoc. 2020;21(8).

75. Snider KT, Snider EJ, Johnson JC, Hagan C, Schoenwald C. Preventative osteopathic manipulative treatment and the elderly nursing home resident: a pilot study. J Am Osteopath Assoc. 2012;112(8):489–501.

76. Sumi Y, Ozawa N, Miura H, Michiwaki Y, Umemura O. Oral care help to maintain nutritional status in frail older people. Arch Gerontol Geriatr. 2010;51(2):125–8.

77. Telenius EW, Engedal K, Bergland A. Effect of a high-intensity exercise program on physical function and mental health in nursing home residents with dementia: an assessor blinded randomized controlled trial. PLoS ONE Electron Resour. 2015;10(5):e0126102.

78. Telenius EW, Engedal K, Bergland A. Long-term effects of a 12 weeks high-intensity functional exercise program on physical function and mental health in nursing home residents with dementia: a single blinded randomized controlled trial. BMC Geriatr. 2015;15(158).

79. Temkin-Greener H, Ladwig S, Ye Z, Norton SA, Mukamel DB. Improving palliative care through teamwork (IMPACTT) in nursing homes: Study design and baseline findings. Contemp Clin Trials. 2017;56:1–8.

80. Temkin-Greener H, Mukamel DB, Ladd H, Ladwig S, Caprio TV, Norton SA, et al. Impact of Nursing Home Palliative Care Teams on End-of-Life Outcomes: A Randomized Controlled Trial. Med Care. 2018;56(1):11–8.

81. Torma J, Winblad U, Saletti A, Cederholm T. Strategies to implement community guidelines on nutrition and their long-term clinical effects in nursing home residents. J Nutr Health Aging. 2015;19(1):70–6.

82. Travers C. Increasing enjoyable activities to treat depression in nursing home residents with dementia: a pilot study. Dement Lond Engl. 2017;16(2):204–18.

83. Tynan A, Deeth L, McKenzie D. An integrated oral health program for rural residential aged care facilities: a mixed methods comparative study. BMC Health Serv Res. 2018;18(1):515.

84. Verkaik R, Francke AL, van Meijel B, Spreeuwenberg PM, Ribbe MW, Bensing JM. The effects of a nursing guideline on depression in psychogeriatric nursing home residents with dementia. Int J Geriatr Psychiatry. 2011;26(7):723–32.

85. Verrue C, Mehuys E, Boussery K, Adriaens E, Remon JP, Petrovic M. A pharmacist-conducted medication review in nursing home residents: impact on the appropriateness of prescribing. Acta Clin Belg. 2012;67(6):423–9.

86. Weatherall CD, Hansen AT, Nicholson S. The effect of assigning dedicated general practitioners to nursing homes. Health Serv Res. 2019;54(3):547–54.

87. Wenborn J, Challis D, Head J, Miranda-Castillo C, Popham C, Thakur R, et al. Providing activity for people with dementia in care homes: a cluster randomised controlled trial. Int J Geriatr Psychiatry. 2013;28(12):1296–304.

88. Wikstrom M, Kareem KL, Almstahl A, Palmgren E, Lingstrom P, Wardh I. Effect of 12-month weekly professional oral hygiene care on the composition of the oral flora in dentate, dependent elderly residents: A prospective study. Gerodontology. 2017;34(2):240–8.

89. Wu MP, Lin PF, Lin KJ, Sun RS, Yu WR, Peng LN, et al. Integrated care for severely disabled long-term care facility residents: is it better? Arch Gerontol Geriatr. 2010;50(3):315–8.

90. Wylie G, Menz HB, McFarlane S, Ogston S, Sullivan F, Williams B, et al. Podiatry intervention versus usual care to prevent falls in care homes: pilot randomised controlled trial (the PIRFECT study). BMC Geriatr. 2017;17(1):143.

91. Zenthofer A, Dieke R, Dieke A, Wege KC, Rammelsberg P, Hassel AJ. Improving oral hygiene in the long-term care of the elderly--a RCT. Community Dent Oral Epidemiol. 2013;41(3):261–8.

92. Zenthofer A, Meyer-Kuhling I, Hufeland AL, Schroder J, Cabrera T, Baumgart D, et al. Carers’ education improves oral health of older people suffering from dementia - results of an intervention study. Clin Interv Aging. 2016;11:1755–62.
